# Structural Modifications and Biological Evaluations of Rift Valley Fever Virus Inhibitors Identified from Chemical Library Screening

**DOI:** 10.1021/acsomega.1c06513

**Published:** 2022-02-16

**Authors:** Koushikul Islam, Marcus Carlsson, Per-Anders Enquist, Weixing Qian, Marko Marttila, Mårten Strand, Clas Ahlm, Magnus Evander

**Affiliations:** † Department of Clinical Microbiology, Umeå University, Umeå 901 85, Sweden; ‡ Department of Chemistry, Umeå University, Umeå 901 87, Sweden

## Abstract

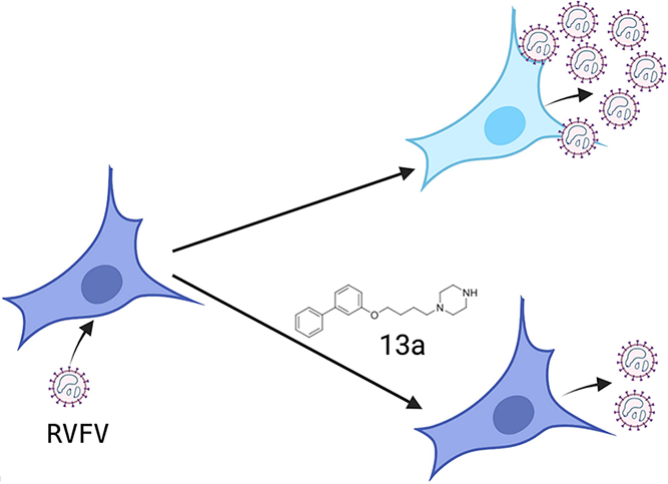

The Rift Valley fever virus (RVFV) is an emerging high-priority pathogen endemic in Africa with pandemic potential. There is no specific treatment or approved antiviral drugs for the RVFV. We previously developed a cell-based high-throughput assay to screen small molecules targeting the RVFV and identified a potential effective antiviral compound (1-*N*-(2-(biphenyl-4-yloxy)ethyl)propane-1,3-diamine) as a lead compound. Here, we investigated how structural modifications of the lead compound affected the biological properties and the antiviral effect against the RVFV. We found that the length of the 2-(3-aminopropylamino)ethyl chain of the compound was important for the compound to retain its antiviral activity. The antiviral activity was similar when the 2-(3-aminopropylamino)ethyl chain was replaced with a butyl piperazine chain. However, we could improve the cytotoxicity profile of the lead compound by changing the phenyl piperazine linker from the *para*-position (compound **9a**) to the *meta*-position (compound **13a**). Results from time-of-addition studies suggested that compound **13a** might be active during virus post-entry and/or the replication phase of the virus life cycle and seemed to affect the K^+^ channel. The modifications improved the properties of our lead compound, and our data suggest that **13a** is a promising candidate to evaluate further as a therapeutic agent for RVFV infection.

## Introduction

Rift Valley fever (RVF) is an acute viral infection caused by the emerging mosquito-borne Rift Valley fever virus (RVFV) (genus *Phlebovirus*, family *Phenuiviridae*), which infects domestic animals and humans. The RVFV causes deadly infection among ruminants with high fever, hepatitis, acute deaths of newborns, and abortions in pregnant animals. Abortion storms are considered as a hallmark of RVFV outbreaks.^1−3^ Humans are infected by mosquito bites as well as handling contaminated animal tissues and fluids while working with slaughter or taking care of infected animals in herds. In humans, RVFV infection ranges from a mild illness associated with fever and liver abnormalities to much more severe symptoms such as retinitis, encephalitis, and hemorrhagic fever.^4,5^ The association of RVFV infection with miscarriage in humans has also been reported.^6,7^

The RVFV causes recurrent outbreaks throughout the African countries.^8^ The RVF epidemic in Yemen and Saudi Arabia in 2000 was the first of this kind outside Africa. This epidemic affected both livestock and humans, and approximately 200 humans died.^9,10^ The spread of the RVFV outside Africa was mainly due to import of infected animals from epidemic countries. Notably, more than 30 different mosquito species have been identified to carry the RVFV, of which several are competent vectors and are distributed globally.^11^ The RVFV has been endemic in the African subcontinent for decades, but outbreaks in Saudi Arabia, Yemen, Madagascar, and the Comoros Archipelago suggest that the geographical distribution of the RVFV is changing.^12−14^ Recent studies have reported the presence of RVFV-seropositive animals in Iran and Turkey,^15,16^ and other Asian countries are also at risk. Due to the presence of competent mosquito vectors in Europe, studies have emphasized that the RVFV can pose a major threat there.^17−19^

Currently, there are no safe and effective treatments to prevent or cure RVFV-infected humans or livestock. So far, several natural products and synthetic chemical compounds have been reported as potent RVFV inhibitors *in vitro*, but none of them progressed further to become an RVFV-specific drug candidate.^20−26^ Favipiravir is not RVFV-specific but has broad-spectrum activity against a number of RNA viruses, including the RVFV, and has gone through human clinical trials.^27−29^ However, there is a need to develop potent efficacious antiviral compounds against RVFV infection.

**Figure 1:**
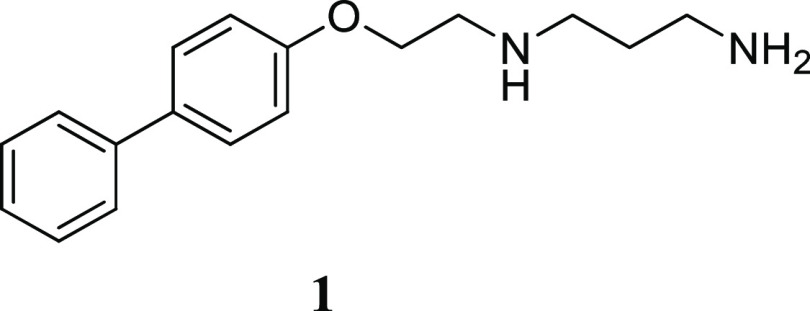
Structure of parent compound **1**, identified from high-throughput screening of RVFV infection *in vitro*.

Previously, we have developed a high-throughput screening method for identifying potent inhibitors of RVFV infection *in vitro* and identified several compounds.^30^ The parent compound (*N*^1^-(2-(biphenyl-4-yloxy)ethyl)propane-1,3-diamine) was identified as a promising hit for further evaluation (Figure 1). The structure of the parent compound (designated as compound **1**) consists of a biphenyl group that is connected, through an ether bond, to a 2-(3-aminopropylamino)ethyl chain. To investigate the important structural factors of parent compound **1**, we aimed to synthesize a set of first-generation compounds with modification of the 2-(3-aminopropylamino)ethyl chain and keep the biphenyloxy part conserved. Depending on the result after the first investigation, the intention was to investigate the biphenyl part by synthesizing a set of second-generation compounds with modifications of the biphenyl part and keep the modified alkyl amine chain of interest conserved.

## Results and Discussion

### Chemistry: First-Generation Compounds

**Scheme 1:**
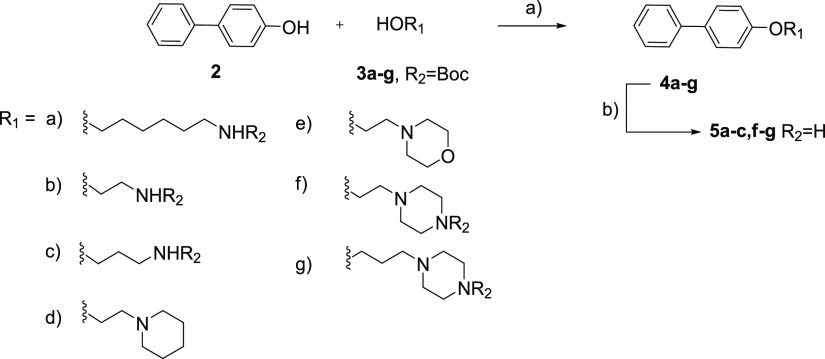
Synthesis of Compounds **4d**–**e**, **5a**–**c**, and **5f**–**g**: (a) PPh_3_, DIAD, THF, RT; (b) **4a**–**c**, TFA, DCM at RT; **4f**–**g**, 4 M HCl (dioxane), DCM, RT

To determine the importance of the 2-(3-aminopropylamino)ethyl chain for the parent compound **1**, a set of first-generation compounds were synthesized. The approach for synthesizing the compounds **4d**–**e**, **5a**–**c**, and **5f**–**g** is described in Scheme 1. Ether bond formation between alcohols **3a**–**g** and 4-biphenol **2** was performed under standard Mitsunobu conditions using di-isopropyl azodicarboxylate (DIAD) and triphenylphosphine (PPh_3_) in tetrahydrofuran (THF), which gave compounds **4a**–**g**.^31^ N-Boc removal of N-Boc-protected compounds **4a**–**c** and **4f**–**g** was performed under acidic conditions with either trifluoroacetic acid (TFA) or HCl, which gave the corresponding deprotected compounds **5a**–**c** and **5f**–**g** as free amines or HCl salts.

**Scheme 2:**
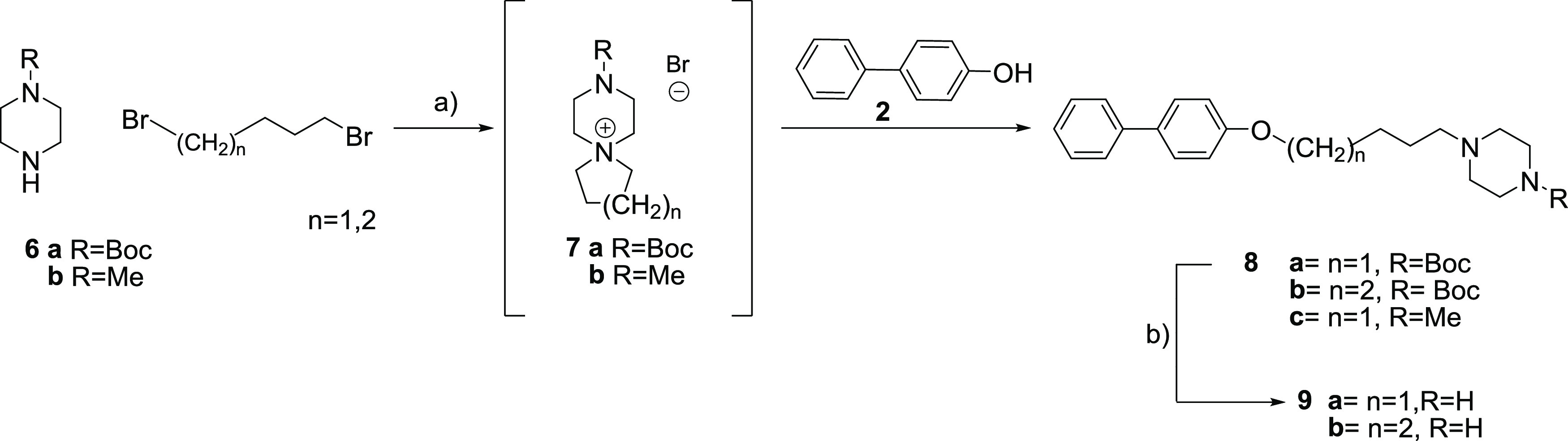
One-Pot Synthesis of Compounds **8a**–**c** and Deprotection of the Boc Group: (a) Cs_2_CO_3_, 1,4-Dibromobutane, MeCN, Microwave Heating (115 °C) Followed by Addition of 4-Biphenol **2** and Microwave Heating (170 °C); (b) 4 M HCl (dioxane), DCM

The synthetic approach for alkyl-piperazine analogs **8c** and **9a**–**b** is described in Scheme 2. For synthesis of compounds **8a**–**c**, N-Boc piperazine **6a** or *N*-methyl piperazine **6b** was treated with an equivalent amount of 1,4-dibromobutane or 1,5-dibromopentane in acetonitrile and excess cesium carbonate as a base. Increasing the temperature to 115 °C for 20 min using microwave heating allowed the spiro salt intermediate **7a**–**7c** to form *in situ*. Addition of 4-biphenol **2** in small excess and increased microwave heating to 170 °C resulted in nucleophilic ring opening of the spiro salt intermediate, which gave the alkyl piperazine analogs **8a**–**c** in a one-pot fashion in <57% yield. The initial attempt was performed using potassium carbonate as a base instead of cesium carbonate, and the reaction worked but a higher temperature was necessary for completion. In addition, an attempt of changing the solvent to dimethylformamide (DMF) was also performed but without any success. Finally, acidic N-Boc removal of compounds **8a**–**b** with HCl gave **9a**–**b** as di-HCl salts.

**Scheme 3:**
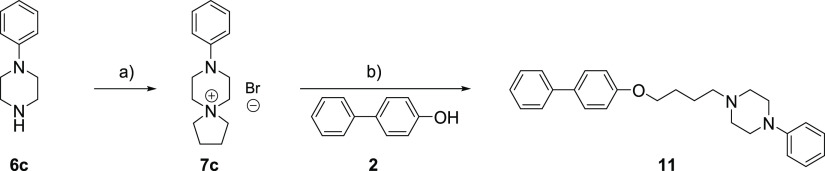
Synthesis of the Phenyl-Substituted Piperazine **11** by Nucleophilic Ring Opening of the Spiro Salt **6c**: (a) K_2_CO_3_, 1,4-Dibromobutane, MeCN, Microwave Heating (115 °C); (b) Cs_2_CO_3_, MeCN, Microwave Heating (170 °C)

For synthesis of the *N*-phenyl-substituted butyl piperazine **11**, the *N*-phenyl piperazine spiro salt **7c** was isolated and reacted as a substrate with 4-biphenol. The synthetic approach for compound **11** is described in Scheme 3. The *N*-phenyl piperazine spiro salt **7c** was synthesized from *N*-phenyl piperazine **6c**, which was reacted with an equivalent amount of 1,4-dibromobutane in acetonitrile and potassium carbonate as a base. The reaction was performed at 115 °C using microwave heating for 15 min, which gave **7c** in 79% yield after isolation. Nucleophilic ring opening of the spiro salt **7c** was performed using acetonitrile as a solvent in excess of cesium carbonate as a base at 170 °C accomplished using microwave heating. After 25 min, the reaction was completed, and compound **11** was isolated in 89% yield.

### Biology: First-Generation Compounds

To evaluate the first-generation compounds, their antiviral activities were analyzed using the rRVFVΔNSs::Katushka virus assay essentially as described previously.^30^ Briefly, A549 cells were infected with rRVFVΔNSs::Katushka (multiplicity of infection (MOI) = 0.1) together with serial dilutions of test compounds and incubated for 16 h. Then, the number of virus-infected cells was quantified by monitoring the expression of Katushka fluorescent proteins in a Trophos plate runner HD (Trophos, Roche Group). To determine the cytotoxic concentration (CC)_50_ values, A549 cells were treated with three-fold serially diluted compounds for 24 h, and the cytotoxicity was measured using a resazurin cell viability assay (Sigma-Aldrich). The results from the antiviral and cytotoxicity assays are summarized in Table 1. Changing the 2-(3-aminopropylamino) ethyl chain in compound **1** to a shorter ethyl (compound **5b**) or propyl amine (compound **5c**) chain resulted in reduction of antiviral activity but improved the cytotoxicity profile. On the other hand, replacing the 2-(3-aminopropylamino) ethyl chain with a hexyl amine chain (compound **5a**) showed a similar antiviral effect to compound **1** but increased the cytotoxicity. Introducing a heterocycle instead of the amino functionality for the ethylamine-substituted compound **5b** to an *N*-piperidine (compound **4d**) or *N*-morpholine (compound **4e**) decreased the antiviral potency but showed an improved cytotoxicity profile. However, the *N*-piperazine-substituted compound **5f** showed similar antiviral potency to the ethylamine-substituted compound **5b**. Increasing the length of the alkyl piperazine chain to butyl (compound **9a**) and pentyl (compound **9b**) showed increased antiviral activity with an increased length of the alkyl ligand, but the propyl (compound **5g**) showed reduced antiviral activity. Butyl and pentyl piperazine compounds **9a** and **9b** showed similar potency with EC_50_ = 12.8 ± 0.2 μM and EC_50_ = 11.7 ± 1.8 μM to the parent compound **1** (12 μM), and butyl piperazine compound **9a** showed a similar cytotoxicity profile (CC_50_ = 74.8 μM ± 1.2) to compound **1** (CC_50_ = 86 μM ± 9); meanwhile, pentyl piperazine compound **9b** showed increased cytotoxicity. Introducing an *N*-methyl in the piperazine moiety (**8c**) decreased the antiviral potency, and for the *N*-phenyl (**11**), the antiviral activity was completely eliminated.

**Table 1:**
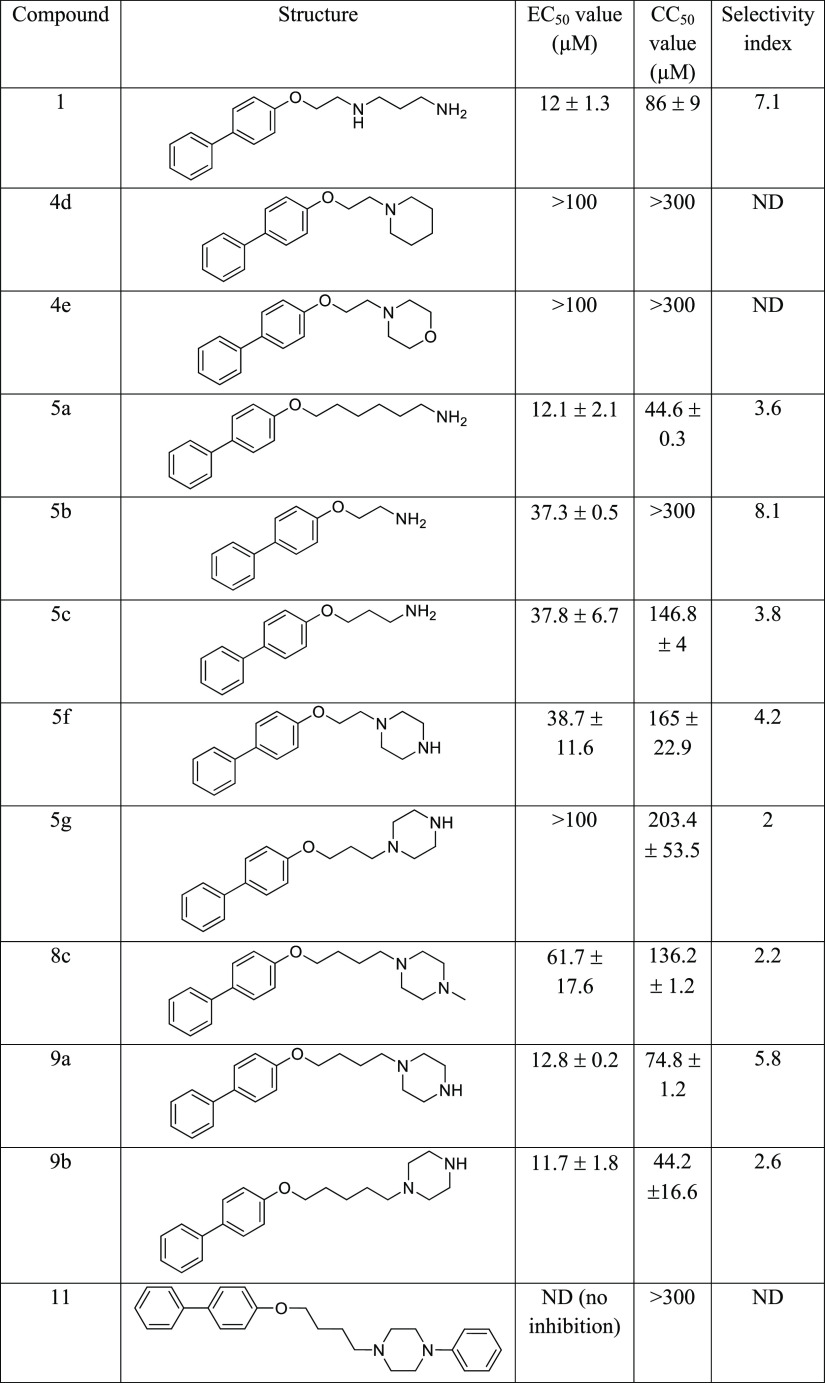
The Antiviral Activity, Cytotoxicity, and Selectivity Index of First-Generation Compounds^a^

a EC_50_, half-maximum effective concentration; CC_50_, cytotoxicity concentration at 50%. Both values are representatives of two independent experiments with two replicates each time. ND, not detectable.

**Figure 2:**
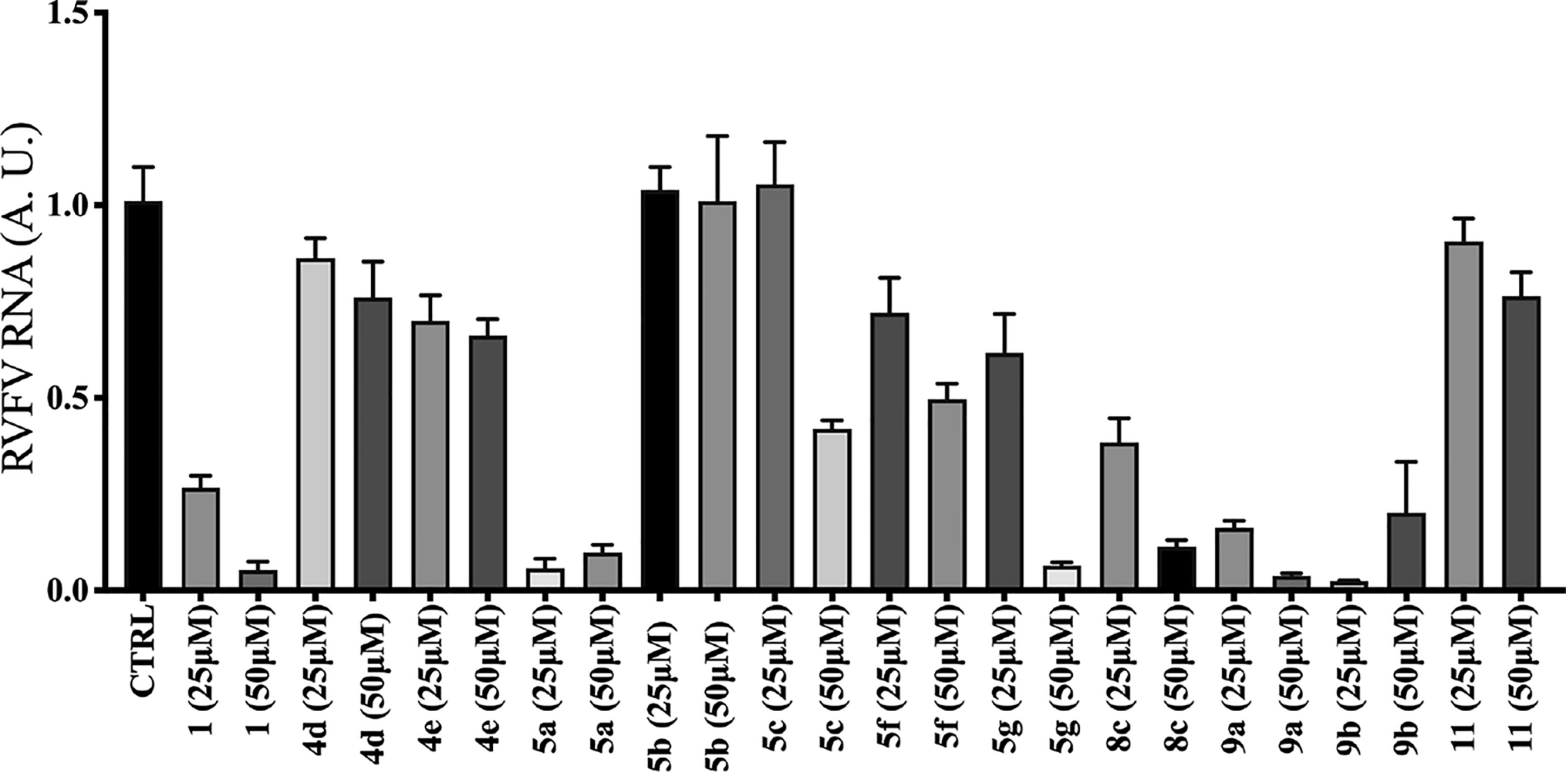
Effect of first-generation compounds on RVFV RNA expression. A549 cells were infected with rRVFVΔNSs::Katushka (MOI = 1) and treated with compounds **1**, **4d**–**e**, **5a**–**c**, **5f**–**g**, **8c**, **9a**–**b**, and **11** at 25 and 50 μM concentrations. The viral load was measured at 16 h post infection using qRT-PCR targeted against the RVFV L segment, which encodes the RVFV RNA-dependent RNA polymerase, and these values were normalized to β-actin mRNA (A.U. = arbitrary units). Each bar represents the mean ± SEM and is representative of two independent experiments with two replicates each time.

The antiviral activity identified from the fluorescence assay was further confirmed with an orthogonal qRT-PCR assay. A549 cells were infected with rRVFVΔNSs::Katushka (MOI = 1) and treated and incubated with the compound for 16 h. Thereafter, viral RNA was purified, and the amount of synthesized viral RNA was quantified by qRT-PCR (Figure 2). As shown in Figure 2, the qRT-PCR results resembled the results obtained from the fluorescence assay. Analogues (**4d**–**e**) were unable to inhibit the RNA expression at the concentrations used in the qRT-PCR assay. Also, the analogues **5b**–**c**, **5f**–**g**, and **8c** were also unable to inhibit the viral RNA expressions. In the fluorescence assay, we observed that analogues **5a** and **9a**–**b** had similar EC_50_ values to compound **1**. These three analogues also had similar patterns to compound **1** regarding inhibition of viral RNA expression. When taking into account the CC_50_ values, butyl piperazine compound **9a** was considered the best among the first-generation analogues with a similar cytotoxicity profile to compound **1**. The *N*-phenyl piperazine analogue **11** did not show any inhibition during the fluorescence assay, and a similar phenomenon was also observed by the qRT-PCR assay.

### Chemistry: Second-Generation Compounds

**Scheme 4:**
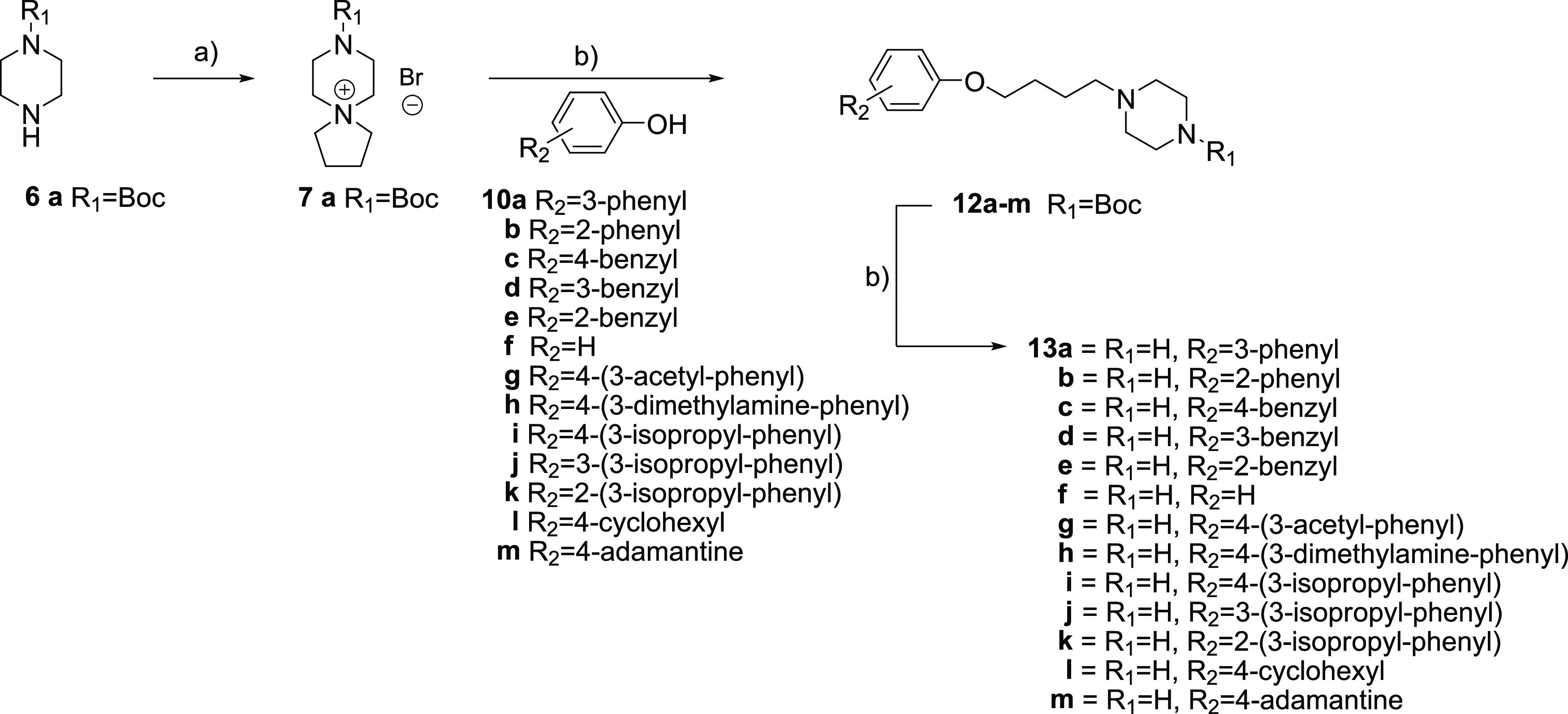
Synthesis of Compounds **13a**–**m** by Nucleophilic Ring Opening of the Spiro Salt **6a**: (a) K_2_CO_3_, 1,4-Dibromobutane, MeCN, Microwave Heating (115 °C); (b) Cs_2_CO_3_, MeCN, Microwave Heating (170 °C)

**Scheme 5:**
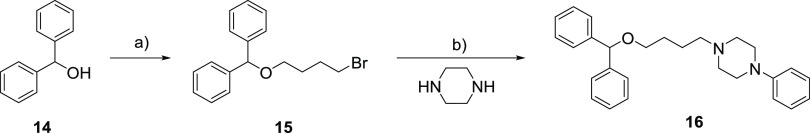
(a) NaH, THF, RT; (b) Cs_2_CO_3_, MeCN, Microwave Heating (115 °C)

Based on the first-generation compounds, we concluded that the butyl piperazine ligand in compound **9a** was the best choice for further modification of the biphenyl part. Compound **9a** with a butyl piperazine ligand showed an equal antiviral activity and toxicity profile to the parent compound **1** with a 2-(3-aminopropylamino)ethyl ligand. For synthesis of the butyl piperazine analogs, the spiro salt **7a** was synthesized and isolated and used as a substrate for further synthesis of the butyl piperazine analogs **12a**–**m** by nucleophilic ring opening of the spiro salt. Acidic removal of the Boc group using HCl gave **13a**–**m** as di-HCl salts in 25–72% yields calculated over two steps. The synthetic approach for compounds **13a**–**m** is described in Scheme 4. For synthesis of compound **16**, the above approach was not successful. Instead, compound **16** was synthesized in two steps described in Scheme 5. First, diphenylmethanol **14** was O-alkylated with 1,4-dibromobutane and sodium hydride as a base to give compound **15** in 22% yield. Second, N-alkylation of piperazine with 1 equiv of compound **15** and excess of cesium carbonate gave compound **16** in 57% yield.

### Biology: Second-Generation Compounds

The butyl piperazine analogues with modification of the biphenyl part showed a wide range of antiviral and cytotoxic activities (Table 2). Changing the biphenyl part to a smaller phenyl group (**13f**) completely removed the antiviral activity. In addition, introducing a more bulky group such as diphenyl methyl (**16**) instead of the biphenyl reduced the antiviral activity significantly. For the investigation of the structural isomers of the biphenyl part, the antiviral activity was similar for both the *para*-position of **9a** (EC_50_ = 12.8 μM ± 0.2) and the *meta*-position of **13a** (EC_50_ = 13.8 ± 5.3 μM) but reduced when the phenyl was located in the *ortho*-position of **13b** (EC_50_ = 63.5 μM ± 11.1). On the contrary, an improved cytotoxicity profile was observed when the phenyl was located in the *meta*-position (CC_50_ = 144.8 ± 5.5 μM). Then, the phenyl was changed to benzyl (**13c**–**e**), and among them, the highest antiviral activity was observed for the benzyl located in the *para*-position of **13c** (EC_50_ = 15.4 ± 4.7 μM) compared to the *ortho*-position of **13d** (EC_50_ = 22.9 ± 5.2) and the *meta*-position of **13e** (EC_50_ = 31.2 ± 15.8). Benzyl-substituted compounds (**13c**–**e**) showed a similar cytotoxicity profile to the *meta*-biphenyl analog **13a**. Introducing a more bulky substituent such as cyclohexyl (**13l**) instead of phenyl (**9a**) in the *para*-position increased the antiviral activity (EC_50_ = 8.8 ± 0.5 μM) but also increased the cytotoxicity. Extreme toxicity was observed when the outer phenyl ring was replaced with adamantine (**13m**). To investigate if addition of an electron-donating, electron-withdrawing, or steric group could affect the antiviral activity, an acetyl (**13g**), dimethylamine (**13h**), or isopropyl (**13j**) substituent was introduced in the *meta*-position of the outer ring of the biphenyl part. For the *meta*-acetyl-substituted compound **13g**, the antiviral activity decreased (EC_50_ = 41.9 ± 27.3 μM), while the *meta*-*N*,*N*-dimethylamine-substituted compound **13h** (EC_50_ = 15.5 ± 0.7 μM) displayed improved antiviral activity but showed high toxicity. Good antiviral activity was detected when the isopropyl substituent was introduced to either the *para*- (**13i**) or *ortho*-position (**13k**). In contrast, addition of an isopropyl group to the biphenyl ring may have enhanced the antiviral activity for **13i**–**k** but diminished the cytotoxicity profile. One interesting observation was that analogues with low EC_50_ values (**13i**–**m**) exhibited high toxicity. This suggested that the observed antiviral activities of these compounds were actually cytotoxic effects.

**Table 2:**
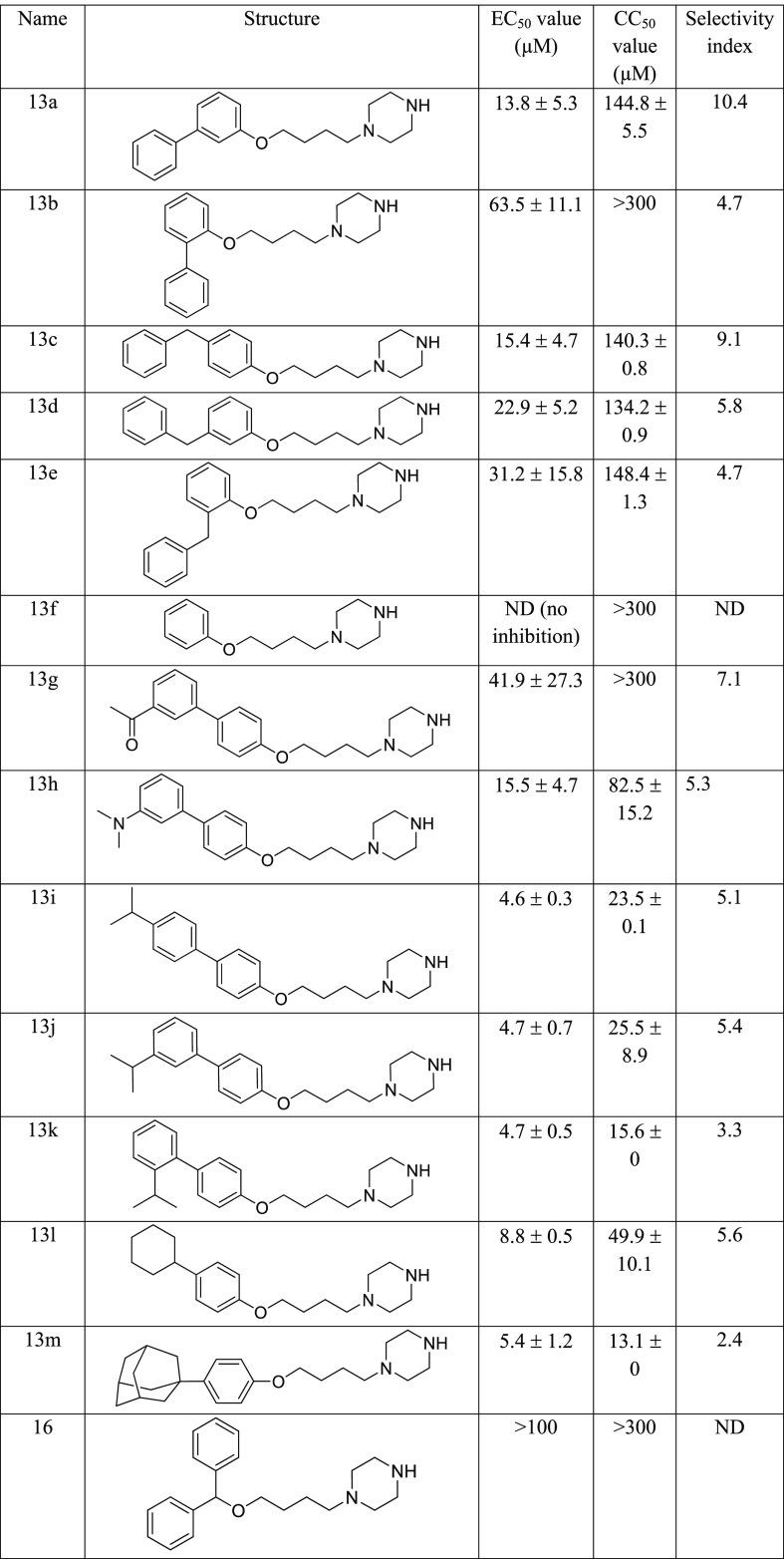
The Antiviral Activity, Cytotoxicity, and Selectivity Index of Second-Generation Compounds^a^

a EC_50_, half-maximum effective concentration; CC_50_, cytotoxicity concentration at 50%. Both values are representatives of two independent experiments with two replicates each time. ND, not detectable.

**Figure 3:**
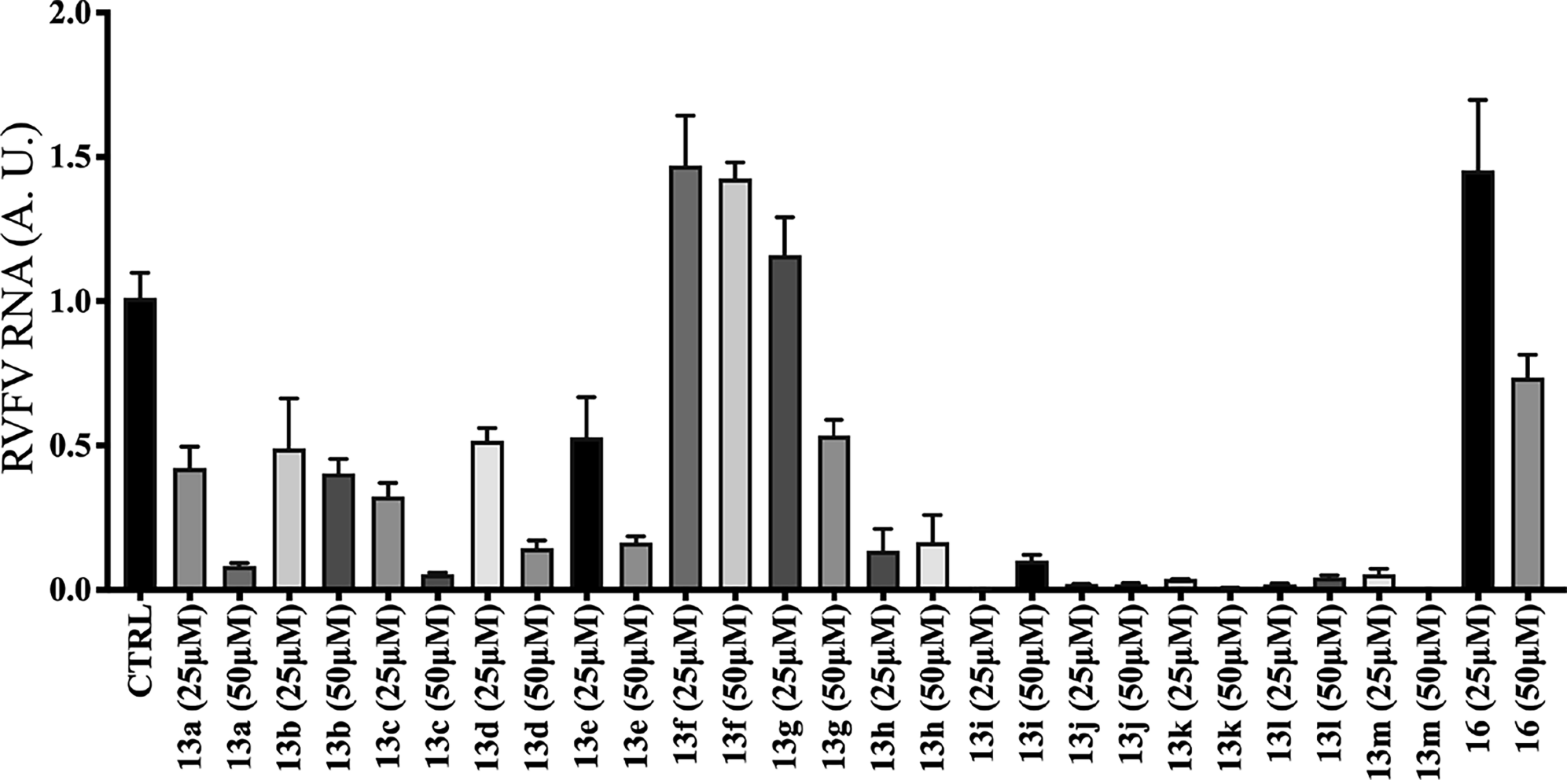
Effects of second-generation compounds on RVFV RNA expression. A549 cells were infected with rRVFVΔNSs::Katushka (MOI = 1) and treated with compounds **13a**–**m** and **16** at 25 and 50 μM concentrations. The viral load was measured at 16 h post infection using qRT-PCR targeted against the RVFV L segment, which encodes the RVFV RNA-dependent RNA polymerase, and these values were normalized to β-actin mRNA (A.U. = arbitrary units). Each bar represents the mean ± SEM and is representative of two independent experiments with two replicates each time.

Similar to the first-generation compounds, the antiviral activity of the second-generation compounds identified from the fluorescence assay was further confirmed by qRT-PCR. Compared to **13a**, compounds (**13b**–**e** and **13 g**–**h**) showed low or no inhibitory effect on RVFV RNA expression. We had similar observation for compounds **13b**–**e** and **13 g**–**h** when we detected the EC_50_ values by the fluorescence assay. In contrast, compounds (**13i**–**m**) having lower EC_50_ values than **13a** in the fluorescence assay also efficiently inhibited the RVFV RNA expression in qRT-PCR. However, as mentioned earlier, these compounds were highly cytotoxic; therefore, the observed antiviral potency was most probably due to their toxic effect on the cells and not because they inhibited the virus itself (Figure 3). Taking into account both antiviral activity and cytotoxicity of all the compounds, **13a** was considered the best to be further explored in a mode-of-action study, although **13a** has similar antiviral activity to compound **1**. However, **13a** had a better cytotoxicity profile than compound **1**.

### Mode-of-Action Studies

**Figure 4:**
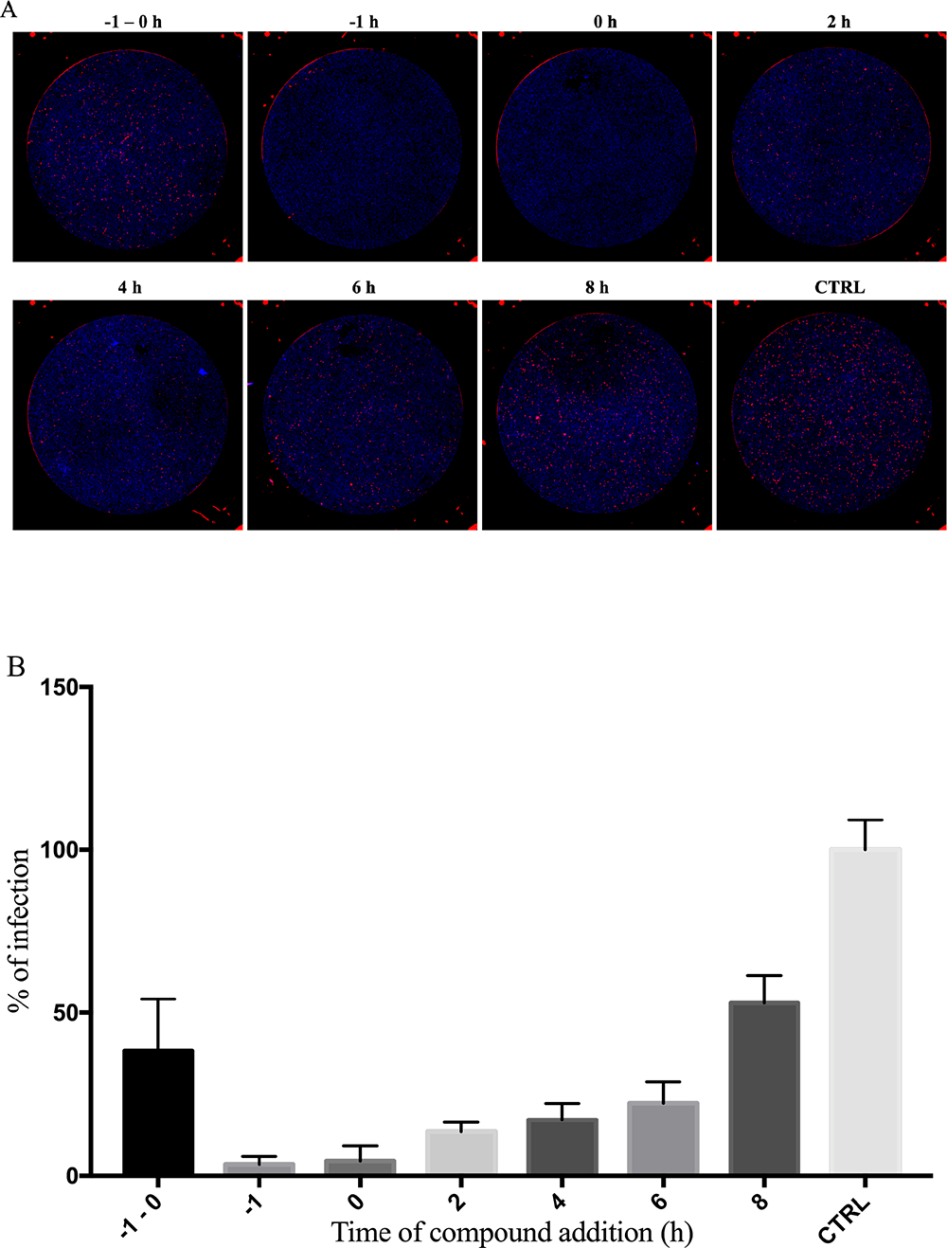
Time-of-addition study. Pre- and post treatment of rRVFVΔNSs::Katushka-infected cells with compound **13a** (50 μM). A549 cells were inoculated with rRVFVΔNSs::Katushka at MOI = 0.1 (time point 0 h). Compound **13a** was added 1 h before infection and then removed at the time of infection (time point −1 to 0 h); the compound was added 1 h prior to infection (time point −1 h), at the same time as infection (time point 0 h), or at the indicated time points post infection and incubated for 13 h, and the percentage of viral infection was determined by a fluorescent cell focus assay. (A) Cells infected with the virus (red) and stained with DAPI (blue). (B) Quantification of the time-of-addition assay in (A). The percentage of infection shown here is relative to the infected control where no antiviral compound was added (CTRL). Each bar represents the mean ± SD and is representative of two independent experiments with at least two replicates each time.

The time-of-addition assay was performed to determine at which stage in the RVFV infection cycle compound **13a** had an effect. Compound **13a** (50 μM) was added at different time points of virus (rRVFVΔNSs::Katushka) infection: preinfection (−1 h before infection), during virus addition (0 h), at early post-entry (2 h post infection (hpi) and 4 hpi), and at late stages of virus infection (6 and 8 hpi). The compound was also added to the cells 1 h before infection, incubated for 1 h, and then removed just before virus addition (−1 to 0 h) as indicated in Figure 4. The experiment was terminated at 13 hpi, and infectivity was assessed by the fluorescence assay, as described previously. As demonstrated in Figure 4, data suggested that the highest antiviral potency was observed when compound **13a** was added in the beginning of virus infection. These results suggested that compound **13a** was active at early stages of the viral replication cycle, most likely during or just after virus entry. The compound **13a** progressively lost its potency when added at late stages of the infection cycle.

It is now well-evident that virion fusion and entry to the host cells are largely regulated by ion channels. Studies have shown that the functionality of ion channels plays a crucial role during entry or post-entry stages of several viruses.^34^ For example, the hepatitis C virus (HCV) requires ion channels for its successful infection cycle.^35,36^ Therefore, ion channels could be new targets to counteract virus infections, with a potentially broad-spectrum antiviral activity useful for future pandemics. Previous antiviral screens of chemical compounds identified several clinically approved ion channel inhibitors as membrane fusion blockers of the HCV.^37−39^ Many of them were closely related to our compound **13a**. Recently, it has been reported that the K^+^ channel regulates the post-entry stages of the Bunyamwera virus (BUNV), a closely related virus to the RVFV, and the authors showed that blocking the K^+^ channel with chemical compounds inhibits BUNV infection.^40,41^ Based on data from our time-of-addition experiments and the above-mentioned facts, we hypothesized that compound **13a** might influence ion channels. Therefore, we performed resting membrane potential experiments to evaluate if compound **13a** has effects on the ion channel (i.e., the K^+^ channel).

**Figure 5:**
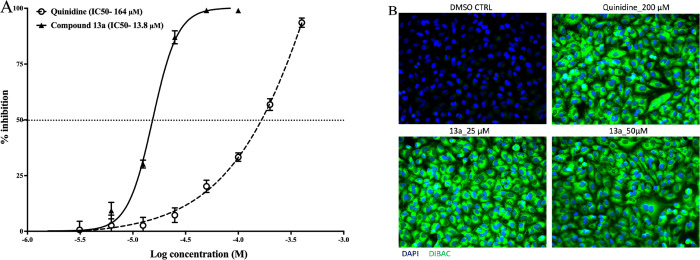
Blocking of the K^+^ channel inhibited RVFV infection. (A) Dose–response curves of inhibition of RVFV infection by quinidine and compound **13a**. A549 cells were infected with rRVFVΔNSs::Katushka (MOI 0.1) in the presence of the compounds. Katushka expression, as a measure of infectivity, was determined 16 h after infection. (B) A549 cells were treated with quinidine (200 μM) or compound **13a** (25 and 50 μM) for 16 h. Cells were fixed and stained with DiBAC_4_(3) (green) and DAPI (blue) and imaged on an Olympus CKX53 fluorescence microscope.

The K^+^ channel plays a vital role to maintain the charge difference across the cell membrane (the resting membrane potential). Due to the changes of the ion channel’s status (either open or closed), the membrane potential can either become more positive (depolarization) or more negative (hyperpolarization). This scenario can be monitored using a membrane potential-sensitive dye, bis(1,3-dibutylbarbituric acid) trimethine oxonol (DiBAC4(3)).^40,42^ An increased DiBAC4(3) fluorescence intensity indicates cell depolarization, while a decreased fluorescence intensity means cellular hyperpolarization. We measured the membrane potential of A549 cells when treated with compound **13a** or quinidine as a positive control to validate the assay. Quinidine is a known K^+^ channel blocker that leads to depolarization. Before performing the membrane potential experiment, we examined the antiviral activity of quinidine and confirmed that it inhibits RVFV infection (EC_50_ value = 146 μM) (Figure 5a). We then performed the membrane potential assay, and A549 cells treated with compound **13a** (25 and 50 μM) exhibited an increased DiBAC4(3) fluorescence intensity (depolarization) similar to quinidine (200 μM), compared to cells that remained untreated, which showed a very low or decreased fluorescence intensity (hyperpolarization) (Figure 5b). This indicated that compound **13a** either blocked directly or had an indirect effect on the K^+^ channel.

## Conclusions

The highly pathogenic RVFV has great health and socioeconomic impacts on endemic countries and could spread to new regions, with potentially devastating consequences. It is crucial to develop better therapeutics to prevent public and animal health threats. In this study, we investigated the SAR of the novel antiviral compound **1**, previously identified from chemical library screening specifically for the RVFV.^30^ In the first-generation compounds, we examined the importance of the (3-aminopropylamino)ethyl chain of compound **1**. Here, we showed that the length of the alkyl amine chain seemed to be important to retain the antiviral activity, but it was also affecting the cytotoxic profile. A general observation was that for compounds with shorter alkyl amine chains (**5b**–**c** and **5f**–**g**), the antiviral efficacy and toxicity against A549 cells decreased, and for compounds with longer alkyl amine chains (**5a** and **9a**–**b**), the antiviral efficacy and toxicity increased. In addition, the presence of a hydrogen bond-donating amine functionality seemed to be necessary for the antiviral activity. When N–H for the ethyl piperazine analog **5f** was replaced with −CH2 (**4d**) or oxygen (**4e**), the antiviral activity was dramatically reduced. Similarly, when the hydrogen bond-donating amine functionality of the butyl piperazine analog **9a** was substituted with *N*-methyl (**8c**) or *N*-phenyl (**11**), the antiviral activity was reduced or completely abolished. The butyl piperazine compound **9a** and the parent compound **1** showed similar antiviral activity and toxicity to A549 cells, but taking into account the CC_50_ values, butyl piperazine compound **9a** was considered the best candidate for further modifications.

In the second-generation compounds, we examined the importance of the biphenyl part of the butyl piperazine compound **9a**. An investigation of the structural isomers by exchanging the *para*-biphenyl (**9a**) with *ortho*-biphenyl (**13a**) or *meta*-biphenyl (**13b**) or substitution of phenyl to benzyl (**13c**–**e**) resulted in no improvement in the antiviral activity. However, when the biphenyl was located in the *ortho*-position (**13a**), the CC_50_ value was improved to almost double compared to the biphenyl in the *para*-position (**9a**). When the biphenyl group was replaced with a phenyl (**13f**) or 1,2-diphenyl methyl (**16**), the antiviral activity was completely removed or significantly reduced. Addition of an isopropyl group to the biphenyl ring induced antiviral activity but showed more toxicity (**13i**–**k**). Therefore, considering both the antiviral activity and the cytotoxicity profile, compound **13a** was the best compound with a selectivity index (SI) of 10. In addition, mode-of-action studies suggested that **13a** could inhibit the post-entry or the early replication phase of the RVFV life cycle, and it affected the K^+^ channel. In the future, it would be interesting to investigate the pharmacokinetics and antiviral efficiency of compound **13a** in an animal model. To conclude, our studies have identified a novel compound with the potential to be further developed as an antiviral drug against the emerging and potentially deadly RVFV infection for which there are no available therapeutics.

## Experimental Section

### Chemistry: General Experimental Procedures

H^1^ and C^13^ NMR spectra were recorded on a Bruker DRX-400 spectrometer (Bruker, Billerica, MA, USA) at 298 K. ^1^H and ^13^C chemical shifts are reported relative to CHCl_3_ (δ_H_ 7.26 ppm) or CDCl_3_ (δ_C_ 77.16 ppm), DMSO-*d*_6_ (δ_H_ 2.50 ppm or δ_C_ 39.52 ppm), and MEOH (δ_H_ 3.33 ppm) or MeOD (δ_C_ 49.0 ppm) as an internal reference. High-resolution mass spectral (HRMS) data were recorded with an Agilent 1290 binary LC system connected to an Agilent 6230 Accurate-Mass TOF LC/MS (ESI+), calibrated with an Agilent G1969-85001 ES-TOF reference mix containing ammonium trifluoroacetate, purine, and hexakis(1*H*,1*H*,3*H*-tetrafluoropropoxy)phosphazine in 90:10 acetonitrile:water. TLC was performed on silica gel 60 F_254_ (Merck Millipore) with detection of UV light. Flash column chromatography [the eluent for flash chromatography is given between brackets in the Experimental Section] was carried out on silica gel (particle size, 60 Å; 230–400 mesh; Sigma-Aldrich). Preparative HPLC was performed using a VP 250/21 Nucleodur C-18, HTEC, 5 μm column (Macherey-Nagel) on a Gilson 333/334 Prep-Scale system with a flow rate of 210 mL/min, detection at 210 nm (Gilson 151), and a CH_3_CN (0.005% HCO_2_H)/H_2_O (0.005% HCO_2_H) eluent system. Compounds **2**, **10c**, **10e**, **10f**, **10l**, and **10b** were purchased from Sigma-Aldrich. Compounds **10d**(32) and **10m**(31) were synthesized according to previously described literature procedures.

### General Synthetic Procedure

#### Procedure A: Synthesis of Compounds **4a**–**g** (R_2_ = Boc) Using the Mitsunobu Reaction, Exemplified for Compound **4a**

Biphenyl-4-ol (86.2 mg, 0.506 mmol), *tert*-butyl 6-hydroxyhexylcarbamate **3a** (100 mg, 0.46 mmol), and PPh_3_ (145 mg, 0.55mmol) were dissolved in 1 mL of THF. To the solution, DIAD (0.11 mL, 0.55 mmol) was added, and the reaction was stirred at rt for 9 days. The resulting mixture was diluted with EtOAc and washed two times with brine. The organic phase was dried with Na_2_SO_4_, filtrated, and concentrated. Purification with flash chromatography [P:E 6:1] over silica gave 172 mg of the Boc-protected compound **4a**, which was taken directly to the next reaction.

#### Procedure B: Synthesis of Compounds **5a**–**c** and Deprotection of the Boc Group Using TFA, Exemplified for Compound **5a**

Boc-protected compound **4a** dissolved in 1.5 mL of DCM and 1.5 mL of TFA was added. After ca. 2.5 h of reaction, it was diluted with water and DCM. NaOH (aq) (2 M) was added until basic pH. The aqueous phase was extracted three times with DCM. Organic phases were combined and washed with brine, dried with anhydrous Na_2_SO_4_ (s), filtrated, and concentrated. Free amine was redissolved in Et_2_O, and 4 M HCl (dioxane) was added until acidic pH. The resulting mixture was concentrated and triturated with Et_2_O three times, which gave **5a** as a HCl salt (52 mg, 0.17 mmol) in 37% yield.

#### Procedure C: Deprotection of the Boc Group Using HCl, Exemplified for Compound **5g**

Boc-protected **4g** (160 mg, 0.404 mmol) was dissolved in DCM (1 mL). HCl (4 M) in dioxane (1.6 mL) was added, and the reaction was stirred at rt for 3 h. The resulting mixture was concentrated and triturated three times with Et_2_O, which gave **5g** (116 mg, 0.315 mmol) as a solid in 60% yield calculated over two steps.

#### Procedure D: One-Pot Synthesis of Compounds **8a**–**c**, Exemplified for Compound **8a**

Boc-piperazine **6a** (100 mg, 0.54 mmol) was dissolved in MeCN (3 mL) in a microwave vessel, and Na_2_CO_3_ (525 mg, 1.61 mmol) was added. To the mixture, 1,4-dibromobutane (67.3 μL, 0.564 mmol) was added, and the reaction was heated in a microwave at 115 °C. After 20 min, 4-biphenol **2** (100 mg, 0.59 mmol) was added, and the reaction was further heated at 170 °C. The resulting mixture was diluted with EtOAc and washed two times with brine. The organic phase was dried with anhydrous Na_2_SO_4_ (s), filtrated, and concentrated. Flash chromatography over silica [2:1 P:E] gave compound **8a** (125 mg, 0.30 mmol) in 56% yield.

#### Procedure E: Spiro Salt Formation, Exemplified for Compound **7a**

Boc-protected piperazine **6a** (500 mg, 2.685 mmol) was dissolved in MeCN (3 mL), and K_2_CO_3_ (1.113 g, 8.054 mmol) and 1,4-dibromobutane (337 μL, 2.819 mmol) were added. The reaction was capped (microwave vessel) and heated using a sand batch at 120 °C for 40 min. The resulting mixture was diluted with CHCl_3_ (product soluble in CHCl_3_) and filtrated to remove K_2_CO_3_ solids. The organic phase was extracted with Milli-Q water twice. The combined water phases were co-concentrated using absolute EtOH. The resulting colorless precipitate was dissolved in CHCl_3_ and dried with anhydrous Na_2_SO_4_ (s), filtrated, and evaporated. After drying under vacuum, it gave **7a** (757 mg, 2.356 mmol) as a colorless solid in 87% yield.

#### Procedure F: Synthesis of Substituted Biphenols Using the Suzuki Coupling Reaction, Exemplified for Compound **10a**

3-Bromophenol (1.26 g, 7.29 mmol), benzeneboronic acid (1,78 g, 14.57 mmol), K_2_CO_3_ (2.52 g, 18.22 mmol), and Pd(PPh_3_)_2_Cl_2_ (26 mg, 0.037 mmol) were mixed with a solution of 20 mL of dioxane and 5 mL of water followed by reflux overnight. The resulting black mixture was diluted with Et_2_O and washed with H_2_O. The water phase was extracted with additional Et_2_O. The combined organic phases were dried with Na_2_SO_4_, filtrated, and concentrated. Flash chromatography over silica [8.5:1 H:E] gave **10a** (0.91 g, 5.35 mmol) as a colorless solid in 73% yield.

#### Procedure G: Nucleophilic Ring Opening of the Spiro Salt, Exemplified for Compound **12a**

The spiro salt **7a** (50 mg, 0.156 mmol) and 3-biphenol **10a** (29.13 mg, 0.171 mmol) were mixed in 0.8 mL of MeCN. Cs_2_CO_3_ (152 mg, 0.467 mmol) was added, and the reaction was microwave heated at 170 °C for 25 min. The resulting mixture was diluted with EtOAc and washed two times with brine. The organic phase was dried with anhydrous Na_2_SO_4_, filtrated, and concentrated. Flash chromatography over silica [1:1 P:E] gave compound **12a** (46 mg, 0.112 mmol) as a sticky oil in 72% yield.

#### *N*1-(2-(Biphenyl-4-yloxy)ethyl)propane-1,3-diamine·2HCl (**1**)

Biphenyl-4-ol (178 mg, 1.05 mmol), *N*,*N*-di-Boc-2-(3-aminopropylamino)ethanol (500 mg, 1.57 mmol), and PPh_3_ (412 mg, 1.57 mmol) were dissolved in 0.4 mL of THF and 0.1 mL of DMF. An increased temperature was needed for complete solvation. To the solution, DIAD (0.3 mL, 1.57 mmol) was added, and the reaction was stirred at 55 °C. After 16 h, additional PPh_3_ (137 mg, mmol) and DIAD (0.1 mL, mmol) were added, and the reaction was further stirred at 70 °C for 24 h. The resulting mixture was diluted with EtOAc and washed two times with brine. The organic phase was dried with Na_2_SO_4_, filtrated, and concentrated. Purification using flash chromatography [E:P 1:7] gave 222 mg of the Boc-protected compound **1** in 45% yield. For removal of the Boc groups, 33 mg of the product was dissolved in 0.4 mL of DCM and 0.5 mL of 4 M HCl (dioxane was added). After 8 h, the resulting mixture was concentrated, and the retained solid was triturated three times with Et_2_O, which gave compound **1** (mg, mmol) as a di-HCl salt in 75% yield. ^1^H NMR (400 MHz, DMSO-*d*_6_): δ 9.34 (broad s, 2H), 8.10 (broad s, 3H) 7.67–7.59 (m, 4H), 7.47–7.41 (m, 2H), 7.35–7.29 (m, 1H), 7.13–7.07 (m, 2H), 4.35 (t, *J* = 5.2 Hz, 2H), 3.39–3.31 (apparent broad s, 2H), 3.14–3.06 (apparent broad s, 2H), 2.97–2.88 (apparent broad s, 2H); ^13^C NMR (100 MHz, DMSO-*d*_6_): δ 157.4, 139.7, 133.3, 128.9, 127.8, 126.9, 126.2, 115.2, 63.4, 46.0, 44.2, 36.1, 23.6; HRMS [M + H]^+^ calculated for C_17_H_23_N_2_O: 271.1810; found, 271.1807.

#### 1-(2-(Biphenyl-4-yloxy)ethyl)piperidine (**4d**)

**4d** was synthesized by procedure A in 37% yield. ^1^H NMR (400 MHz, CDCl_3_): δ 7.57–7.49 (m, 4H), 7.41 (t, *J* = 7.8 Hz, 2H), 7.32–7.27 (m, 1H), 6.98 (d, *J* = 8.7 Hz, 2H), 4.15 (t, *J* = 6.1 Hz, 2H), 2.80 (t, *J* = 6.1 Hz, 2H), 2.57–2.49 (m, 4H), 1.16 (p, *J* = 5.5 Hz, 4H), 1.50–1.41 (m, 2H); ^13^C NMR (100 Hz, CDCl_3_): δ 158.6, 141.1, 134.0, 129.0, 128.4, 127.0, 126.9, 115.1, 66.3, 58.2, 55.3, 26.2, 24.5; HRMS [M + H]^+^ calculated for C_19_H_24_NO: 282.1858; found, 282.1853.

#### 4-(2-(Biphenyl-4-yloxy)ethyl)morpholine (**4e**)

**4e** was synthesized by procedure A in 44% yield. ^1^H NMR (400 MHz, CDCl_3_): δ 7.57–7.50 (m, 4H), 7.42 (t, *J* = 7.8 Hz, 2H), 7.33–7.27 (m, 1H), 7.01–6.96 (m, 2H), 4.16 (t, *J* = 5.75 Hz, 2H), 3.78–3.72 (m, 4H), 2.83 (t, 5.75 Hz, 2H), 2.62–2.58 (m, 4H); ^13^C NMR (100 Hz, CDCl_3_): δ 158.4, 140.9, 134.2, 128.9, 128.3, 126.9, 115.1, 67.1, 66.1, 57.8, 54.3; HRMS [M + H]^+^ calculated for C_18_H_22_NO_2_: 284.1651; found, 284.1648.

#### 6-(Biphenyl-4-yloxy)hexan-1-amine·HCl (**5a**)

**5a** was synthesized by procedures A and B in 37% yield. ^1^H NMR (400 MHz, DMSO-*d*_6_): δ 7.89 (broad s, 2H), 7.63–7.56 (m, 4H), 7.43 (t, *J* = 7.59 Hz, 2H), 7.33–7.27 (m, 1H), 7.04–6.99 (m, 2H), 4.01 (t, *J* = 6.5 Hz, 2H), 2.83–2.72 (m, 2H), 1.78–1.68 (m, 2H), 1.63–1.53 (m, 2H), 1.49–1.34 (m, 2H); ^13^C NMR (100 MHz, DMSO-*d*_6_): δ 158.4, 139.9, 132.5, 128.9, 127.8, 126.7, 126.2, 114.9, 67.3, 38.7, 28.5, 26.9, 25.6, 25.1; HRMS [M + H]^+^ calculated for C_18_H_24_NO: 270.1858; found, 270.1856.

#### 2-(Biphenyl-4-yloxy)ethanamine·HCl (**5b**)

**5b** was synthesized by procedures A and B in 50% yield.

^1^H NMR (400 MHz, CD_3_OD): δ 7.62–7.54 (m, 4H), 7.44–7.37 (m, 2H), 7.32–7.26 (m, 1H), 7.12–7.07 (m, 2H), 4.27 (t, *J* = 5.18 Hz, 2H), 3.39 (t, *J* = 5.2 Hz, 2H); ^13^C NMR (100 MHz, CD_3_OD): δ 159.0, 141.8, 136.1, 129.9, 129.2, 127.9, 127.6, 116.0, 65.4, 40.4; HRMS [M + H]^+^ calculated for C_14_H_16_NO: 214.1232; found, 214.1211.

#### 3-(Biphenyl-4-yloxy)propan-1-amine (**5c**)

**5c** was synthesized by procedures A and B in 42% yield as a free amine. ^1^H NMR (400 MHz, MeOD): δ 7.57–7.50 (m, 4H), 7.41–7.36 (m, 2H), 7.29–7.24 (m, 1H), 7.01–6.96 (m, 2H), 4.09 (t, *J* = 6.12 Hz, 2H), 2.85 (t, *J* = 7.2 Hz, 2H), 1.96 (p, *J* = 6.6 Hz, 2H); ^13^C NMR (100 MHz, MeOD): δ 160.0, 142.1, 135.0, 129.8, 129.0, 127.6, 127.5, 115.9, 67.1, 39.8, 33.3; HRMS [M + H]^+^ calculated for C_15_H_18_NO: 228.1388; found, 228.1381.

#### 1-(2-(Biphenyl-4-yloxy)ethyl)piperazine (**5f**)

**5f** was synthesized by procedures A and B in 41% yield as a free amine. ^1^H NMR (400 MHz, CDCl_3_): δ 7.58–7.49 (m, 4H), 7.45–7.37 (m, 2H), 7.33–7.27 (m, 1H), 7.01 (m, 2H), 4.16 (t, *J* = 5.8 Hz, 2H), 2.93 (t, *J* = 5.0 Hz, 2H), 2.82 (t, *J* = 5.9 Hz, 2H), 2.57 (apparent broad s, 4H), 1.57 (apparent broad s, 1H). ^13^C NMR (100 Hz, CDCl_3_): δ 158.5, 140.9, 134.0, 128.9, 128.3, 126.9, 126.8, 115.0, 66.0, 56.0, 55.2, 46.3; HRMS [M + H]^+^ calculated for C_18_H_23_N_2_O: 283.1810; found, 283.1819.

#### 1-(3-(Biphenyl-4-yloxy)propyl)piperazine (**5g**)

**5g** was synthesized by procedures A and C in 60% yield. ^1^H NMR (400 MHz, DMSO-*d*_6_): δ 12.08 (broad s, 1H), 9.92 (broad s, 2H), 7.65–7.56 (m, 4H), 7.43 (t, *J* = 7.7 Hz, 2H), 7.34–7.27 (m, 1H), 7.07–7.01 (m, 2H), 4.12 (t, *J* = 6.15 Hz, 2H), 3.82–3.64 (m, 2H), 3.56–3.22 (m, 8H) 2.28–2.17 (m, 2H); ^13^C NMR (100 MHz, DMSO-*d*_6_): δ 157.9, 139.8, 132.8, 128.9, 127.8, 126.8, 126.2, 115.0, 65.0, 53.1, 47.8, 39.5, 23.2: HRMS [M + H]^+^ calculated for C_19_H_25_N_2_O: 297.1967; found, 297.1967.

#### 8-(*tert*-Butoxycarbonyl)-8-aza-5-azoniaspiro[4.5]decane Bromide (**7a**)

**7a** was synthesized by procedure E in 87% yield. ^1^H NMR (600 MHz, CDCl_3_): δ 4.09–3.93 (m, 4H), 3.81–3.77 (m, 4H), 3.77–3.65 (m, 4H), 2.39–3.30 (m, 4H), 1.45 (s, 9H); ^13^C NMR (150 MHz, CDCl_3_): δ 153.7, 81.9, 62.7, 59.3, 59.3, 28.4, 21.7; HRMS [M]^+^ calculated for C_13_H_25_N_2_O_2_: 241.1916; found, 241.1915.

#### 8-Phenyl-8-aza-5-azoniaspiro[4.5]decane Bromide (**7d***)*

**7d** was synthesized by procedure E in 89% yield. ^1^H NMR (600 MHz, CDCl_3_): δ 7.31–7.25 (m, 2H), 6.98–6.91 (m, 3H), 4.01–3.95 (m, 4H), 3.93–3.3.87 (m, 4H), 3.53–3.46 (m, 4H), 2.35–3.28 (m, 4H), 1.45 (s, 9H); ^13^C NMR (150 MHz, CDCl_3_): δ 149.1, 129.8, 122.1, 117.0, 62.8, 59.6, 45.6, 21.8; HRMS [M]^+^ calculated for C_14_H_21_N_2_: 217.1705; found, 217.1720.

#### 1-(4-(Biphenyl-4-yloxy)butyl)-4-methylpiperazine (**8c**)

**8c** was synthesized by procedure D in 55% yield and converted to a HCl salt. ^1^H NMR (400 MHz, DMSO-*d*_6_): δ 11.85 (broad s, 2H), 7.65–7.57 (m, 4H), 7.47–7.4 (m, 2H) 7.34–7.28 (m, 1H), 7.06–7.01 (m, 2H), 4.05 (t, *J* = 6.0 Hz, 2H), 3.84–3.3 (m, 8H), 3.28–3.10 (m, 2H), 2.85 (broad s, 3H), 1.95–1.85 (m, 2H); ^13^C NMR (100 MHz, DMSO-*d*_6_): δ 158.1, 139.8, 132.6, 128.9, 127.7, 126.8, 126.2, 114.9, 66.9, 55.4, 49.6, 48.1, 42.1, 25.8, 20.2; HRMS [M + H]^+^ calculated for C_21_H_29_N_2_O: 325.2280; found, 325.2273.

#### 1-(4-(Biphenyl-4-yloxy)butyl)piperazine (**9a**)

**9a** was synthesized by procedures D and C in 51% yield as a di-HCl salt. ^1^H NMR (400 MHz, DMSO-*d*_6_): δ 11.72 (broad s, 1H), 9.62 (broad s, 2H), 7.64–7.56 (m, 4H), 7.43 (t, *J* = 7.5 Hz, 2H), 7.34–7.28 (m, 1H), 7.06–7.01 (m, 2H), 4.05 (t, *J* = 5.9 Hz, 2H), 3.77–3.60 (m, 2H), 3.58–3.36 (m, 4H), 3.33–3.13 (m, 4H), 1.95–1.83 (m, 2H), 1.83–1.74 (m, 2H); ^13^C NMR (100 MHz, DMSO-*d*_6_): δ 158.1, 139.8, 132.6, 128.9, 127.8, 126.7, 126.2, 114.9, 66.9, 55.3, 47.7, 39.7, 25.8, 20.1; HRMS [M + H]^+^ calculated for C_20_H_27_N_2_O: 311.2123; found, 311.2119.

#### 1-(5-(Biphenyl-4-yloxy)pentyl)piperazine (**9b**)

**9b** was synthesized by procedures D and C in 5% yield after purification with HPLC as a free amine. ^1^H NMR (400 MHz, DMSO-*d*_6_): δ 7.63–7.55 (m, 4H), 7.46–7.39 (m, 2H), 7.33–7.27 (m, 1H), 7.03–6.98 (m, 2H), 4.00 (t, *J* = 6.41 Hz, 2H), 2.97–2.91 (m, 4H), 2.49–2.44 (m, 4H), 2.35–2.29 (m, 2H), 1.53–1.37 (m, 4H); ^13^C NMR (100 MHz, DMSO-*d*_6_): δ 158.3, 139.9, 132.4, 128.9, 127.7, 126.7, 126.1, 114.9, 67.5, 57.7, 50.7, 43.7, 28.6, 25.7, 23.4; HRMS [M + H]^+^ calculated for C_21_H_28_N_2_O: 325.2274 ; found 325.2280.

#### Biphenyl-3-ol (**10a**)

**10a** was synthesized by procedure F in 73% yield. ^1^H NMR (400 MHz, CDCl_3_): δ 7.61–7.55 (m, 2H), 7.47–7.41 (m, 2H), 7.40–7.29 (m, 2H), 7.21–7.17 (m, 1H), 7.09–7.07 (m, 1H), 6.84 (ddd, *J*_1_ = 8.0 Hz, *J*_2_ = 2.5 Hz, *J*_3_ = 0.85 Hz, 1H), 5.14 (s, 1H); ^13^C NMR (100 Hz, CDCl_3_): δ 155.9, 143.1, 140.9, 130.1, 128.9, 127.6, 127.3, 119.9, 114.3, 114.2; HRMS [M–H]^−^ calculated for C_12_H_10_O: 169.0653; found, 169.0679.

#### 1-(4′-Hydroxybiphenyl-3-yl)ethanone (**10g**)

**10 g** was synthesized by procedure F in 71% yield. ^1^H NMR (400 MHz, CDCl_3_): δ 8.14 (apparent t, *J* = 1.8 Hz, 1H), 7.91–7.87 (m, 1H), 7.76–7.73 (m, 1H), 7.54–7.47 (m, 3H), 6.95 (apparent d, *J* = 8.7 Hz, 2H), 5.43 (s, 1H), 2.67 (s, 3H); ^13^C NMR (100 Hz, CDCl_3_): δ 198.9, 155.9, 141.5, 137.7, 132.9, 131.6, 129.2, 128.7, 126.9, 126.6, 116.0, 27.0; HRMS [M–H]^−^ calculated for C_14_H_12_O_2_: 211.0759; found, 211.0788.

#### 4′-Isopropylbiphenyl-4-ol (**10i**)

**10i** was synthesized by procedure F in 70% yield. ^1^H NMR (400 MHz, CDCl_3_): δ 7.54–7.47 (m, 4H), 7.32 (apparent d, *J* = 8.3 Hz, 2H), 6.92 (apparent d, *J* = 8.7 Hz, 2H), 5.16 (broad s, 1H), 3.04–2.92 (m, 1H), 1.33 (d, *J* = 6.94 Hz, 6H); ^13^C NMR (100 Hz, CDCl_3_): δ 154.9, 147.6, 138.4, 134.2, 128.4, 126.9, 126.8, 115.8, 33.9, 24.2; HRMS [M–H]^−^ calculated for C_15_H_16_O: 211.1128; found, 211.1127.

#### 3′-Isopropylbiphenyl-4-ol (**10j**)

**10j** was synthesized by procedure F in 82% yield. ^1^H NMR (400 MHz, CDCl_3_): δ 7.49 (apparent d, *J* = 8.6 Hz, 2H), 7.42–7.32 (m, 3H), 7.22–7.16 (m, 1H), 6.91 (apparent d, *J* = 8.6 Hz, 2H), 4.84 (s, 1H), 3.04–2.91 (m, 1H), 1.31 (d, *J* = 6.9 Hz, 6H); ^13^C NMR (100 Hz, CDCl_3_): δ 155.1, 149.5, 140.9, 134.5, 128.8, 128.6, 125.2, 125.0, 124.4, 115.7, 34.4, 24.2; HRMS [M–H]^−^ calculated for C_15_H_16_O: 211.1128; found, 211.1128.

#### 2′-Isopropylbiphenyl-4-ol (**10k**)

**10k** was synthesized by procedure F in 68% yield. ^1^H NMR (400 MHz, CDCl_3_): δ 7.44–7.32 (m, 2H), 7.25–7.16 (m, 4H), 6.90 (apparent d, *J* = 8.62 Hz), 5.12 (broad s, 1H), 3.17–3.04 (m, 1H), 1.19 (d, *J* = 6.9 Hz, 6H); ^13^C NMR (100 Hz, CDCl_3_): δ 154.5, 146.7, 140.7, 134.8, 130.7, 130.3, 127.6, 125.7, 125.4, 115.0, 29.4, 24.4; HRMS [M–H]^−^ calculated for C_15_H_16_O: 211.1128; found, 211.1127.

#### 3′-(Dimethylamino)biphenyl-4-ol (**10h**)

**10 h** was synthesized by procedure F in 29% yield. ^1^H NMR (400 MHz, CDCl_3_): δ 7.48 (apparent d, 8.8 Hz, 2H), 7.3 (t, *J* = 7.7 Hz, 1H), 6.95–6.85 (m, 4H), 6.77–6.72 (m, 1H), 5.34–4-70 (broad s, 1H), 3.01 (s, 6H); ^13^C NMR (100 Hz, CDCl_3_): δ 155.2, 151.1, 142.1, 135.1, 129.6, 128.7, 115.9, 115.6, 111.60, 111.57, 41.0.

#### 1-(4-(Biphenyl-4-yloxy)butyl)-4-phenylpiperazine (**11**)

**11** was synthesized by procedure G in 89% yield: ^1^H NMR (400 MHz, DMSO-*d*_6_): δ 11.0 (broad s, 1H), 7.64–7.57 (m, 4H), 7.46–7.39 (m, 2H), 7.34–7.23 (m, 3H), 7.06–6.98 (m, 4H), 6.89–6.83 (m, 1H), 4.06 (t, *J* = 6.12 Hz, 2H), 3.83–3.77 (m, 2H), 3.59–3.53 (m, 2H), 3.24–3.06 (m, 6H), 1.99–1.87 (m, 2H), 1.85–1.75 (2H); ^13^C NMR (100 Hz, CDCl_3_): δ 158.1, 149.6, 139.8, 132.6, 129.2, 128.9, 127.8, 126.8, 126.2, 120.0, 116.0, 115.0, 66.9, 55.1, 50.6, 45.4, 26.0, 20.1; HRMS [M + H]^+^ calculated for C_26_H_30_N_2_O: 387.2436; found, 387.2436.

#### 1-(4-(Biphenyl-3-yloxy)butyl)piperazine·2HCl (**13a**)

**13a** was synthesized by procedures G and C as a di-HCl salt in 67% yield. ^1^H NMR (400 MHz, DMSO-*d*_6_): δ 11.82 (broad s, 1H), 9.77 (broad s, 2H), 7.72–7.63 (m, 2H), 7.50–7.44 (m, 2H), 7.41–7.35 (m, 2H), 7.25–7.21 (m, 2H), 7.21–7.17 (m, 1H), 6.95 (dd, *J* = 8.2, 2.2 Hz, 1H), 4.09 (d, *J* = 6.0 Hz, 1H), 3.82–3.61 (m, 2H), 3.60–3.336 (m, 4H), 3.36–3.11 (m, 4H), 1.99–1.86 (m, 2H), 1.86–1.75 (m, 2H); ^13^C NMR (100 MHz, DMSO-*d*_6_): δ 159.0, 141.7, 140.1, 130.0, 128.9, 127.6, 126.8, 119.1, 113.7, 112.8, 66.9, 55.3, 47.7, 39.6, 25.9, 20.1; HRMS [M + H]^+^ calculated for C_20_H_27_N_2_O: 311.2123; found, 311.2130.

#### 1-(4-(Biphenyl-2-yloxy)butyl)piperazine·2HCl (**13b**)

**13b** was synthesized by procedures G and C as a di-HCl salt in 50% yield. ^1^H NMR (400 MHz, DMSO-*d*_6_): δ 11.8 (broad s, 1H), 9.73 (apparent broad s, 2H), 7.55–7.42 (m, 4H), 7.37–7.27 (m, 2H), 7.11 (d, *J* = 7.9 Hz. 1H), 7.03 (dt, *J* = 7.5, 0.7 Hz, 1H), 4.00 (t, *J* = 6 Hz, 2H), 3.79–3.33 (m, 6H), 3.28–3.14 (m, 2H), 3.14–3.03 (m, 2H), 1.85–1.75 (m, 2H), 1.75–1.66 (m, 2H); ^13^C NMR (100 MHz, DMSO-*d*_6_): δ 155.3, 138.2, 130.4, 130.0, 129.3, 128.8, 128.1, 126.8, 120.9, 112.7, 67.0, 55.1, 47.5, 39.5, 25.8, 19.9; HRMS [M + H]^+^ calculated for C_20_H_27_N_2_O: 311.2123; found, 311.2122.

#### 1-(4-(4-Benzylphenoxy)butyl)piperazine·2HCl (**13c**)

**13c** was synthesized by procedures G and C as a di-HCl salt in 65% yield. ^1^H NMR (400 MHz, DMSO-*d*_6_): δ 11.62 (broad s, 1H), 9.61 (broad s, 2H), 7.30–7.24 (m, 2H), 7.22–7.09 (m, 5H), 6.88 (m, 2H), 3.95 (t, *J* = 6.0 Hz, 2H), 3.86 (s, 2H), 3.75–3.59 (m, 2H), 3.52–3.36 (m, 4H), 3.28–3.09 (m 4H), 1.89–1.79 (m, 2H), 1.78–1.69 (m, 2H); ^13^C NMR (100 MHz, DMSO-*d*_6_): δ 156.8, 141.8, 133.3, 129.7, 128.5, 128.4, 125.8, 114.4, 66.7, 55.3, 47.7, 40.0, 39.6, 25.8, 20.1; HRMS [M + H]^+^ calculated for C_21_H_29_N_2_O: 325,2280; found, 325.2283.

#### 1-(4-(3-Benzylphenoxy)butyl)piperazine·2HCl (**13d**)

**13d** was synthesized by procedures G and C as a di-HCl salt in 53% yield. ^1^H NMR (400 MHz, DMSO-*d*_6_): δ 11.68 (broad s, 1H), 9.67 (broad s, 2H), 7.35–7.13 (m, 6H), 6.82–6.72 (m, 3H), 3.95 (t, *J* = 5.9 Hz, 2H), 3.76–3.58 (m, 2H), 3.55–3.34 (m, 4H), 3.30–3.06 (m, 4H), 1.91–1.78 (m, 2H), 1.79–1.68 (m, 2H); ^13^C NMR (100 MHz, DMSO-*d*_6_): δ 158.6, 142.9, 141.2, 129.5, 128.7, 128.4, 126.0, 121.1, 115.1, 111.8, 66.6, 55.3, 47.7, 41.1, 39.5, 25.8, 20.1; HRMS [M + H]^+^ calculated for C_21_H_29_N_2_O: 325,2280; found, 325.2272.

#### 1-(4-(2-Benzylphenoxy)butyl)piperazine·2HCl (**13e**)

**13e** was synthesized by procedures G and C as a di-HCl salt in 70% yield. ^1^H NMR (400 MHz, DMSO-*d*_6_): δ 11.81 (broad s, 1H), 9.70 (apparent broad s, 2H), 7.31–7.10 (m, 7H), 6.95 (d, *J* = 8.0 Hz, 1H), 6.86 (t, *J* = 7.4 Hz, 1H), 3.97 (t, *J* = 5.9 Hz, 2H), 3.90 (s, 2H), 3.70–3.58 (m, 2H), 3.54–3.36 (m, 4H), 2.29–3.09 (m, 4H), 1.90–1.80 (m, 2H), 1.80–1.71 (m, 2H); ^13^C NMR (100 MHz, DMSO-*d*_6_): δ 156.1, 140.9, 130.2, 129.2, 128.7, 128.2, 127.6, 125.7, 120.3, 11.6, 66.7, 55.3, 47.6, 39.5, 35.5, 26.0, 20.0; HRMS [M + H]^+^ calculated for C_21_H_29_N_2_O: 325,2280; found, 325.2291.

#### 1-(4-Phenoxybutyl)piperazine·2HCl (**13f**)

**13f** was synthesized by procedures G and C as a di-HCl salt in 63% yield. ^1^H NMR (400 MHz, DMSO-*d*_6_): δ 11.85 (broad s, 1H), 9.83 (broad s, 2H), 7.35–7.22 (m, 2H), 7.00–6.86 (m, 3H), 3.99 (t, *J* = 6 Hz, 2H), 3.75–3.61 (m, 2H), 3.56–3.36 (m, 4H), 3.36–3.15 (m, 4H), 1.93–1.82 (m, 2H), 1.82–1.71 (m, 2H); ^13^C NMR (100 MHz, DMSO-*d*_6_): δ 158.5, 129.5, 120.6, 114.5, 66.6, 55.2, 47.6, 39.5, 25.9, 20.0; HRMS [M + H]^+^ calculated for C_14_H_23_N_2_O: 235.1810; found, 235.1809.

#### 1-(4′-(4-(Piperazin-1-yl)butoxy)biphenyl-3-yl)ethanone·2HCl (**13g**)

**13g** was synthesized by procedures G and C as a di-HCl salt in 67% yield. ^1^H NMR (400 MHz, DMSO-*d*_6_): δ 11.67 (broad s, 1H), 9.59 (broad s, 2H), 8.13 (t, *J* = 1.71 Hz, 1H), 7.91–7.86 (m, 2H), 7.68 (apparent d, *J* = 8.8 Hz, 2H), 7.59 (t, 7.8 Hz, 1H), 7.06 (apparent d, *J* = 8.8 Hz, 2H), 4.06 (t, *J* = 8 Hz, 2H), 3.70–3.60 (m, 2H), 3.55–3.40 (m, 4H), 3.29–3.13 (m, 4H), 2.65 (s, 3H), 1.94–1.75 (m, 4H); ^13^C NMR (100 MHz, DMSO-*d*_6_): δ 198.1, 158.5, 140.3, 137.5, 131.7, 130.8, 129.3, 128.0, 126.5, 125.7, 115.1, 66.9, 55.3, 47.7, 40.2, 26.9, 25.9, 20.1; HRMS [M + H]^+^ calculated for C_22_H_28_N_2_O_2_: 353.2229; found, 353.2228.

#### *N*,*N*-Dimethyl-4′-(4-(piperazin-1-yl)butoxy)biphenyl-3-amine·3HCl (**13h**)

**13h** was synthesized by procedures G and C as a tri-HCl salt in 53% yield. ^1^H NMR (400 MHz, DMSO-*d*_6_): δ 11.82 (broad s, 1H), 9.83 (broad s, 1H), 9.71 (broad s, 1H), 7.86–7.06 (broad m, 4H), 7.63 (d, *J* = 8.63 Hz, 2H), 7.05 (d, *J* = 8.63 Hz, 2H), 4.05 (t, *J* = 6.19 Hz, 2H), 3.72–3.67 (m, 2H), 3.55–3.42 (m, 4H), 3.31–3.26 (m, 2H), 3.25–3.20 (m, 2H), 1.93–185 (m, 2H), 1.83–1.77 (m, 2H); ^13^C NMR (100 MHz, DMSO-*d*_6_): δ 158.5, 130.0, 128.0, 115.0, 66.9, 55.2, 47.7, 40.1, 25.8, 20.0; HRMS [M + H]^+^ calculated for C_22_H_31_N_3_O: 354.2545; found, 354.2550.

#### 1-(4-(4′-Isopropylbiphenyl-4-yloxy)butyl)piperazine·2HCl (**13i**)

**13i** was synthesized by procedures G and C as a di-HCl salt in 72% yield. ^1^H NMR (400 MHz, DMSO-*d*_6_): δ 11.88 (broad s, 1H), 9.84 (broad s, 2H), 7.60–7.49 (m, 4H), 7.30 (d, *J* = 8.6 Hz, 2H), 7.02 (d, *J* = 8.6 Hz, 2H), 4.04 (t, *J* = 6 Hz, 2H), 3.74–3.25 (m, 8H), 3.25–3.16 (m, 2H), 2.91 (m, 1H), 1.96–1.84 (m, 2H), 1.84–1.75 (m, 2H); ^13^C NMR (100 MHz, DMSO-*d*_6_): δ 157.5, 145.9, 140.3, 133.8, 129.8, 127.5, 125.5, 125.4, 114.1, 66.8, 55.2, 47.7, 40.1, 28.9, 25.9, 24.1, 20.1; HRMS [M + H]^+^ calculated for C_23_H_32_N_2_O: 353.2593; found, 353.2595.

#### 1-(4-(3′-Isopropylbiphenyl-4-yloxy)butyl)piperazine·2HCl (**13j**)

**13j** was synthesized by procedures G and C as a di-HCl salt in 45% yield. ^1^H NMR (400 MHz, DMSO-*d*_6_): δ 11.82 (broad s, 1H), 9.78 (broad s, 2H), 7.59 (apparent d, *J* = 8.68 Hz, 2H), 7.47–7.43 (m, 1H), 7.42–7.33 (m, 1H), 7.34 (t, *J* = 7.58 Hz, 1H), 7.20–7.16 (m, 1H), 7.02 (apparent d, *J* = 8.68 Hz, 2H), 4.04 (t, *J* = 6.13 Hz, 2H), 3.75–3.63 (m, 2H), 3.56–3.40 (m, 4H), 3.33–3.09 (m, 4H), 2.94 (m, 1H), 1.94–1.81 (m, 2H), 1.84–1.74 (m, 2H), 1.24 (d, *J* = 6.92 Hz, 6H); ^13^C NMR (100 MHz, DMSO-*d*_6_): δ 158.1, 149.1, 139.9, 133.0, 128.9, 127.9, 124.7, 124.3, 123.8, 114.9, 66.9, 55.3, 47.7, 39.8, 33.6, 25.9, 24.0, 20.1; HRMS [M + H]^+^ calculated for C_23_H_32_N_2_O: 353.2593; found, 353.2595.

#### 1-(4-(2′-Isopropylbiphenyl-4-yloxy)butyl)piperazine·2HCl (**13k**)

**13k** was synthesized by procedures G and C as a di-HCl salt in 50% yield. ^1^H NMR (400 MHz, DMSO-*d*_6_): δ 11.77 (broad s, 1H), 9.74 (broad s, 2H), 7.42–7.37 (m, 1H), 7.35–7.29 (m, 1H), 7.22–7.15 (m, 1H), 7.22–7.15 (m, 3H), 7.11–7.06 (m, 1H), 7.02–76.96 (m, 2H), 4.04 (t, *J* = 5.8 Hz, 2H), 3.74–3.65 (m, 2H), 3.33–3.16 (m, 4H), 2.99 (m, *J* = 7 Hz, 1H), 1.94–1.85 (m, 2H), 1.85–1.76 (m, 2H), 1.11 (d, *J* = 7 Hz, 6H); ^13^C NMR (100 MHz, DMSO-*d*_6_): δ 157.9, 146.9, 137.4, 132.6, 127.5, 126.8, 126.1, 114.9, 66.8, 55.2, 47.6, 40.2, 33.0, 25.8, 23.9, 20.0; HRMS [M + H]^+^ calculated for C_23_H_32_N_2_O: 353.2593; found, 353.2592.

#### 1-(4-(4-Cyclohexylphenoxy)butyl)piperazine·2HCl (**13l**)

**13l** was synthesized by procedures G and C as a di-HCl salt in 25% yield. ^1^H NMR (400 MHz, DMSO-*d*_6_): δ 11.64 (broad s, 1H), 9.64 (broad s, 1H), 7.11 (d, *J* = 8.64 Hz, 2H), 6.83 (d, *J* = 8.64 Hz, 2H), 3.95 (t, *J* = 6.03 Hz, 2H), 3.73–3.64 (m, 2H), 3.54–3.38 (m, 4H), 3.30–3.11 (m, 4H), 2.46–2.38 (m, 1H), 1.91–1.64 (m, 8H), 1.41–1.14 (m, 4H); ^13^C NMR (100 MHz, DMSO-*d*_6_): δ 156.7, 139.8, 127.5, 114.3, 66.7, 55.3, 47.7, 42.9, 40.1, 34.3, 26.4, 25.9, 25.6, 20.1; HRMS [M + H]^+^ calculated for C_20_H_32_N_2_O: 317.2593; found, 317.2599.

#### 1-(4-(4-Adamantylphenoxy)butyl)piperazine·2HCl (**13m**)

**13m** was synthesized by procedures G and C as a di-HCl salt in 59% yield. ^1^H NMR (400 MHz, DMSO-*d*_6_): δ 11.83 (s, 1H), 9.85 (m, 2H), 7.24 (d, *J* = 8.88 Hz, 2H), 6.86 (d, *J* = 8.88 Hz, 2H), 3.95 (t, *J* = 6.15 Hz, 2H), 3.72–3.62 (m, 2H) 3.54–3.39 (m, 4H), 3.335–3.12 (m, 4H), 2.03 (broad s, 3H), 1.92–1.64 (m, 14H); ^13^C NMR (100 MHz, DMSO-*d*_6_): δ 156.9, 154.9, 143.8, 125.9, 114.0, 79.7, 67.7, 58.4, 53.1, 43.5, 37.0, 35.7, 29.1, 28.6, 27.5, 23.5; HRMS [M + H]^+^ calculated for C_24_H_36_N_2_O: 369.2906; found, 369.2903.

#### 1-(4-(Benzhydryloxy)butyl)-4-phenylpiperazine (**16**)

Diphenyl methanol (500 mg, 2.71 mmol) was dissolved in 15 mL of dry THF, and NaH (119.4 mg, 2.98 mmol) was added under stirring at rt. After 15 min, 1,4-dibromobutane (1.347 mg, 6.24 mmol) was added, and the reaction was stirred over the weekend at a temperature of 65 °C. The resulting mixture was diluted with EtOAc and washed two times with brine. The organic phase was dried with anhydrous Na_2_SO_4_, filtrated, and concentrated. Purification with flash chromatography [1:40, H:E] gave 190 mg of ((4-bromobutoxy)methylene)dibenzene **15** in 22% yield. Compound **15** (50 mg, 0.156 mmol), piperazine (134.9 mg, 1.566 mmol), and Cs_2_CO_3_ (325.8 mg, 0.469 mmol) were mixed in acetonitrile (0.4 mL). The reaction was done using microwave heating at 115 °C for 20 min. The resulting mixture was diluted with EtOAc and washed two times with brine. The organic phase was dried with anhydrous Na_2_SO_4_, filtrated, and concentrated. Purification by flash chromatography gave compound **16** (29 mg, 0.089 mmol) in 57% yield. ^1^H NMR (400 MHz, DMSO-*d*_6_): δ 7.37–7.27 (m, 8H), 7.25–7.19 (m, 2H), 5.32 (s, 1H), 3.50–3.42 (m, 3H), 2.93 (apparent t, *J* = 4.8 Hz, 4H), 2.50–2.38 (m, 4H), 2.36–2.30 (m, 2H), 1.70–1.55 (m, 4H); ^13^C NMR (100 MHz, DMSO-*d*_6_): δ 142.7, 128.5, 127.4, 127.0, 83.7, 69.0, 59.0, 53.9, 45.8, 28.0, 23.6; HRMS [M + H]^+^ calculated for C_21_H_28_N_2_O: 325.2280; found, 325.2282.

### Cells and the Virus

Human lung adenocarcinoma basal epithelial cells, A549, were cultured in a cell culture medium (Dulbecco’s modified Eagle’s medium [DMEM], Sigma-Aldrich, St. Louis, MO) containing 0.75 g NaHCO_3_/L, 20 mM HEPES (4-[2-hydroxyethyl]-1-piperazineethanesulfonic acid) (EuroClone, Milan, Italy), penicillin G (100 IU/mL) and streptomycin sulfate (100 μg/mL) combined (1× PEST, Gibco, Carlsbad, CA), and 5% fetal bovine serum (FBS, Gibco) at 37 °C. For virus infection, a cell maintenance medium was used containing the same components, except at a lower FBS concentration (2%). The replication-competent recombinant RVFV expressing the far-red fluorescent protein Katushka instead of the NSs protein (rRVFVΔNSs::Katushka) was used for the whole study.^30^

### Effective Concentration 50 (EC_50_) Assay

To determine the EC_50_ value of the compounds, the fluorescence intensity of individual infectious cell foci was quantified in a dose-dependent manner for all compounds, described previously.^30^ Briefly, approximately 10,000 A549 cells/well were seeded in 96-well black-wall plates with a transparent bottom on the day before infection. Just before infection, compounds were serially diluted in three-fold steps from 100 to 0.045 μM and mixed with 1000 plaque forming units of the rRVFVΔNSs::Katushka virus in a total volume of 100 μL of DMEM containing 2% FBS, with multiplicity of infection (MOI) = 0.1. The growth medium was removed, 100 μL of the virus and the compound mixture was added to the cells, and the plate was incubated for 16 h at 37 °C in 5% CO_2_. Later, the medium was removed, and cells were fixed for 1 h with 3% paraformaldehyde (PFA); then, the cellular nuclei were stained with 0.1% DAPI for 15 min. The wells were washed with PBS, and the number of infected cells/well was counted by a Trophos plate runner HD (Trophos, Roche Group) following the expression of the Katushka protein by the virus. Simultaneously, the total number of cells/well was also counted following the DAPI staining. GraphPad Prism software version 9.2.0 (GraphPad Software, La Jolla, CA, USA) was used to calculate the EC_50_ value with nonlinear regression analysis with a variable slope. All laboratory work with the rRVFVΔNSs::Katushka virus was performed under biosafety level 2 conditions as approved by the Swedish Work Environment Authority.

### Cellular Toxicity Assay

The resazurin cell viability assay (Sigma-Aldrich) was used to analyze the cellular toxicity of synthesized analogues, described previously. This assay measures the metabolic activity of living cells and is based on the oxidoreduction of the nontoxic indicator blue dye resazurin. Viable cells with active metabolism can reduce resazurin into resorufin, which is pink and fluorescent. Briefly, A549 cells (approximately 10,000/well) were seeded in a black-wall transparent-bottom 96-well plate and incubated at 37 °C in 5% CO_2_ overnight. Cells were then treated with compound concentrations starting from 300 μM with 2-fold serial dilutions down to 2.34 μM and incubated at 37 °C in 5% CO_2_ for 24 h. To analyze the cell survival/toxicity, 10 μL (40 μM final concentration) of resazurin was added per well and incubated for 3–4 h at 37 °C in a 5% CO_2_ incubator, and the resorufin fluorescence intensity was measured by a Trophos plate runner HD (Trophos, Roche Group). The CC_50_ value was then calculated with GraphPad Prism software version 9.2.0 (GraphPad Software, La Jolla, CA, USA) following the nonlinear regression analysis with a variable slope.

### Viral RNA Extraction and qRT-PCR

Cell seeding, virus infection with rRVFVΔNSs::Katushka (MOI = 1.0), and compound addition were carried out in the same way as previously described.^33^ Briefly, A549 cells (approximately 10,000/well) were seeded in transparent 24-well plates and incubated at 37 °C in 5% CO_2_ overnight. Cells were then infected with rRVFVΔNSs::Katushka (MOI = 1.0) together with the compound (50 and 25 μM) and incubated at 37 °C in 5% CO_2_ for 16 h. Then, the virus inoculum was discarded, cells were washed with PBS and lysed with proteinase K, and the total cellular RNA was extracted. Extraction of viral RNA and quantification of the viral load were performed as previously described.^33^

### Time-of-Addition Assay

The fluorescent cell focus assay was used for the time-of-addition assay, as previously described.^33^ In short, A549 cells were infected at an MOI of 0.1, and 50 μM compound **13a** was added prior to infection (−1 h), at the time of infection (0 h), and at 2, 4, 6, and 8 h after infection. An additional experiment was to treat cells with 50 μM of the compound 1 h before infection (−1 h) and then remove the cell medium, containing the compound, at the time of infection (−1 to 0 h). The infection was assayed by the fluorescent cell focus assay at 13 h post infection. The cellular nucleoli were stained with 0.1% DAPI and counted as described in the previous section.

### Resting Membrane Potential Assay

Before performing the resting membrane potential assay, we performed the dose–response activity of quinidine and compound **13a**, similar to the effective concentration assay. The only exception was that both compounds were serially diluted in two-fold steps, from 400 to 3.12 μM for quinidine and from 100 to 1.56 μM for compound **13a**. The resting membrane potential assay was performed as previously described.^40^ Briefly, A549 cells were treated with 20 μM DiBAC_4_(3) (Sigma) for 20 min at 37 °C in the dark. After labeling, cells were washed and treated with either quinidine (200 μm) as a positive control or compound **13a** (25 and 50 μM) for 16 h. Labeled cells were also left untreated as the DiBAC_4_(3) control. Wide-field images were taken with an Olympus CKX53 fluorescence microscope.

## Supporting Information Available

Nuclear magnetic resonance (NMR) spectra and high-performance liquid chromatography (HPLC) analysis of the compounds; purity of the compounds used in the experiments analyzed by NMR and HPLC (PDF)
