# Supplementary material for: Structural Modifications and Biological Evaluations of Rift Valley Fever Virus Inhibitors Identified from Chemical Library Screening
Source: ACS Omega. 2022 Feb 16;7(8):6854–68. doi: 10.1021/acsomega.1c06513 (PMC8892858; doi:10.1021/acsomega.1c06513)
Supplement: Supplementary file 1 — ao1c06513_si_001.pdf [file ao1c06513_si_001.pdf]

Supporting information

Structural modifications and biological evaluations of Rift Valley fever virus inhibitors identified from chemical library screening.

Koushikul Islam[1#], Marcus Carlsson[2#], Per-Anders Enquist[2], Weixing Qian[2], Marko Marttila[1], Mårten Strand[1], Clas Ahlm[1], Magnus Evander[1*].

[1]Department of Clinical Microbiology, Umeå University, Umeå, Sweden. [2]Department of Chemistry, Umeå University, Umeå, Sweden.

[#] Authors contributed equally

*Corresponding author

Supporting information - Methods

NMR

Purity in molar values

Quantitative proton NMR: Integral values were normalized to 1H for target molecule and impurities. The ratio between target molecule value and total value (calculated sum of target molecule and impurities values) was calculated and multiplied by 100 to receive the purity in %.

Purity in mass

Quantitative proton NMR: Integral values were normalized to 1H for target molecule and impurities. Each value was multiplied with the corresponding molecular mass. The ratio between target molecule value and total value (calculated sum of target molecule and impurities value) was calculated and multiplied by 100 to receive the purity in %.

HPLC

For HPLC analysis, samples were dissolved in 50% of 5 mM ammonium formate buffer pH=3.9 and 50% of acetonitrile with 0.01% formic acid mixture. Quantitative analysis was performed using ZIC – HILIC, 5µm, 200Å, 150x4.6 column on a SHIMADZU LC-30AD system with 50% of 5 mM ammonium formate buffer pH=3.9 and 50% of acetonitrile with 0.01% formic acid mobile phase and a flow rate of 1.0 mL/min, SPD-M20A detector at 260 nm. Software was Labsolutions Version 5.51.

Results

Figure **S1**. ^{1}H-NMR (400 MHz, CDCl3) spectrum of compound **1**.

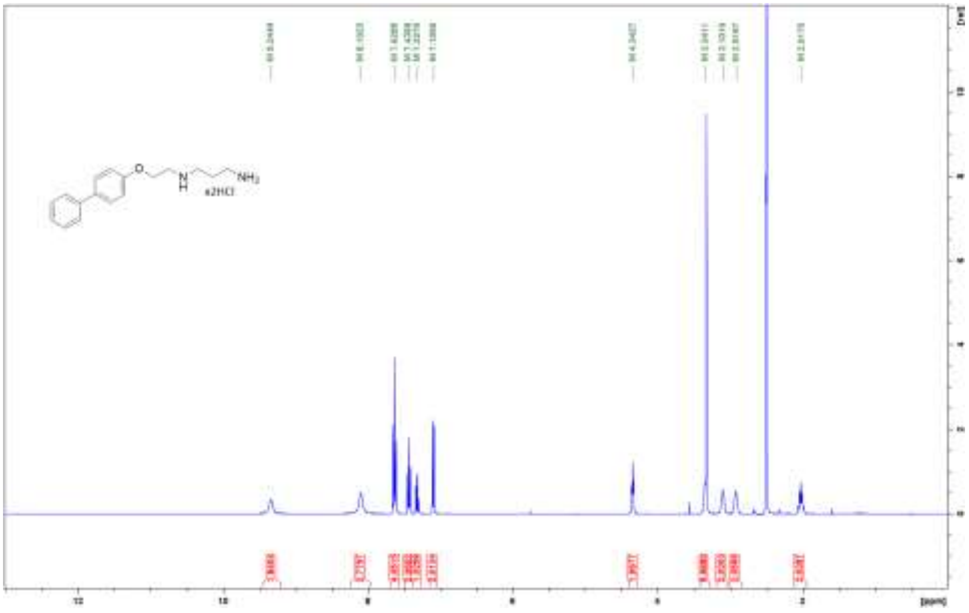

Figure **S2**. ^{13}C-NMR (100 MHz, CDCl3) spectrum of compound **1**.

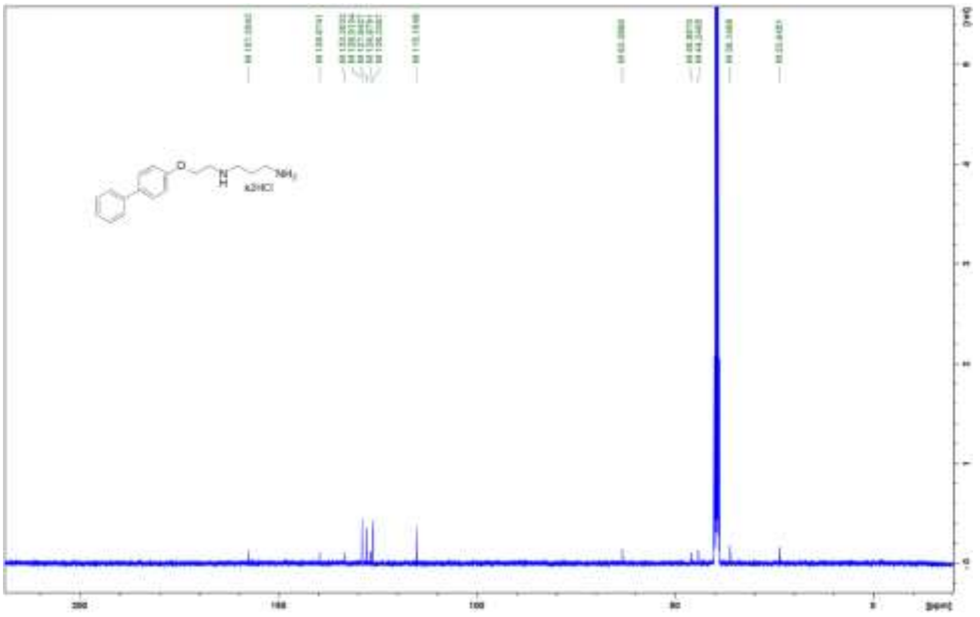

Figure **S3**. ^{1}H-NMR (400 MHz, CDCl3) spectrum of compound **4d**.

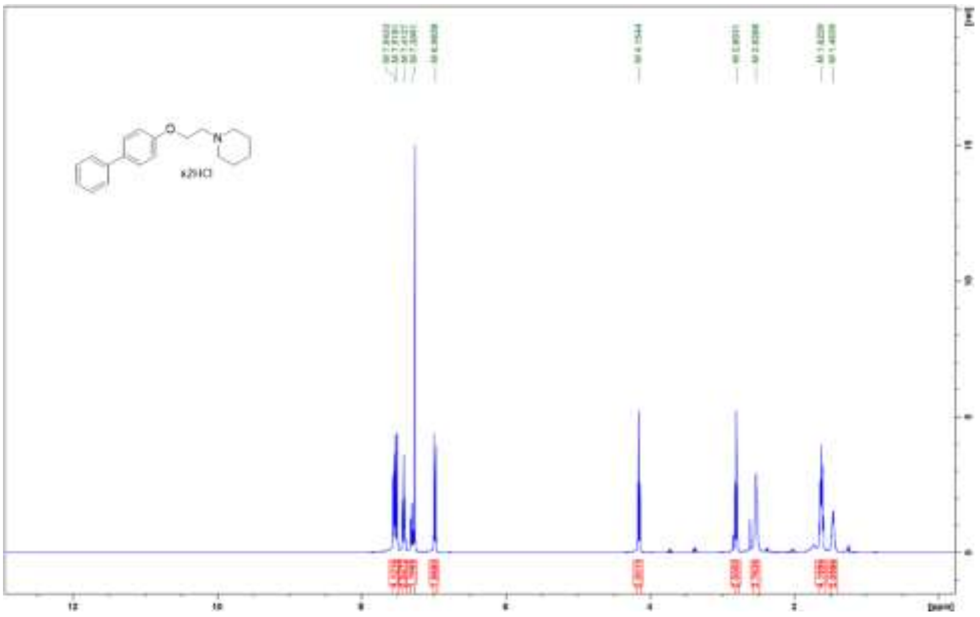

Figure **S4**. ^{13}C-NMR (100 MHz, CDCl3) spectrum of compound **4d**.

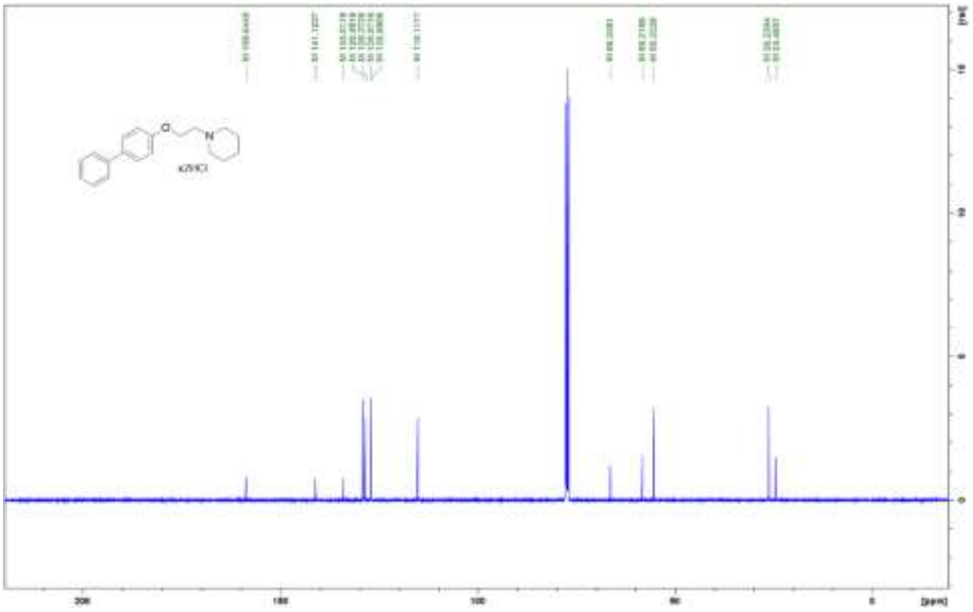

Figure **S5**. ¹H-NMR (400 MHz, CDCl3) spectrum of compound **4e**.

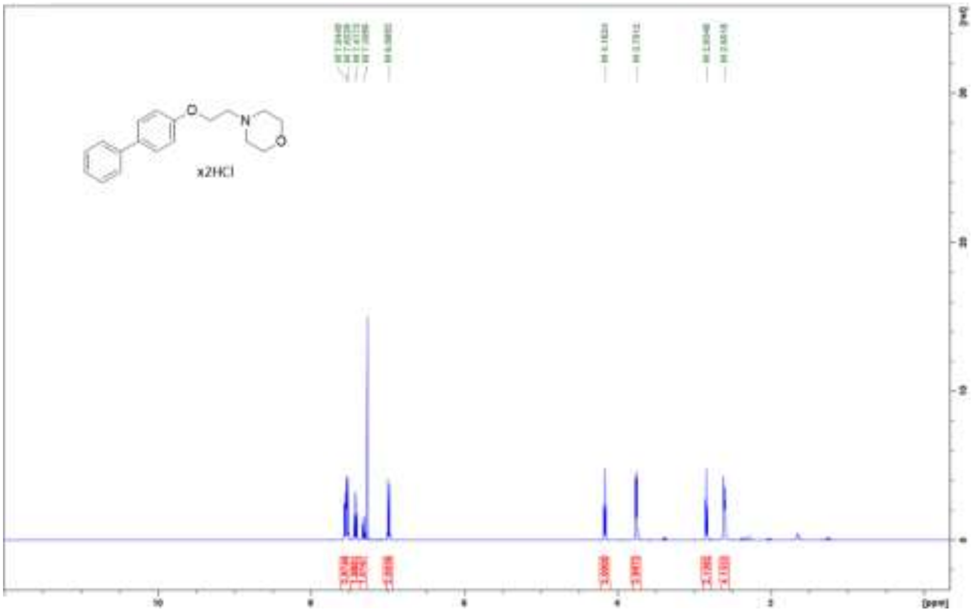

Figure **S6**. ¹³C-NMR (100 MHz, CDCl3) spectrum of compound **4e**.

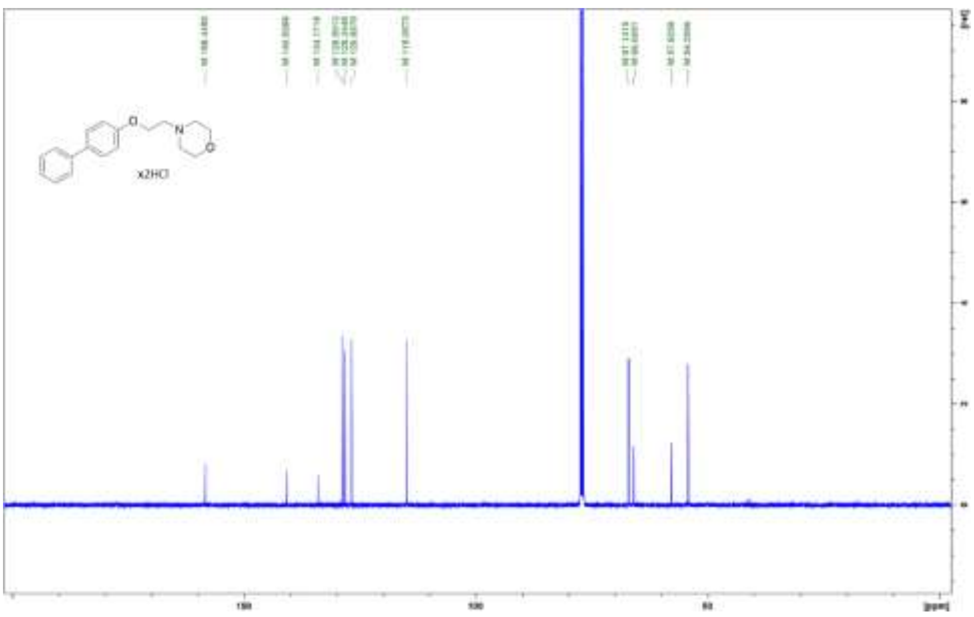

Figure **S7**. ^{1}H-NMR (400 MHz, CDCl3) spectrum of compound **5a**.

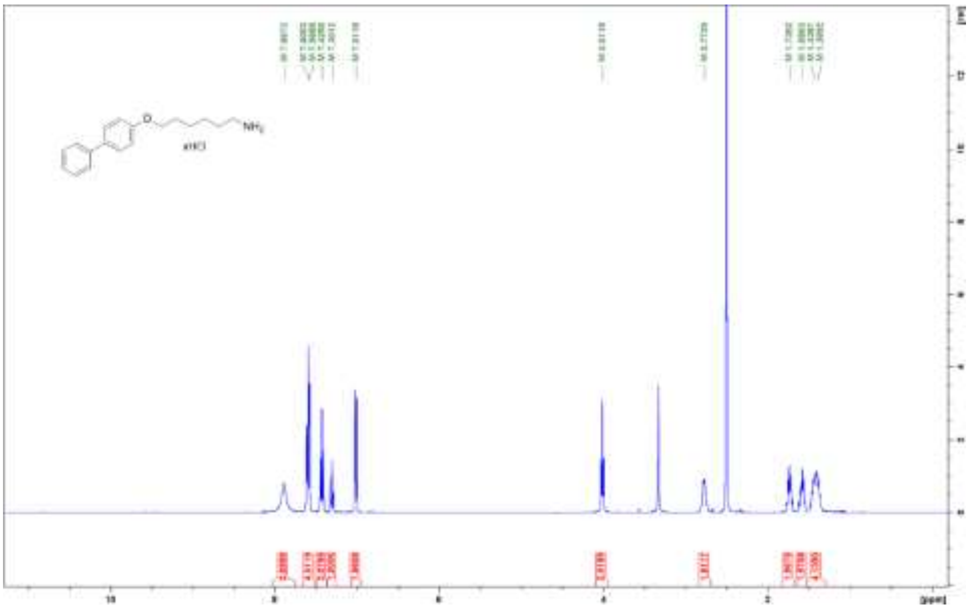

Figure **S8**. ^{13}C-NMR (100 MHz, CDCl3) spectrum of compound **5a**.

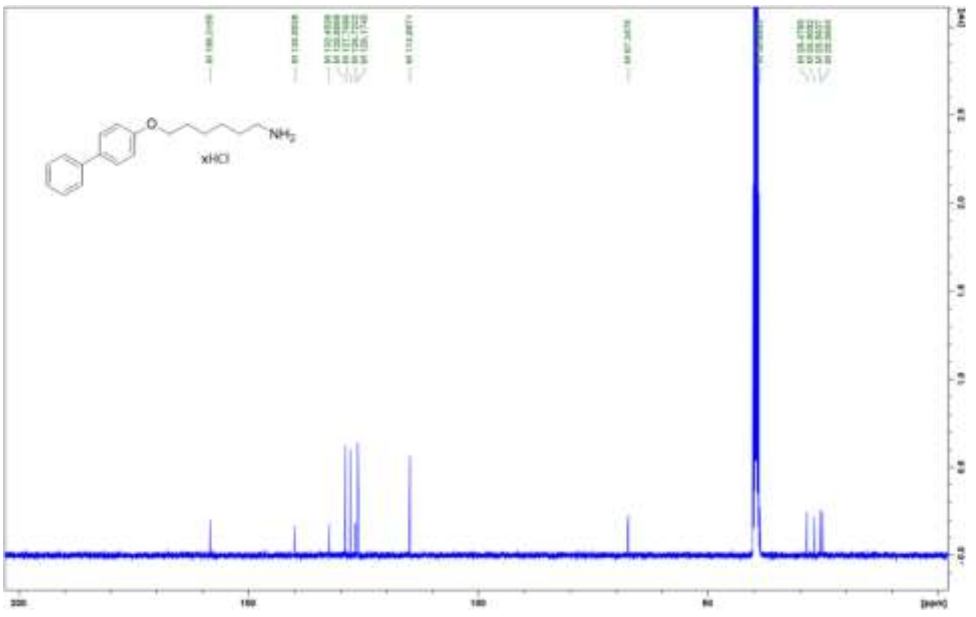

Figure **S9**. ^{1}H-NMR (400 MHz, CDCl3) spectrum of compound **5b**.

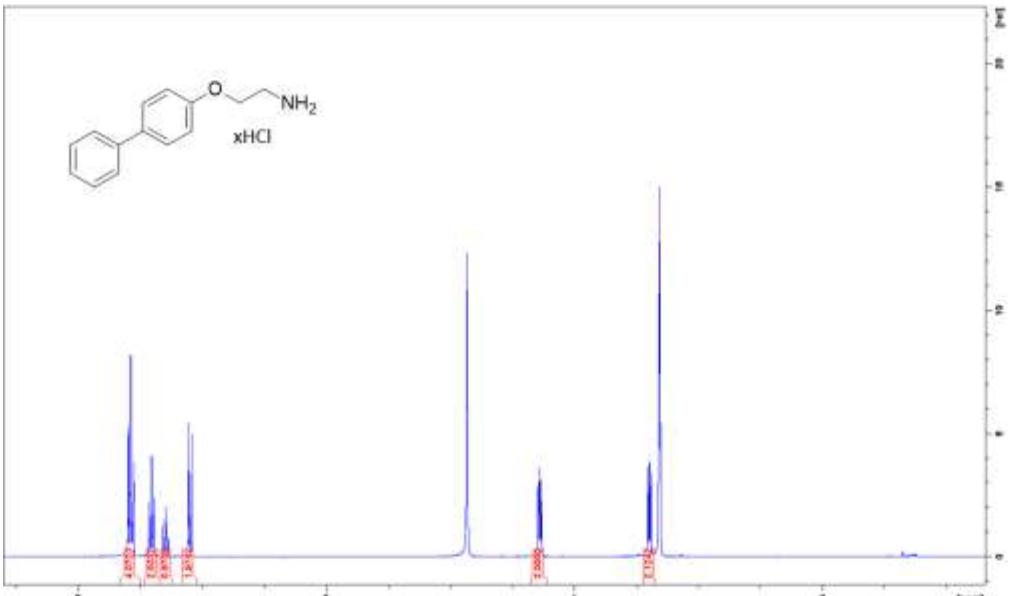

Figure **S10**. ^{13}C-NMR (100 MHz, CDCl3) spectrum of compound **5b**.

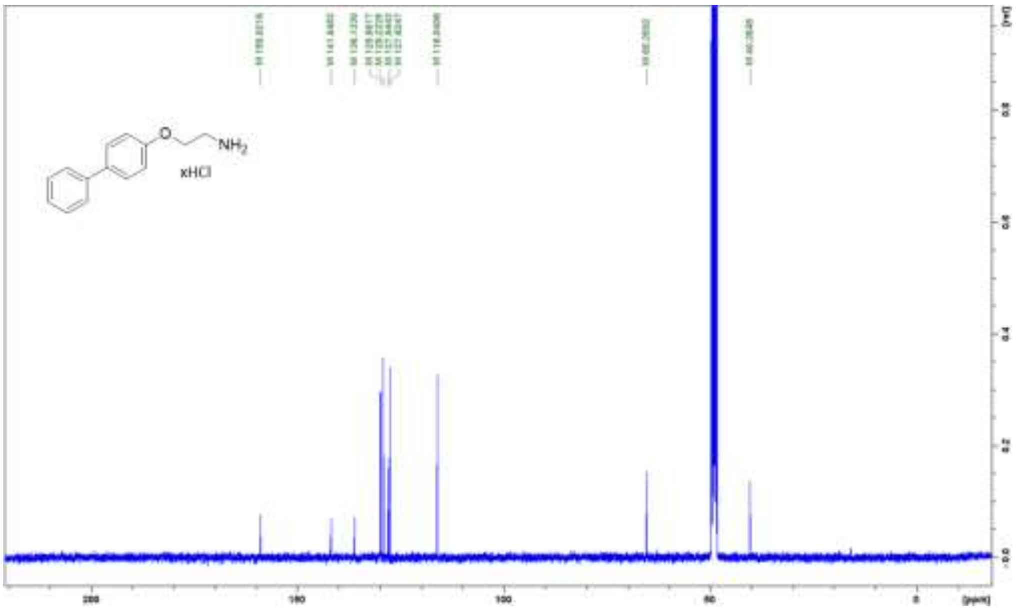

Figure **S11**. ^{1}H-NMR (400 MHz, CDCl3) spectrum of compound **5c**.

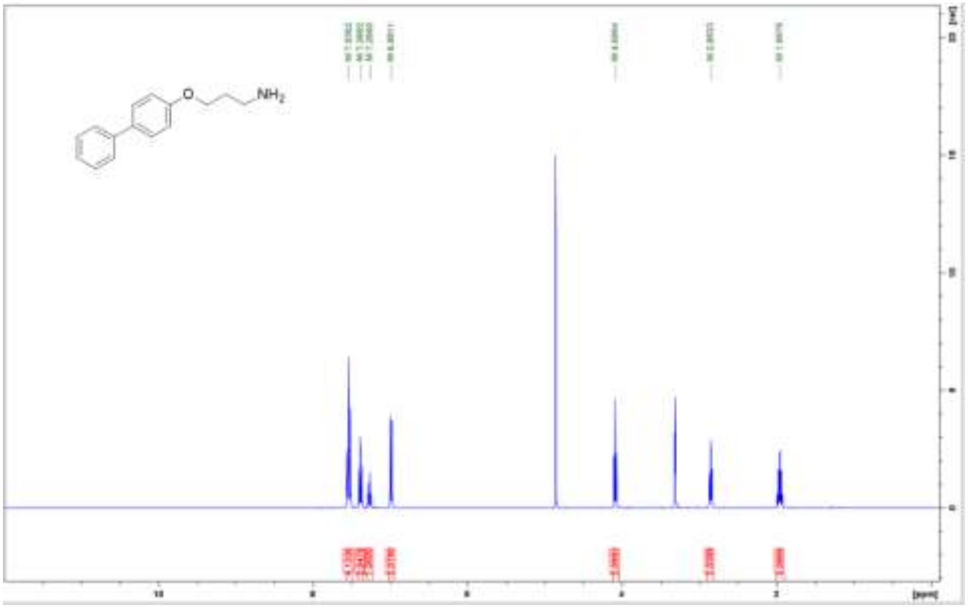

Figure **S12**. ^{13}C-NMR (100 MHz, CDCl3) spectrum of compound **5c**.

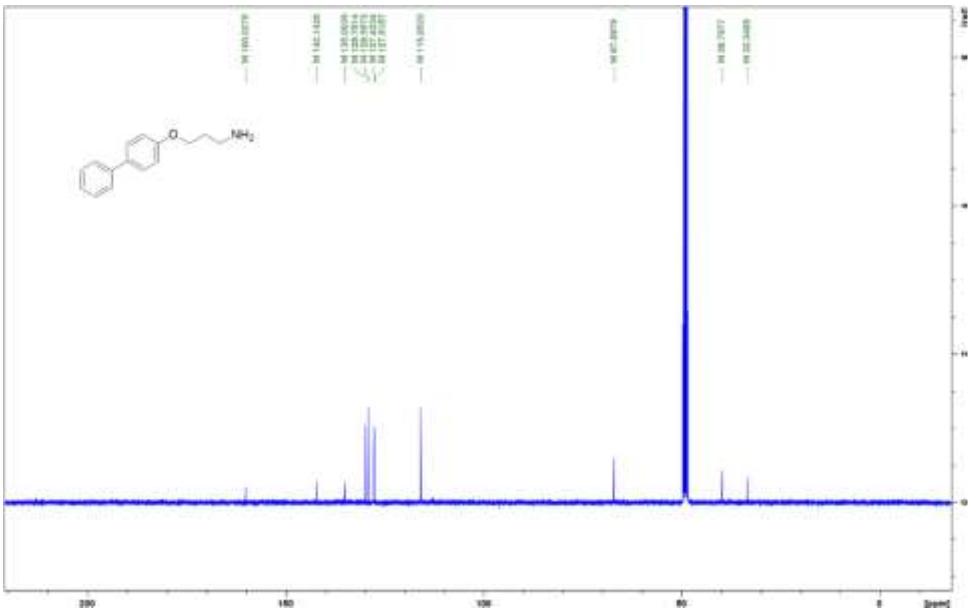

Figure **S13**. ^{1}H-NMR (400 MHz, CDCl3) spectrum of compound **5f**.

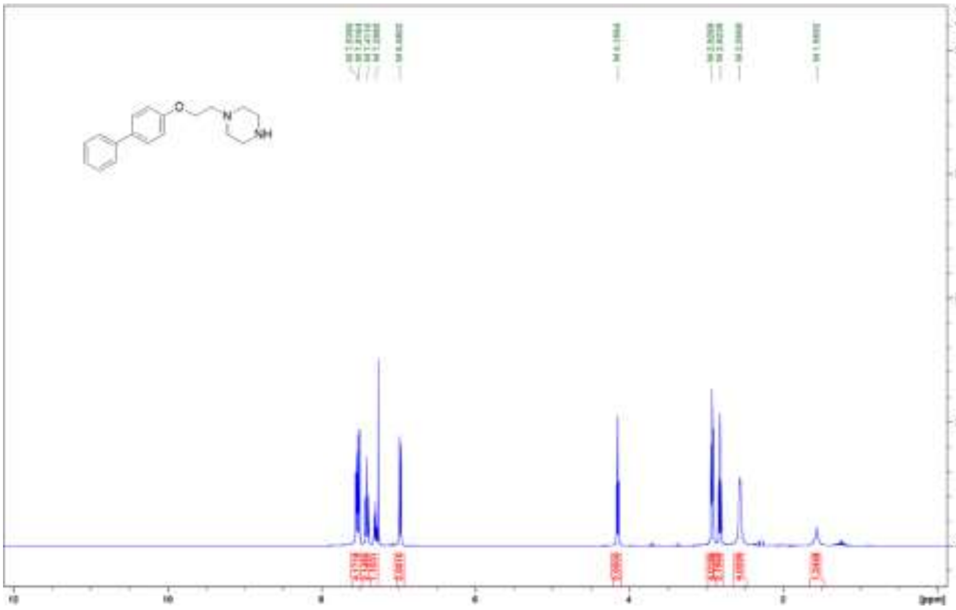

Figure **S14**. ^{13}C-NMR (100 MHz, CDCl3) spectrum of compound **5f**.

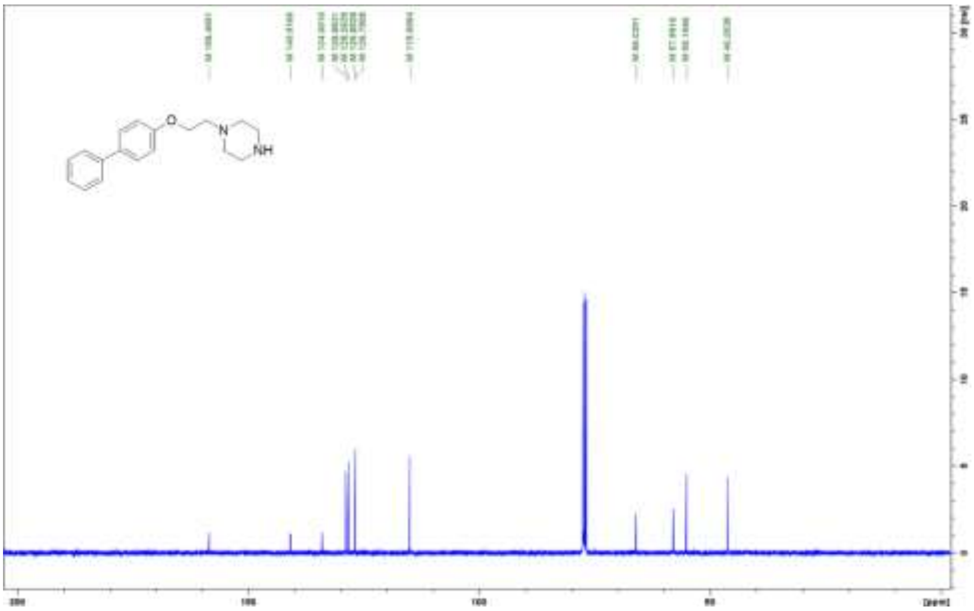

Figure **S15**. ^{1}H-NMR (400 MHz, CDCl3) spectrum of compound **5g**.

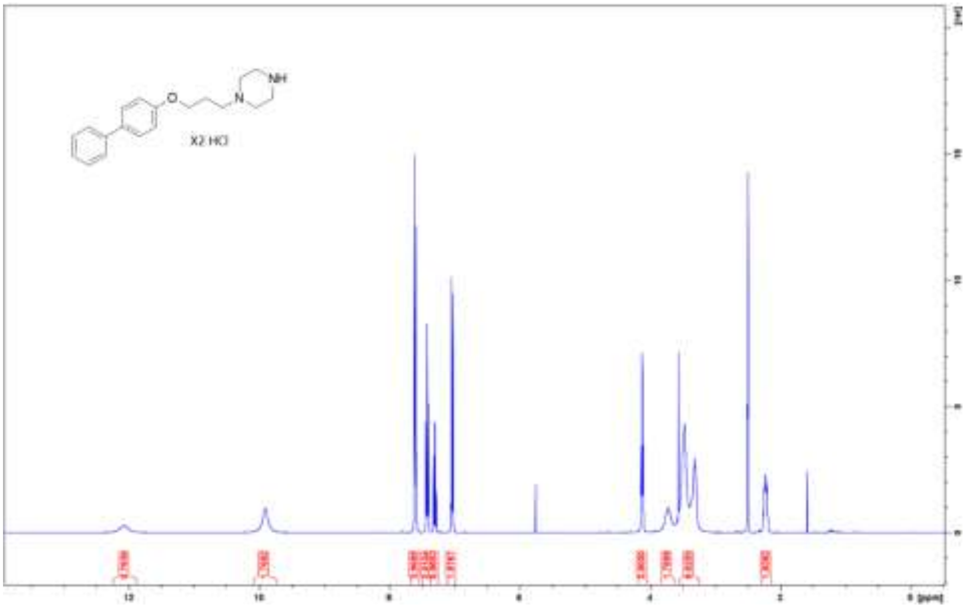

Figure **S16**. ^{13}C-NMR (100 MHz, CDCl3) spectrum of compound **5g**.

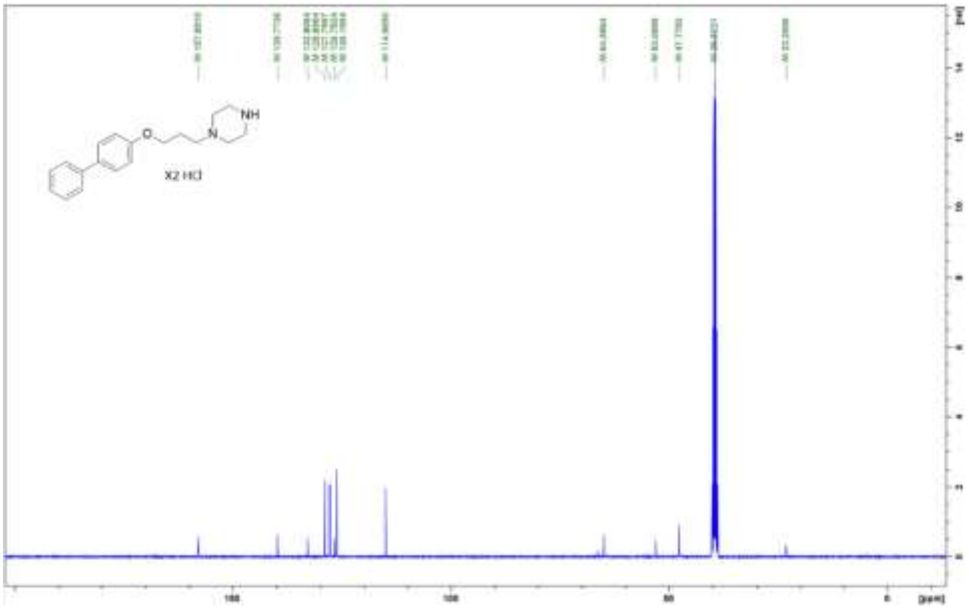

Figure **S17**. ^{1}H-NMR (400 MHz, CDCl3) spectrum of compound **7a**.

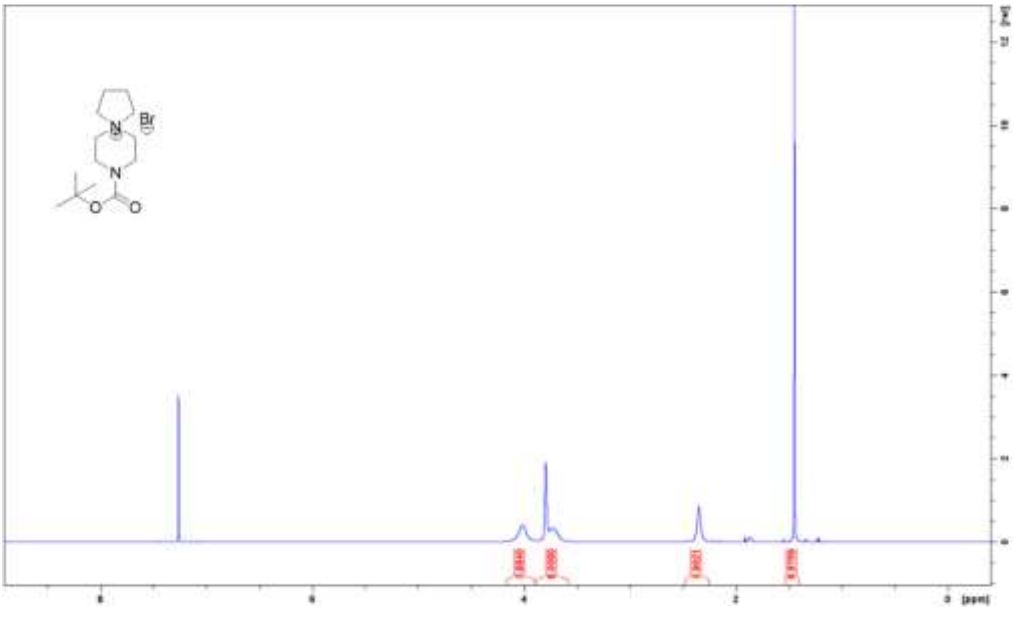

Figure **S18**. ^{13}C-NMR (100 MHz, CDCl3) spectrum of compound **7a**.

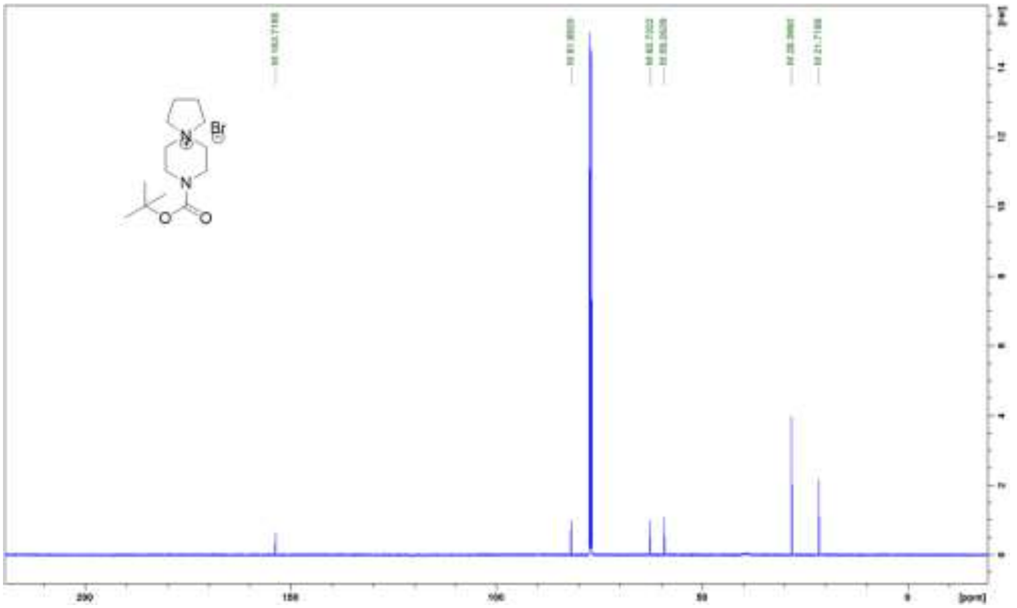

Figure **S19**. ^{1}H-NMR (400 MHz, CDCl3) spectrum of compound **7c**.

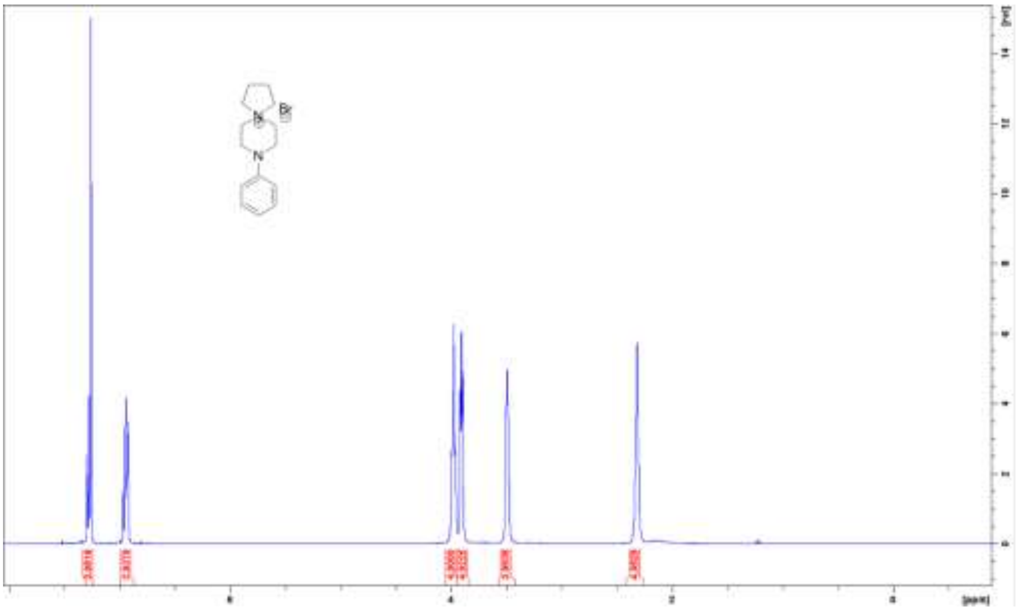

Figure **S20**. ^{13}C-NMR (100 MHz, CDCl3) spectrum of compound **7c**.

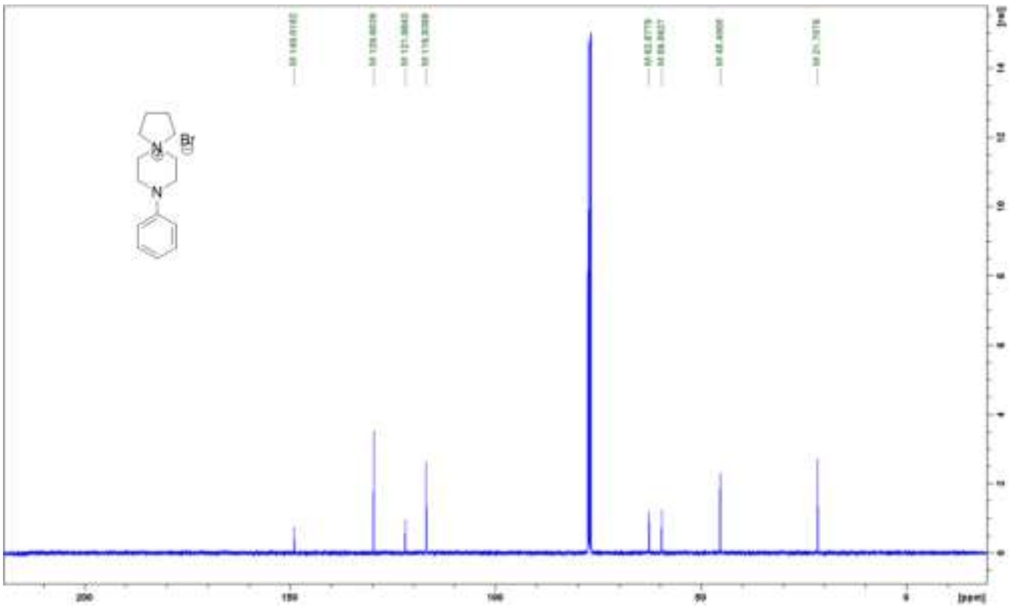

Figure **S21**. ^{1}H-NMR (400 MHz, CDCl3) spectrum of compound **8c**.

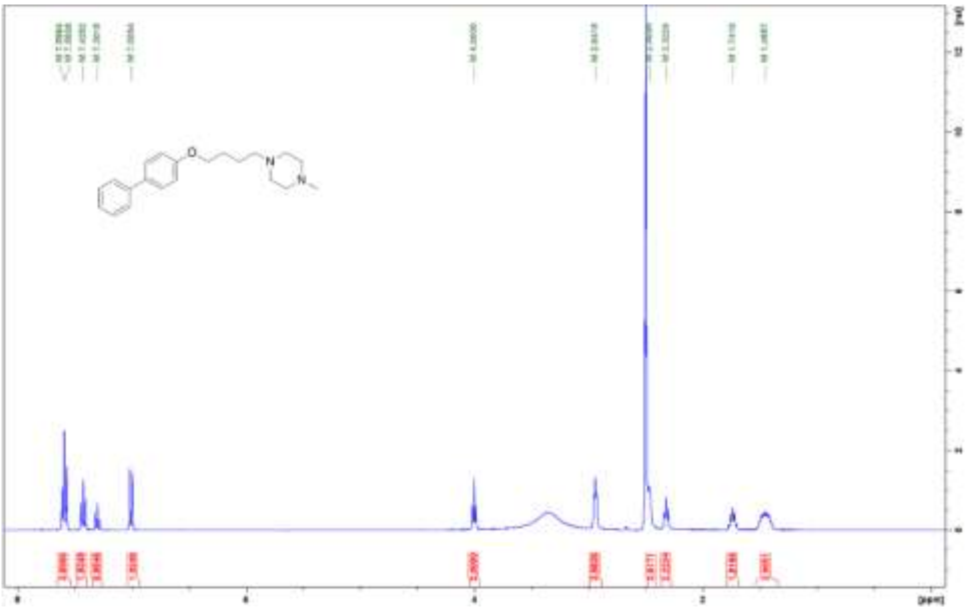

Figure **S22**. ^{13}C-NMR (100 MHz, CDCl3) spectrum of compound **8c**.

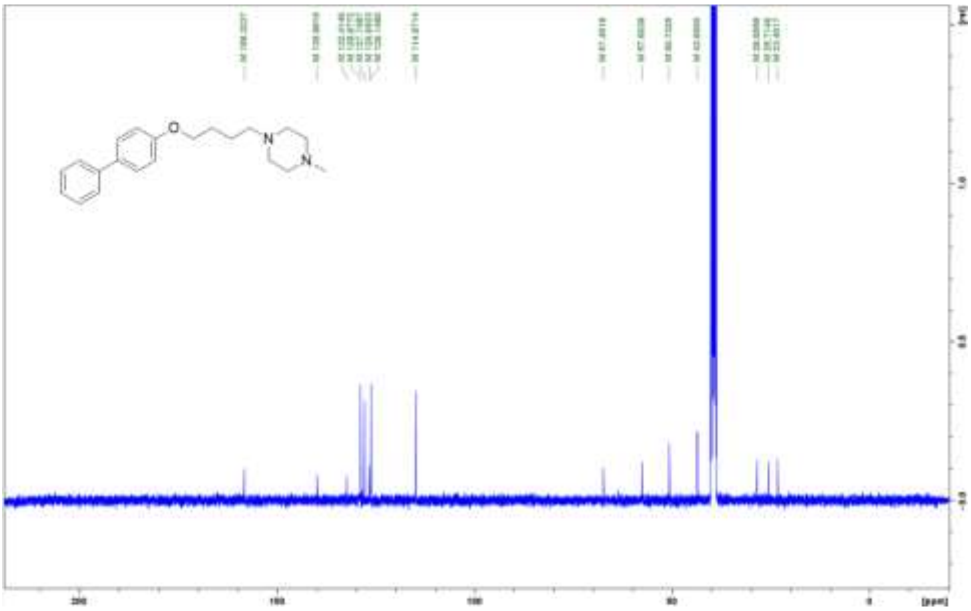

Figure **S23**. ^{1}H-NMR (400 MHz, CDCl3) spectrum of compound **9a**.

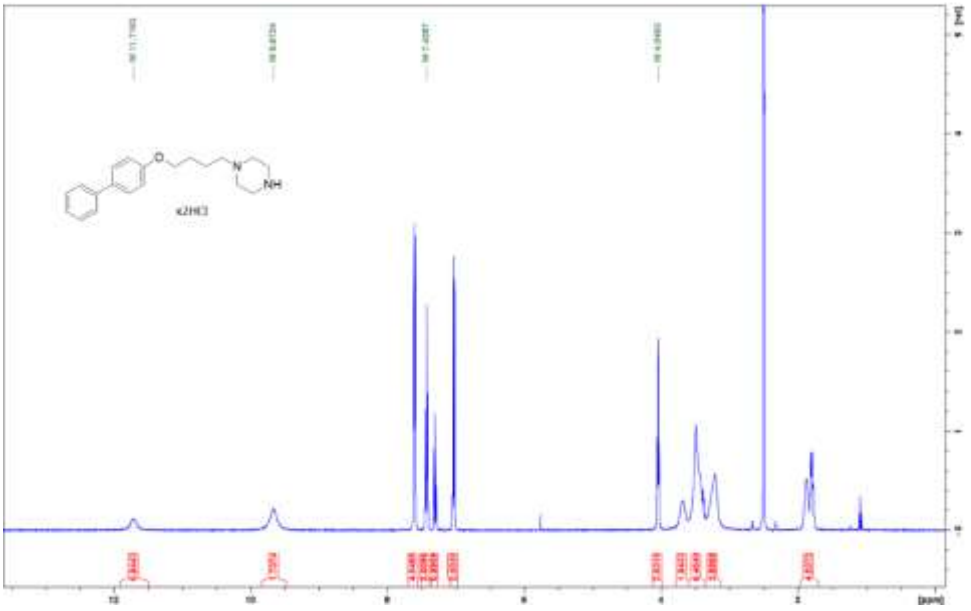

Figure **S24**. ^{13}C-NMR (100 MHz, CDCl3) spectrum of compound **9a**.

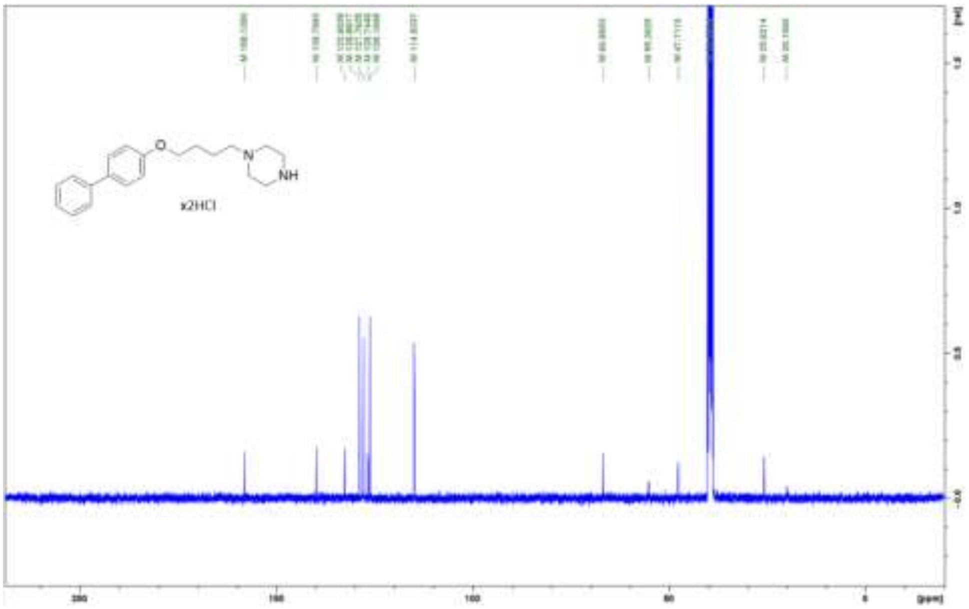

Figure **S25**. ^{1}H-NMR (400 MHz, CDCl3) spectrum of compound **9b**

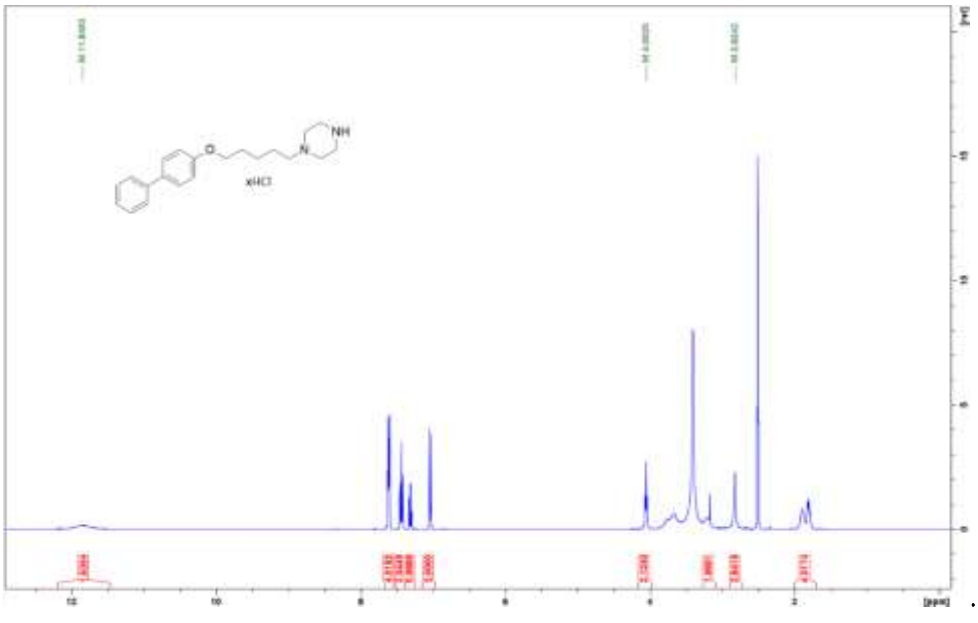

Figure **S26**. ^{13}C-NMR (100 MHz, CDCl3) spectrum of compound **9b**.

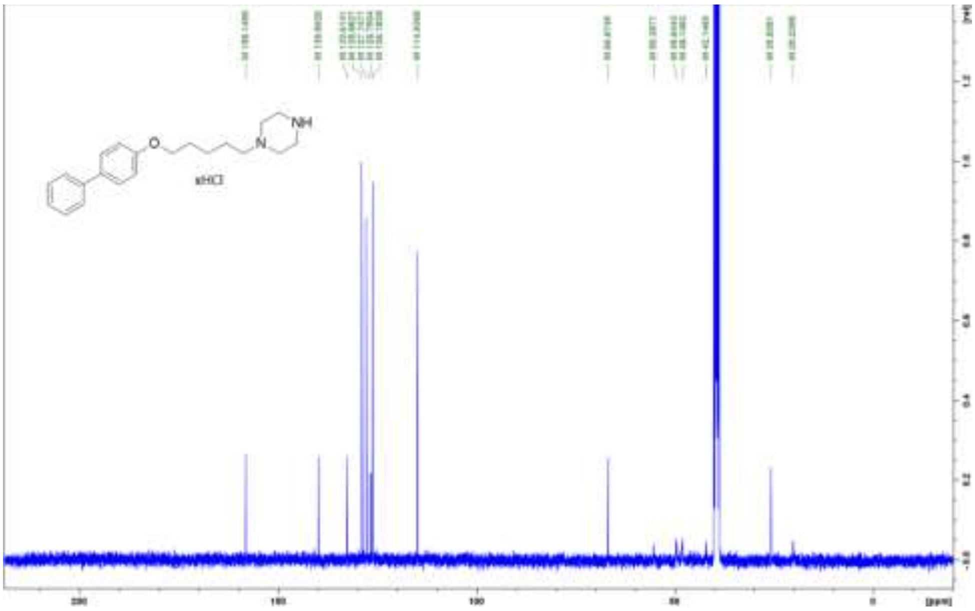

Figure **S27**. ^{1}H-NMR (400 MHz, CDCl3) spectrum of compound **11**.

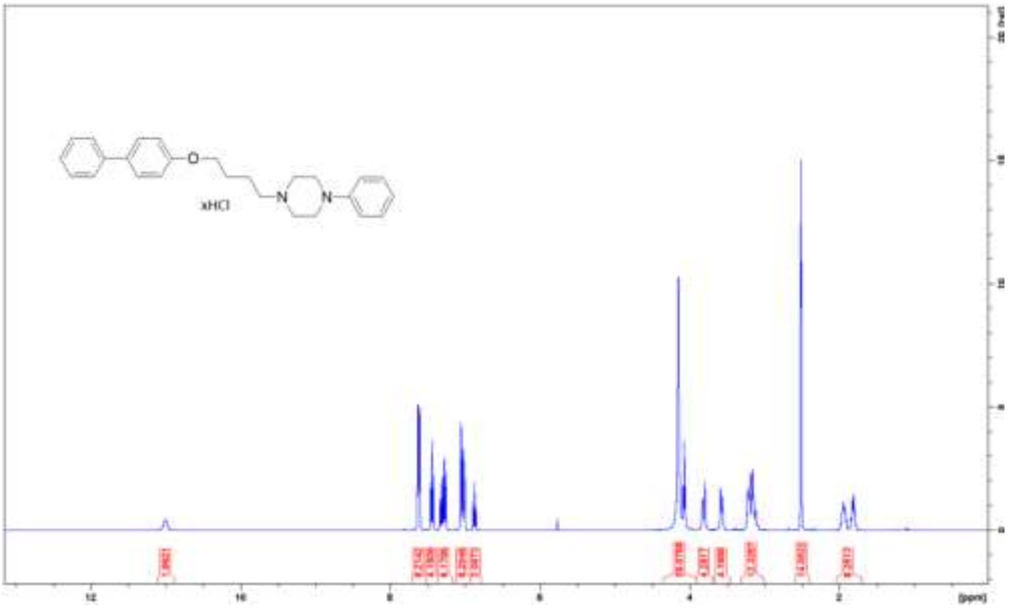

Figure **S28**. ^{13}C-NMR (100 MHz, CDCl3) spectrum of compound **11**.

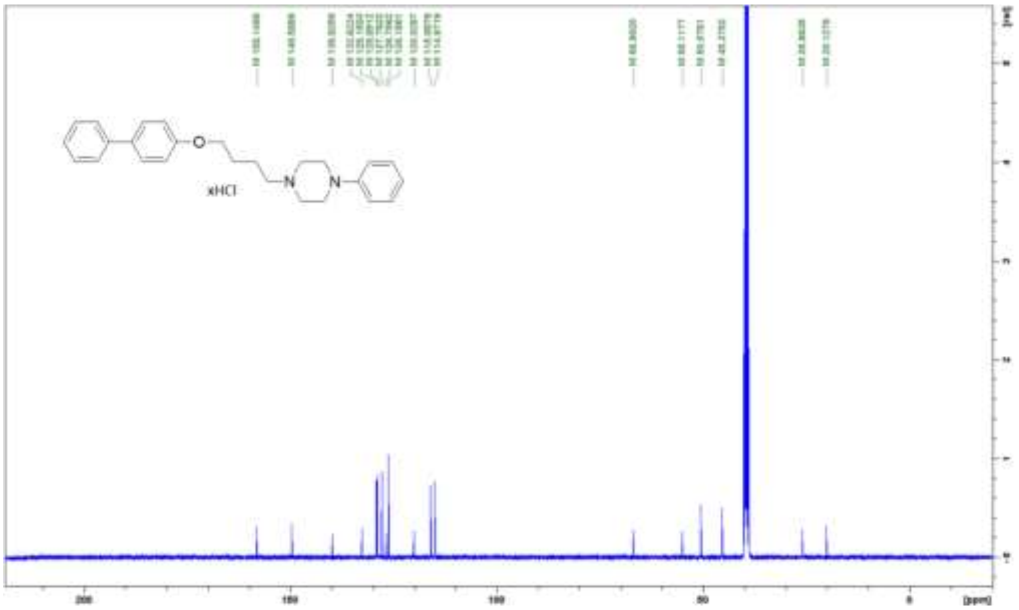

Figure **S29**. ^{1}H-NMR (400 MHz, CDCl3) spectrum of compound **13a**.

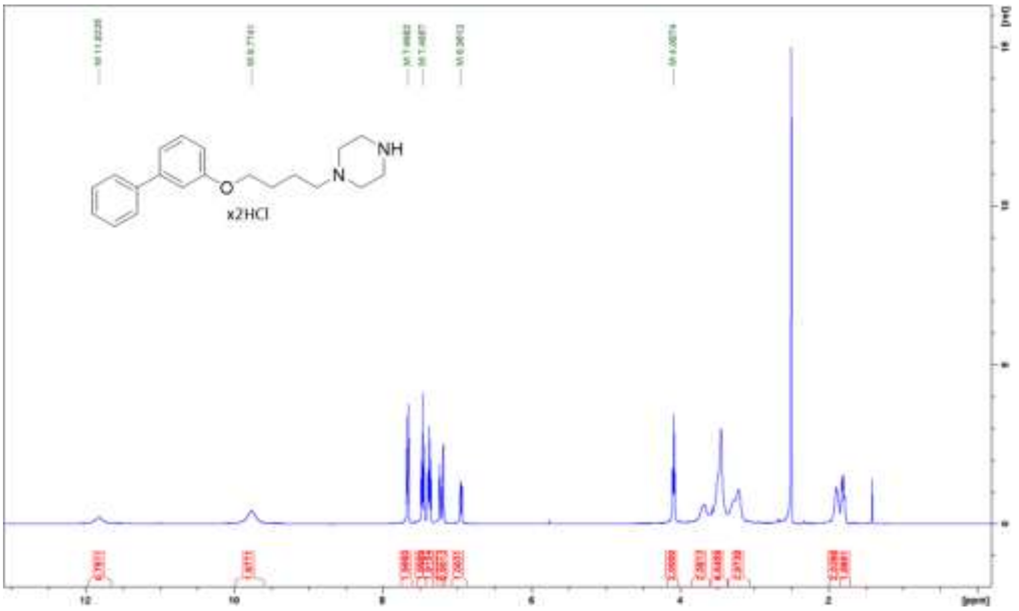

Figure **S30**. ^{13}C-NMR (100 MHz, CDCl3) spectrum of compound **13a**.

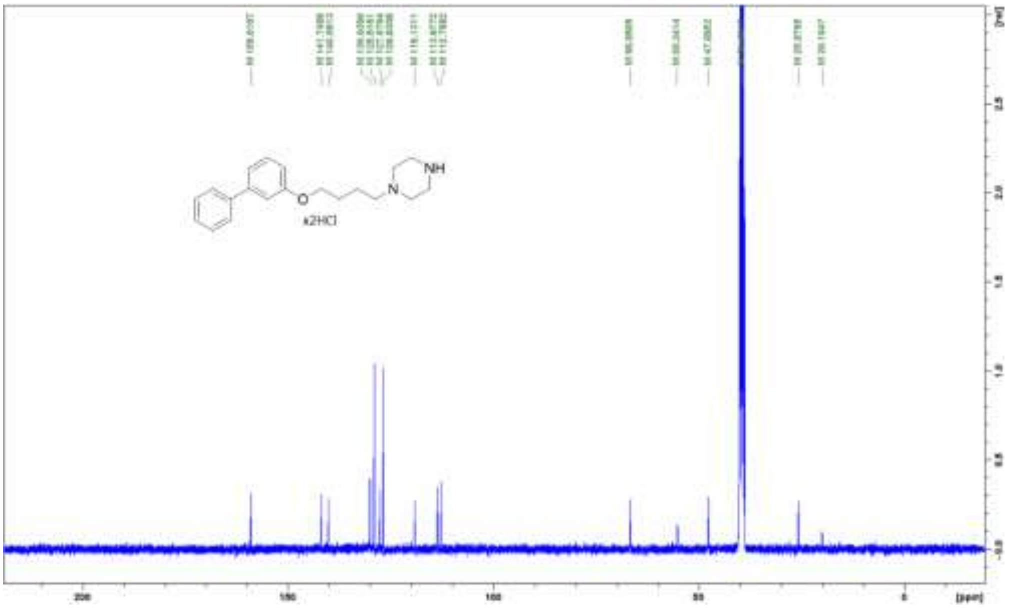

Figure **S31**. ^{1}H-NMR (400 MHz, CDCl3) spectrum of compound **13b**.

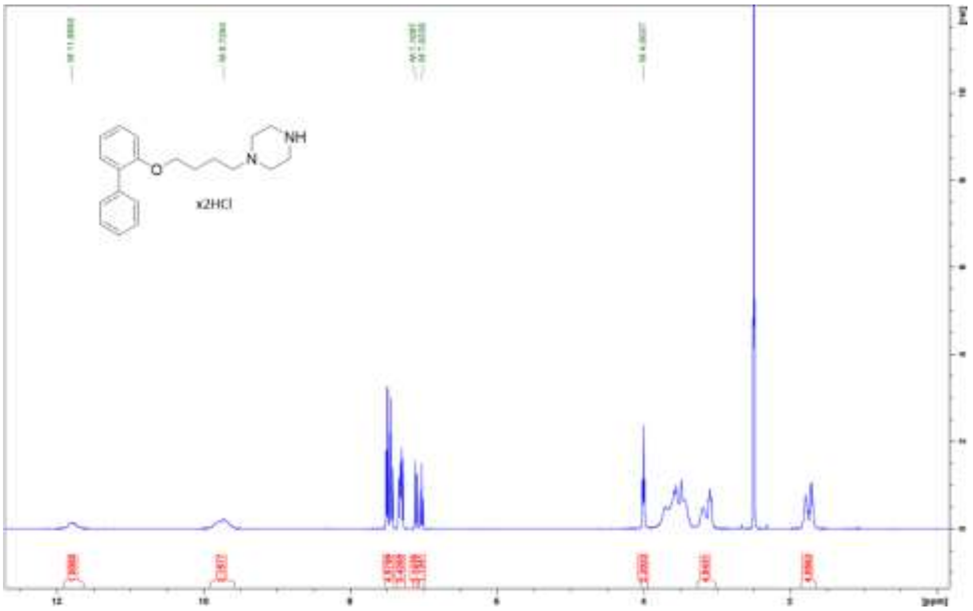

Figure **S32**. ^{13}C-NMR (100 MHz, CDCl3) spectrum of compound **13b**.

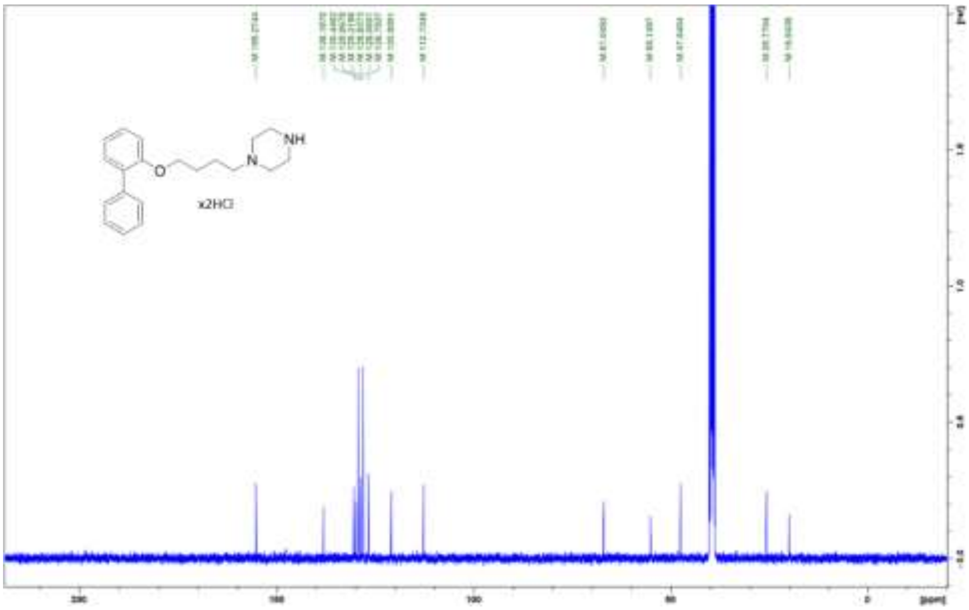

Figure **S33**. ^{1}H-NMR (400 MHz, CDCl3) spectrum of compound **13c**.

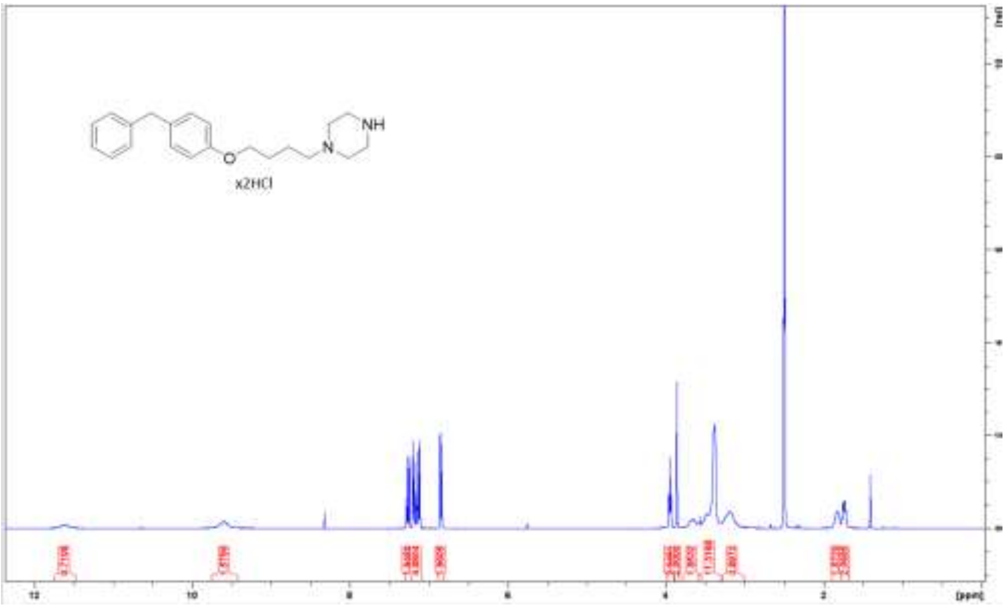

Figure **S34**. ^{13}C-NMR (100 MHz, CDCl3) spectrum of compound **13c**.

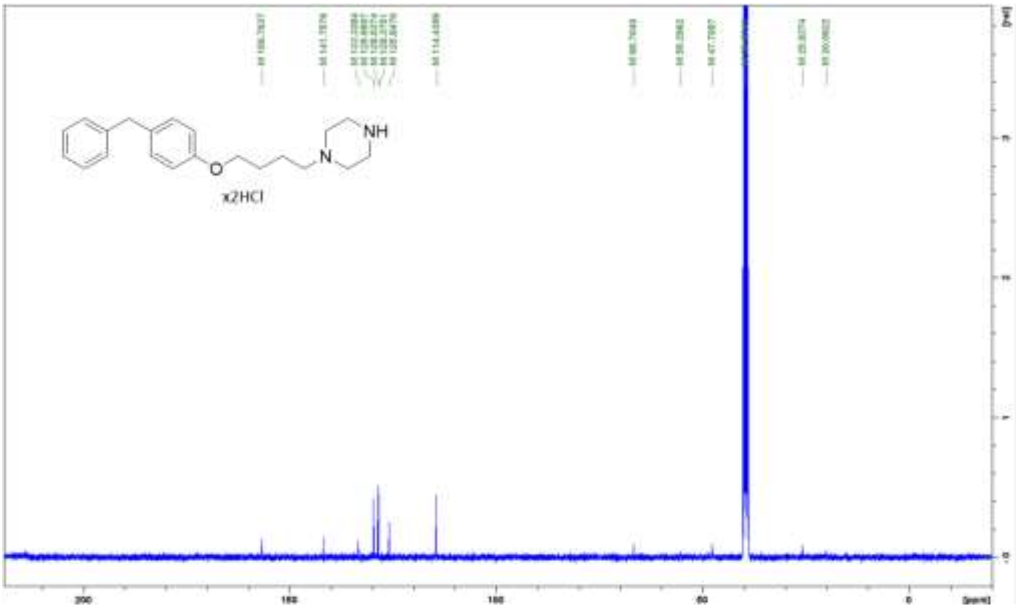

Figure **S35**. ^{1}H-NMR (400 MHz, CDCl3) spectrum of compound **13d**.

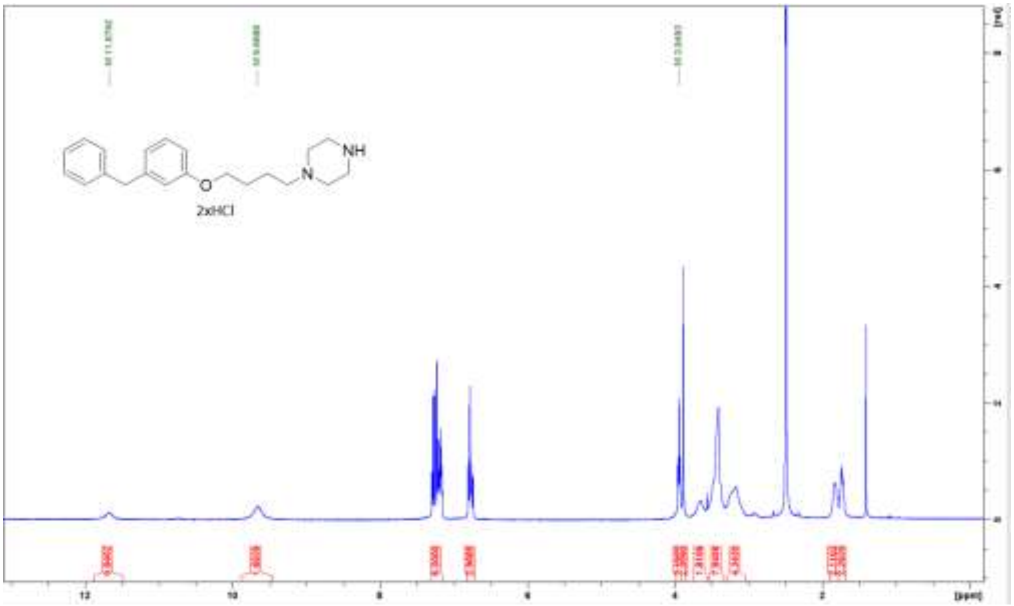

Figure **S36**. ^{13}C-NMR (100 MHz, CDCl3) spectrum of compound **13d**.

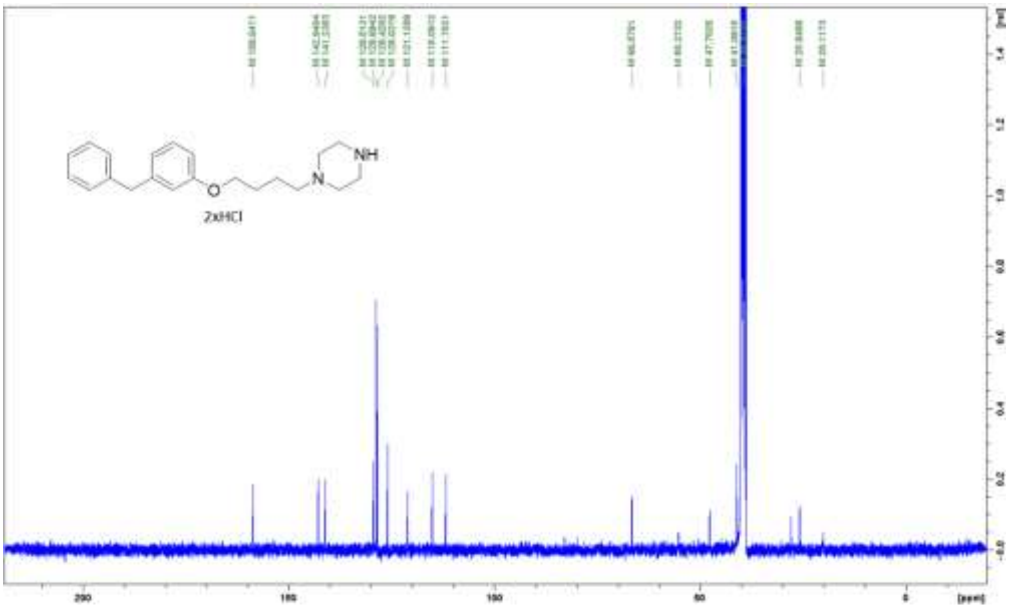

Figure **S37**. ^{1}H-NMR (400 MHz, CDCl3) spectrum of compound **13e**.

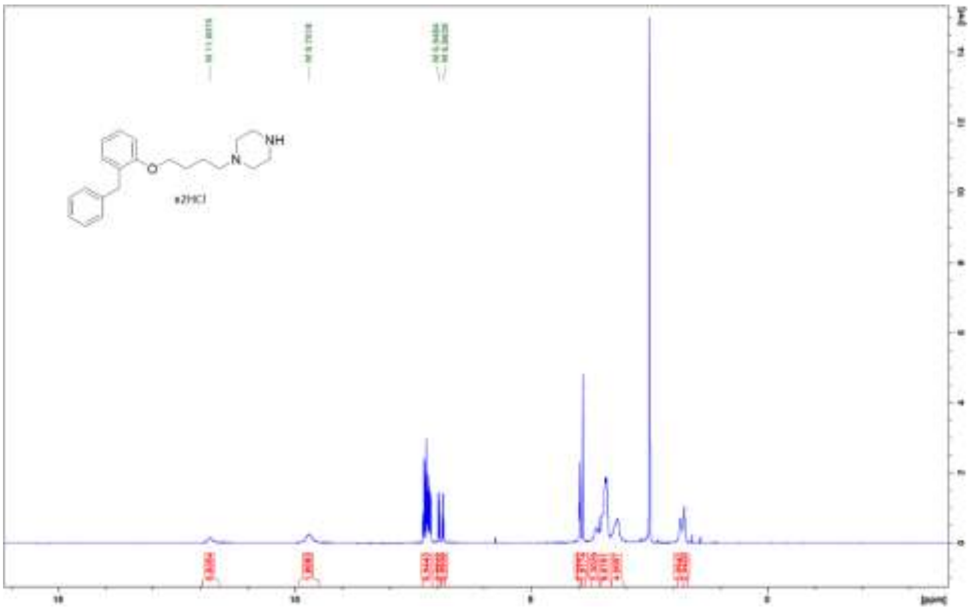

Figure **S38**. ^{13}C-NMR (100 MHz, CDCl3) spectrum of compound **13e**.

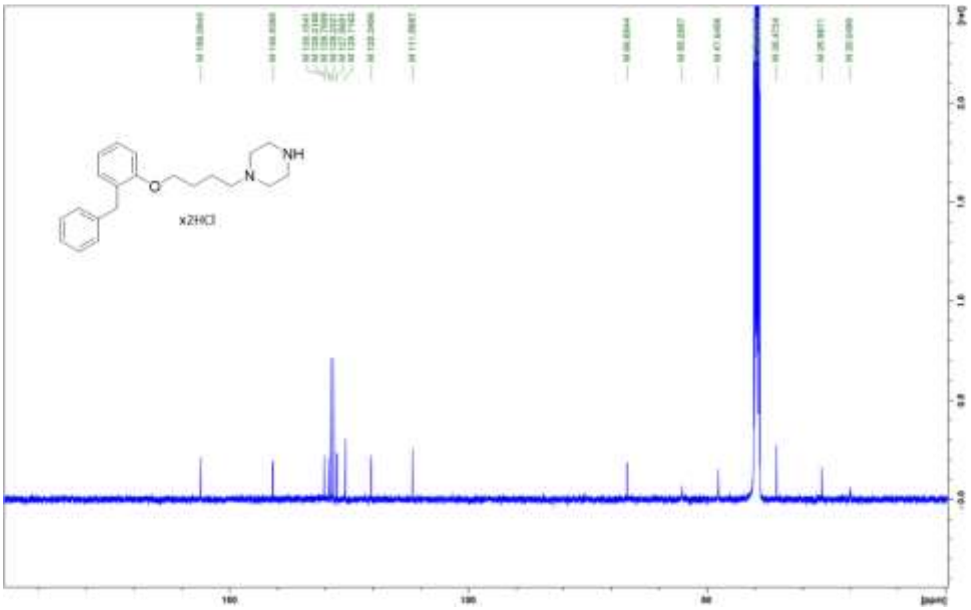

Figure **S39**. ^{1}H-NMR (400 MHz, CDCl3) spectrum of compound **13f**.

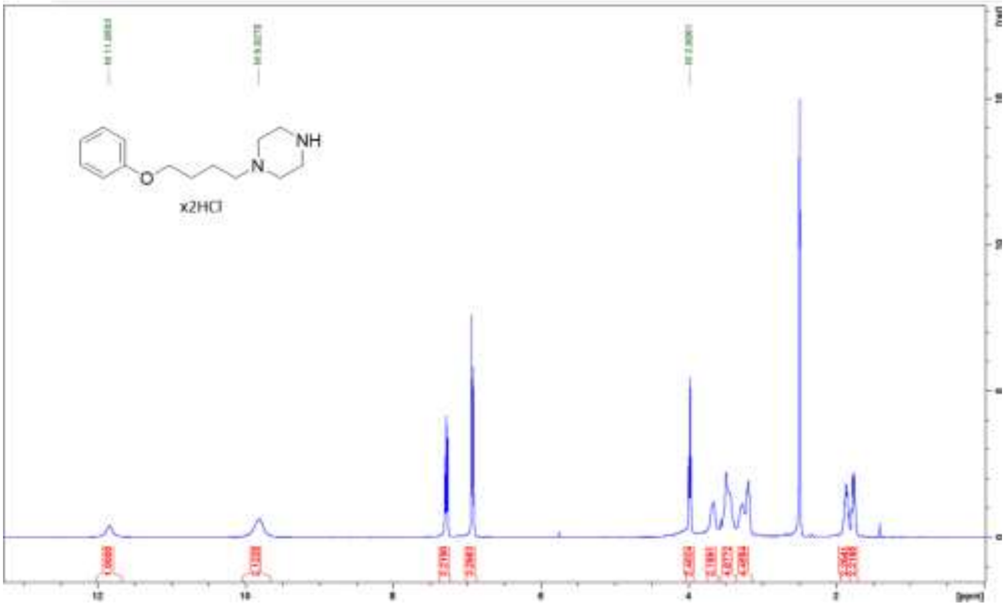

Figure **S40**. ^{13}C-NMR (100 MHz, CDCl3) spectrum of compound **13f**.

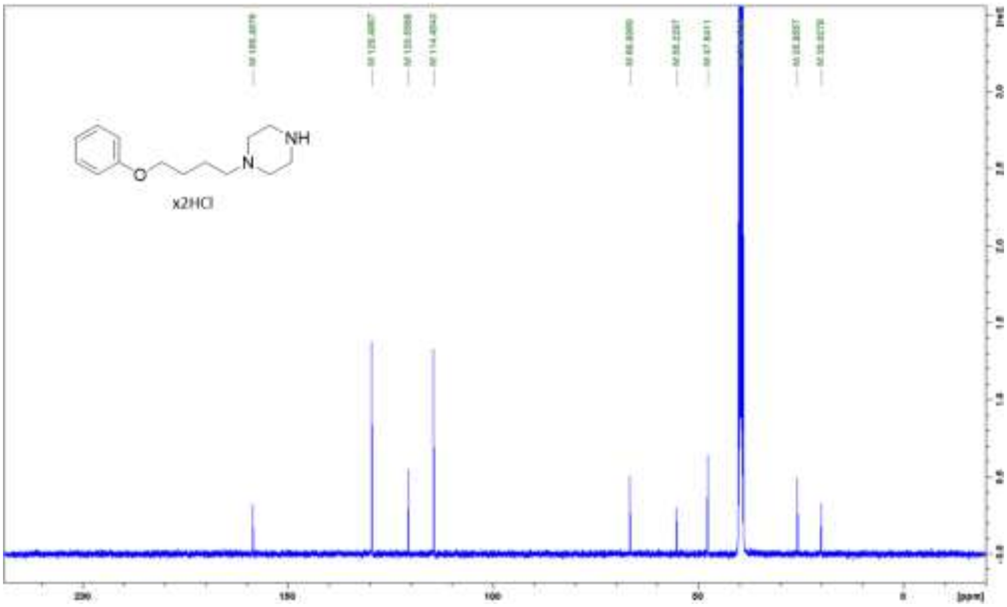

Figure **S41**. ^{1}H-NMR (400 MHz, CDCl3) spectrum of compound **13g**.

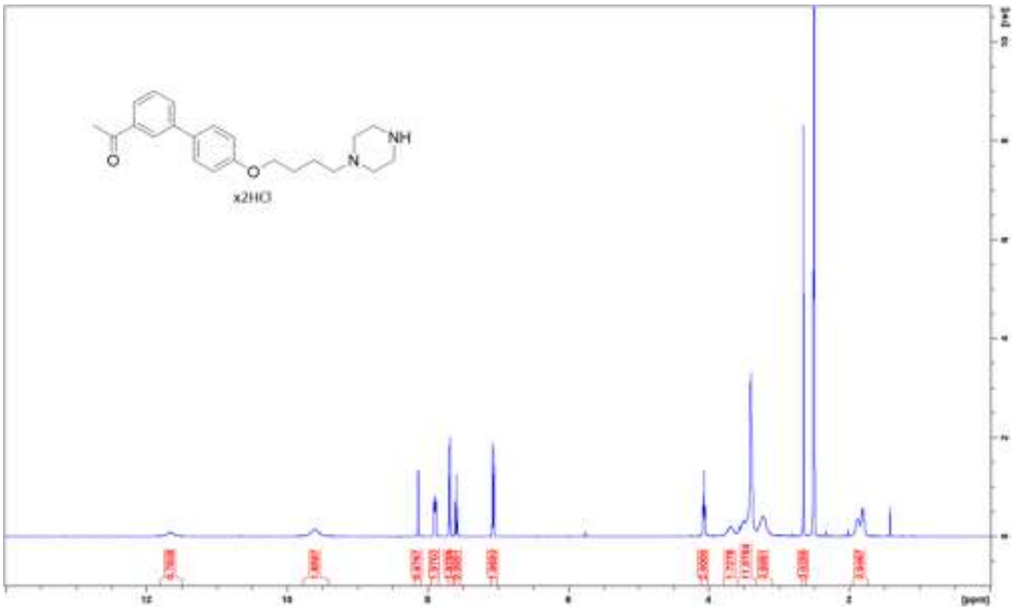

Figure **S42**. ^{13}C-NMR (100 MHz, CDCl3) spectrum of compound **13g**.

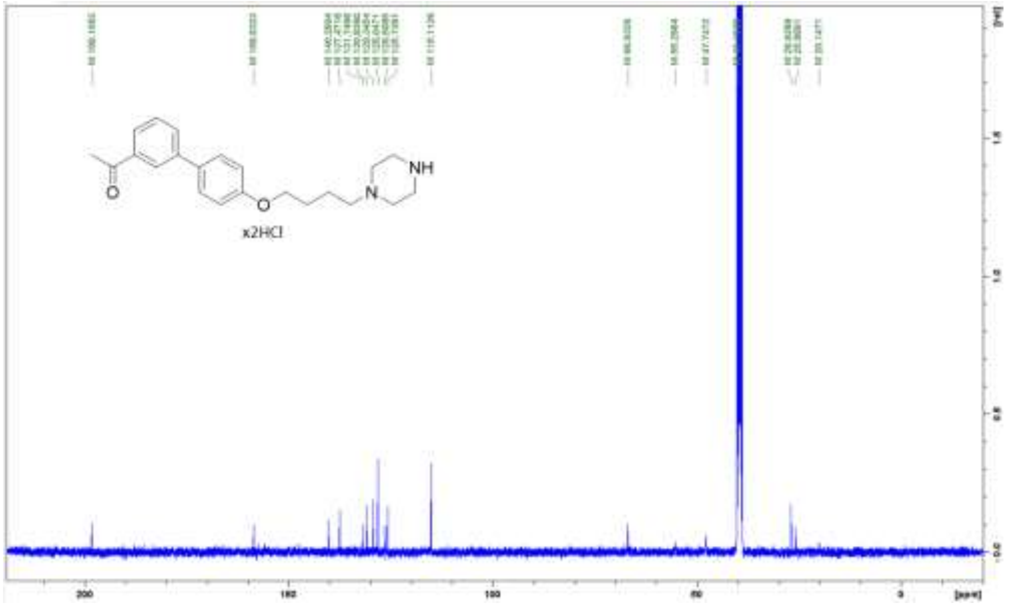

Figure **S43**. ^{1}H-NMR (400 MHz, CDCl3) spectrum of compound **13h**.

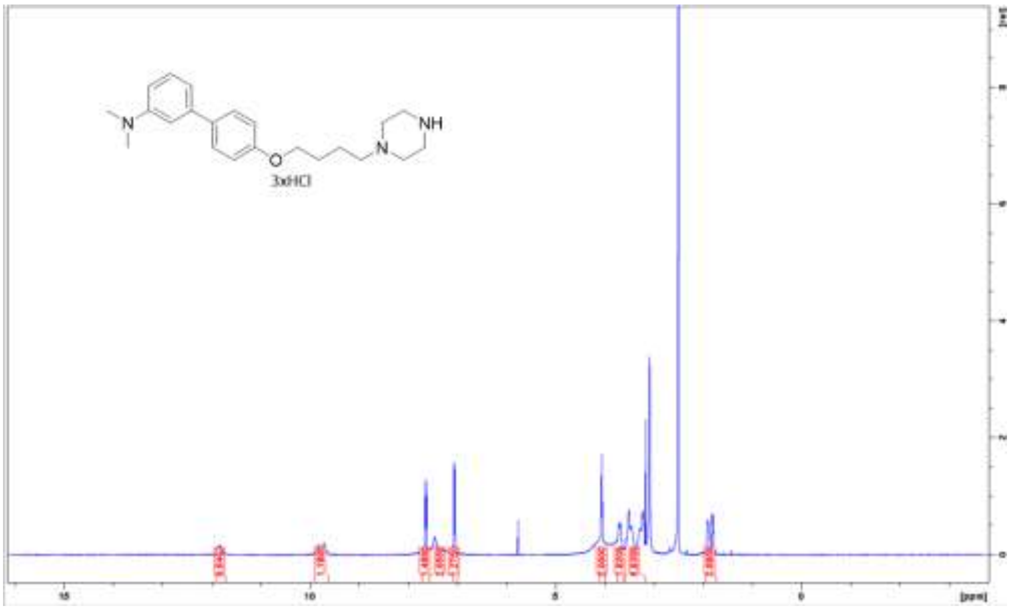

Figure **S44**. ^{13}C-NMR (100 MHz, CDCl3) spectrum of compound **13h**.

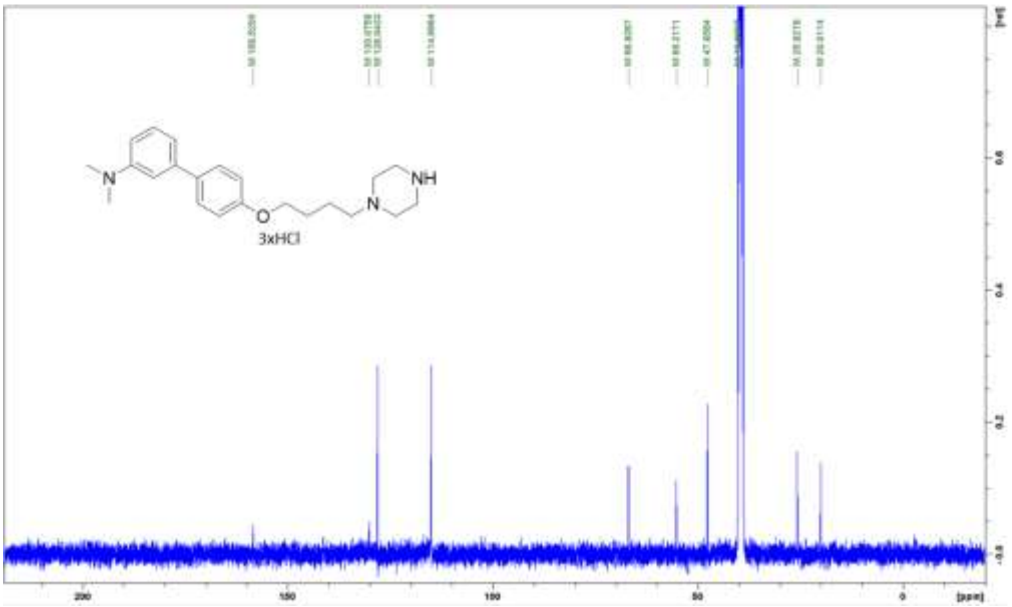

Figure **S45**. ^{1}H-NMR (400 MHz, CDCl3) spectrum of compound **13i**.

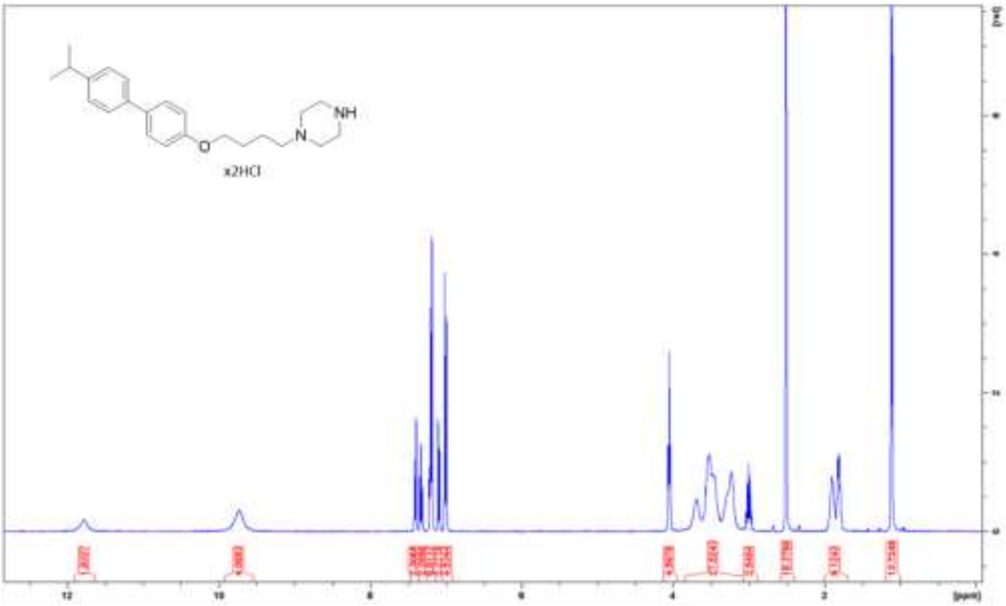

Figure **S46**. ^{13}C-NMR (100 MHz, CDCl3) spectrum of compound **13i**.

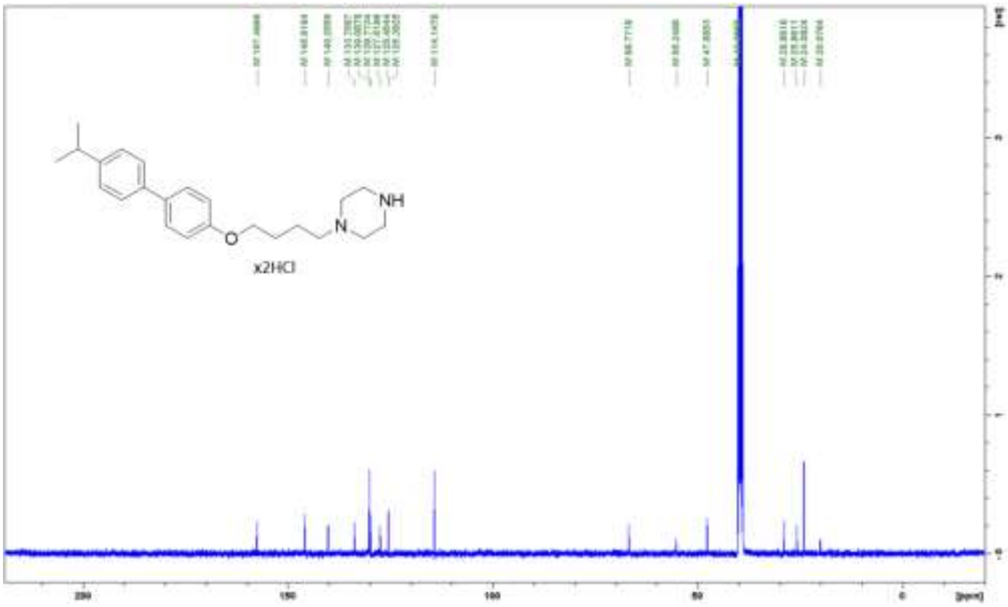

Figure **S47**. ^{1}H-NMR (400 MHz, CDCl3) spectrum of compound **13j**.

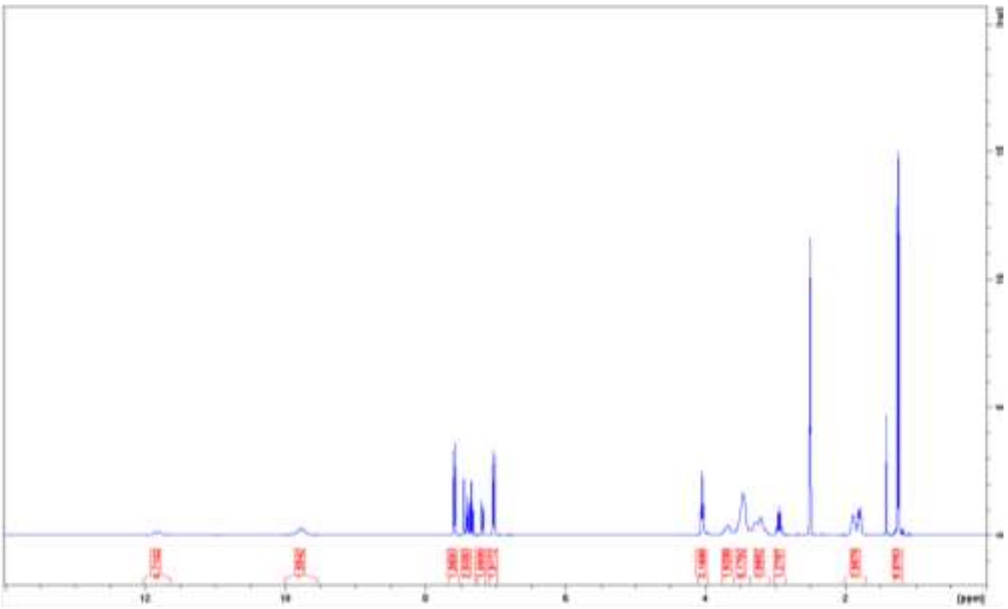

Figure **S48**. ^{13}C-NMR (100 MHz, CDCl3) spectrum of compound **13j**.

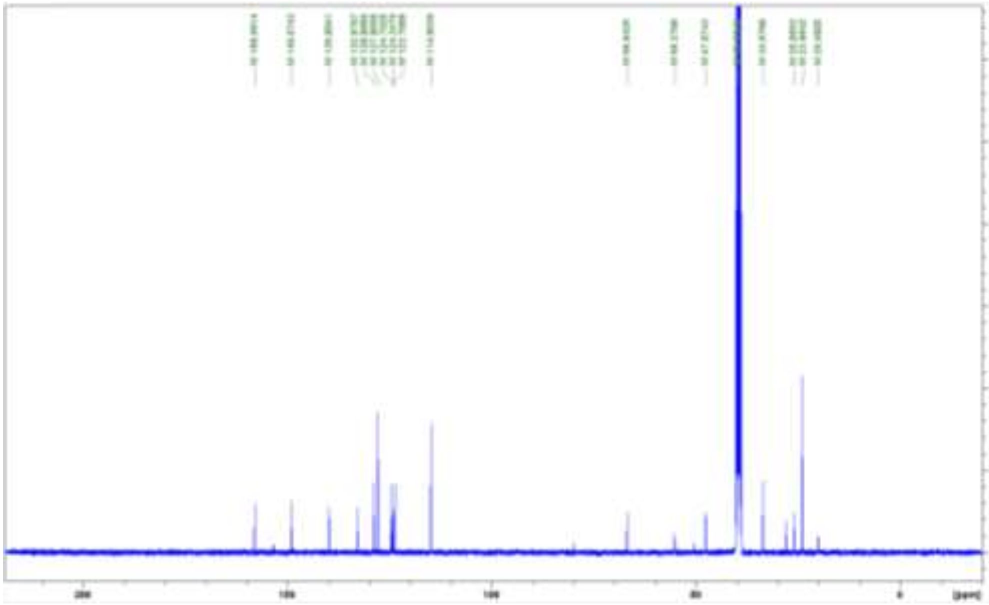

Figure **S49**. ^{1}H-NMR (400 MHz, CDCl3) spectrum of compound **13k**.

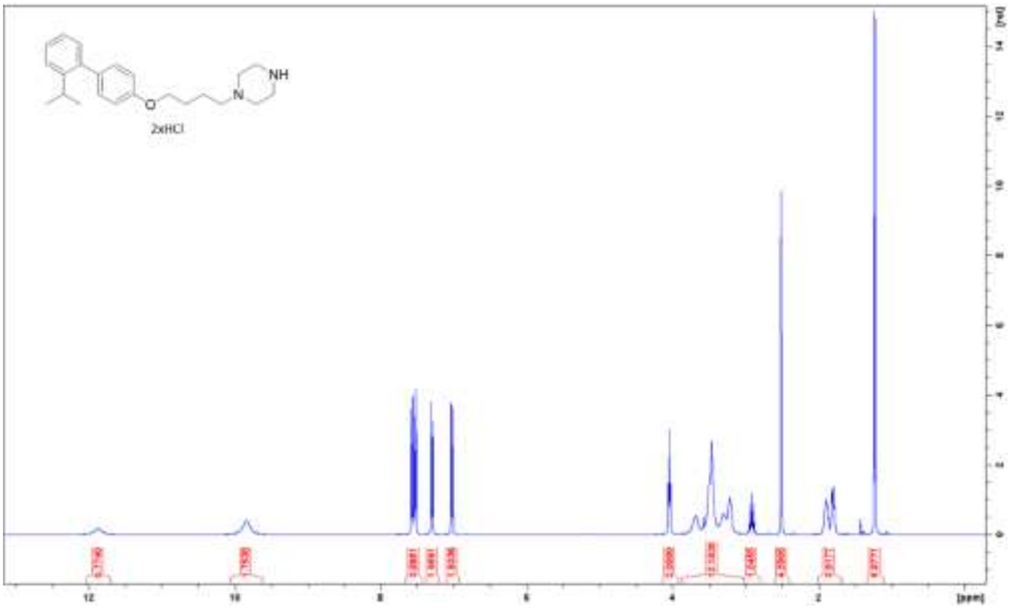

Figure **S50**. ^{13}C-NMR (100 MHz, CDCl3) spectrum of compound **13k**.

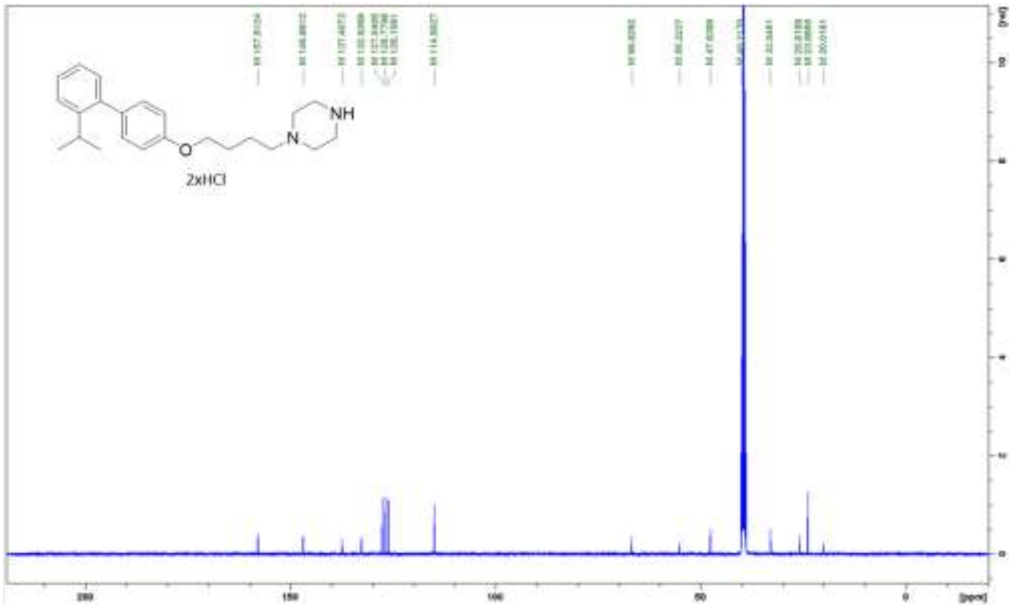

Figure **S51**. ^{1}H-NMR (400 MHz, CDCl3) spectrum of compound **13l**.

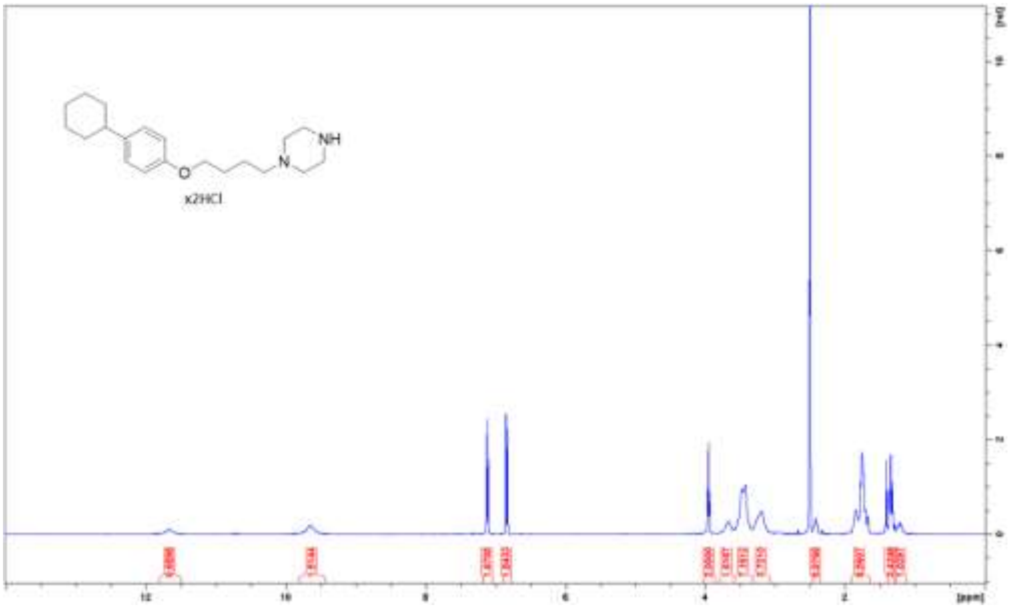

Figure **S52**. ^{13}C-NMR (100 MHz, CDCl3) spectrum of compound **13l**.

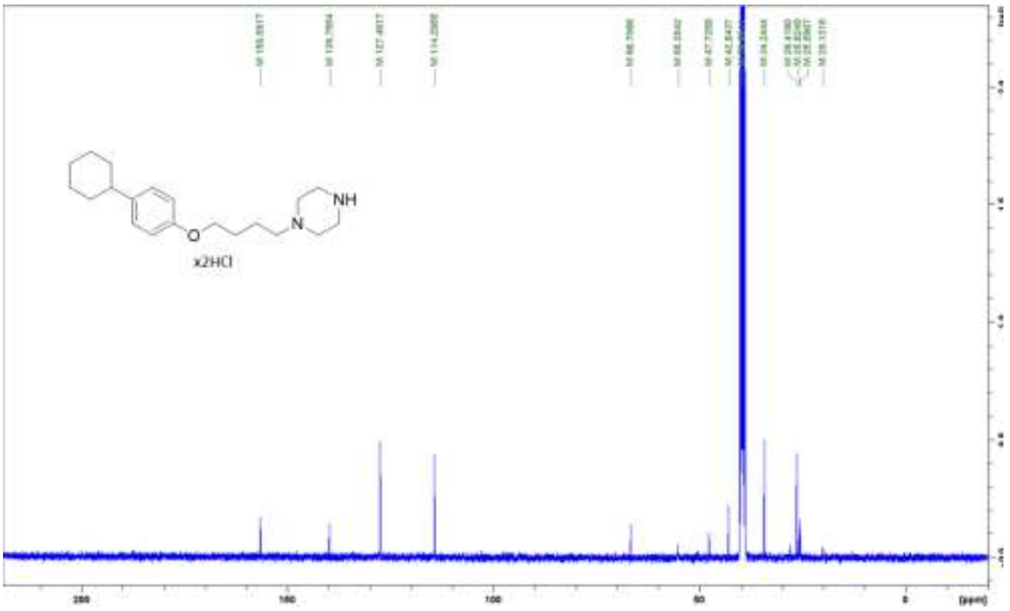

Figure **S53**. ^{1}H-NMR (400 MHz, CDCl3) spectrum of compound **13m**.

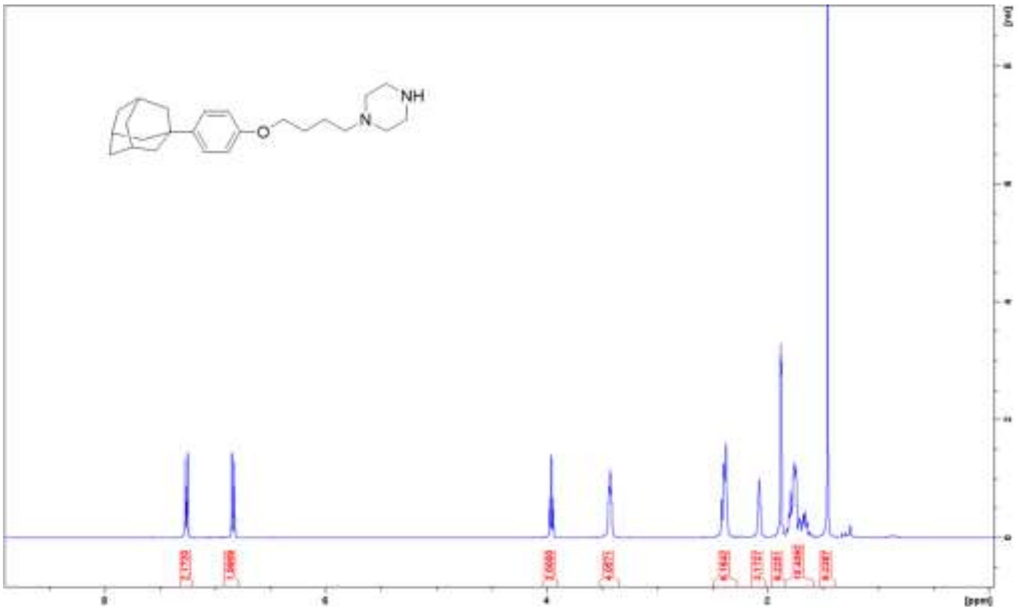

Figure **S54**. ^{13}C-NMR (100 MHz, CDCl3) spectrum of compound **13m**.

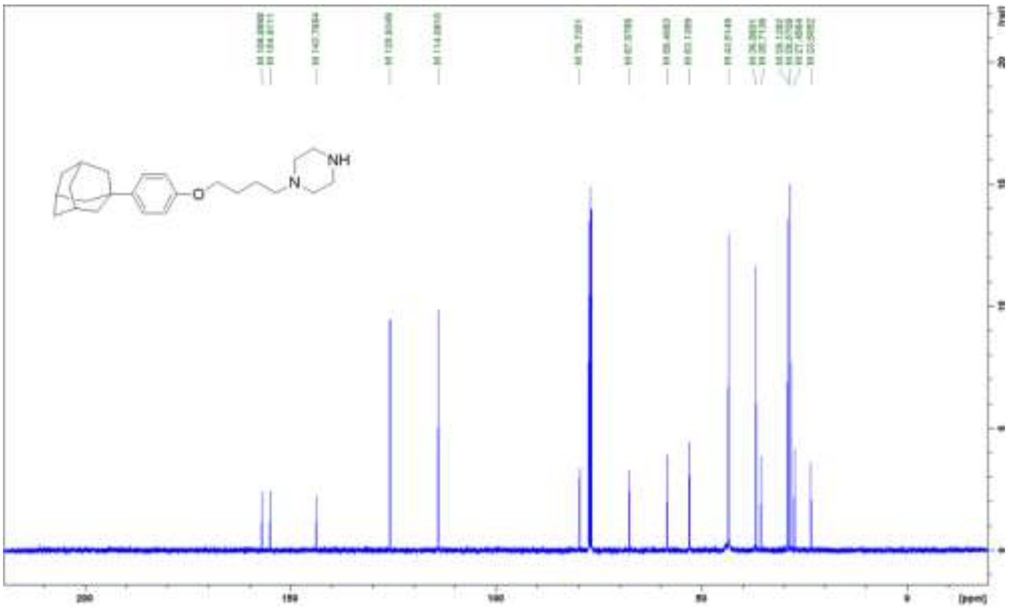

Figure **S55**. ^{1}H-NMR (400 MHz, CDCl3) spectrum of compound **16**.

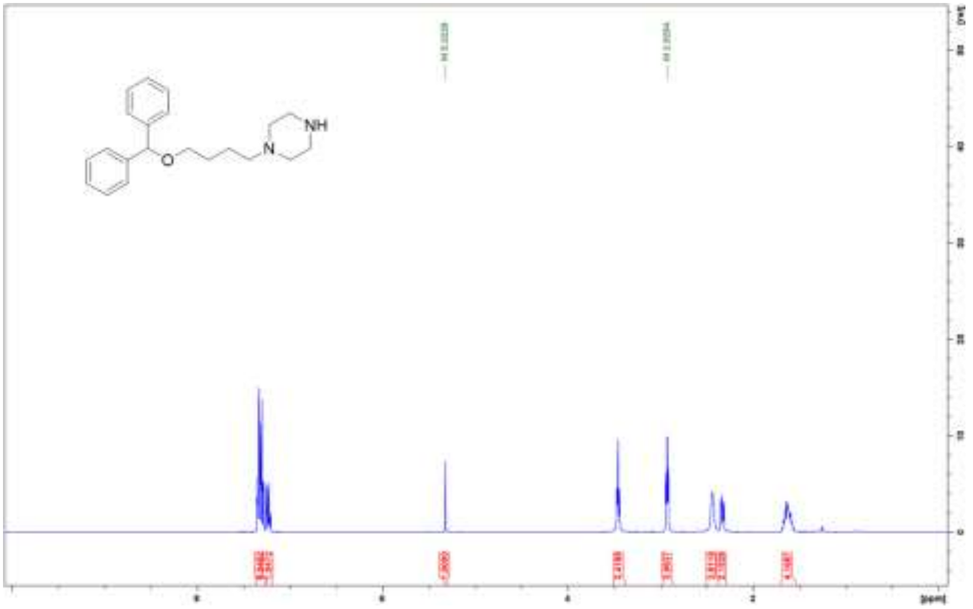

Figure **S56**. ^{13}C-NMR (100 MHz, CDCl3) spectrum of compound **16**.

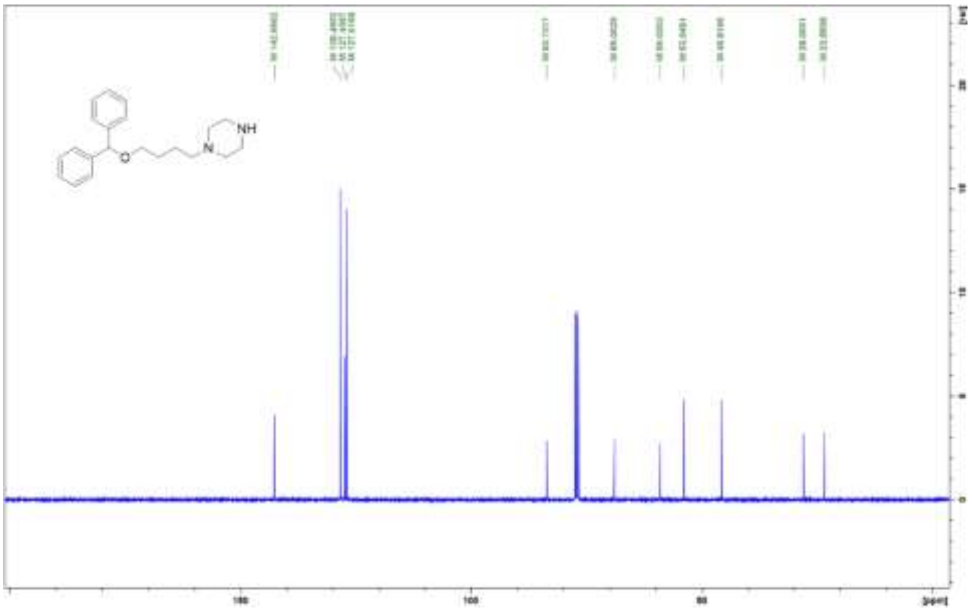

Figure **S57**. HPLC chromatogram of compound **1**

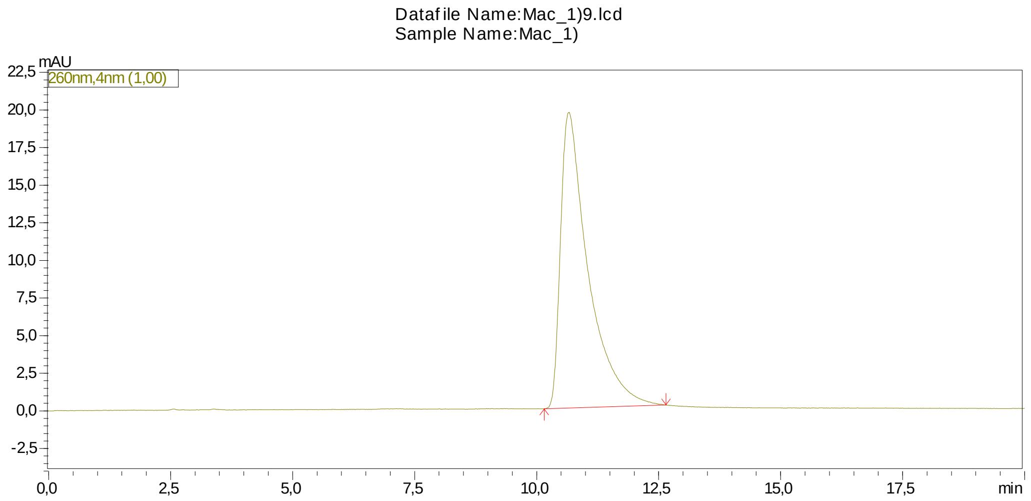

Peak#	Ret. Time	Area	Height	Conc.	Name	Area%
1	10,666	772297	19652	0,000		100,000
Total		772297	19652	0,000		100,000

Figure **S58**. HPLC chromatogram of compound **4d**

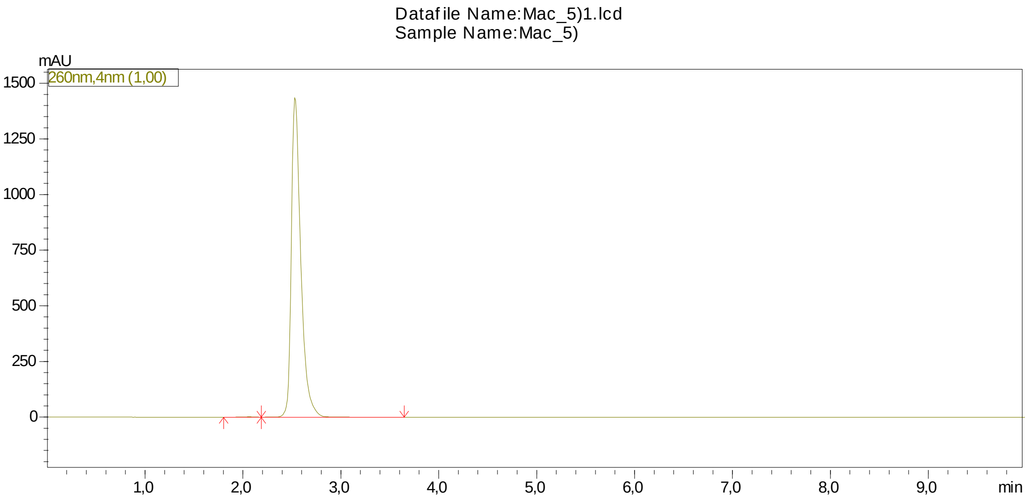

Peak#	Ret. Time	Area	Height	Conc.	Name	Area%
1	2,068	12523	1618	0,000		0,129
2	2,532	9690482	1435756	0,000		99,871
Total		9703005	1437374	0,000		100,000

Figure **S59**. HPLC chromatogram of compound **4e**

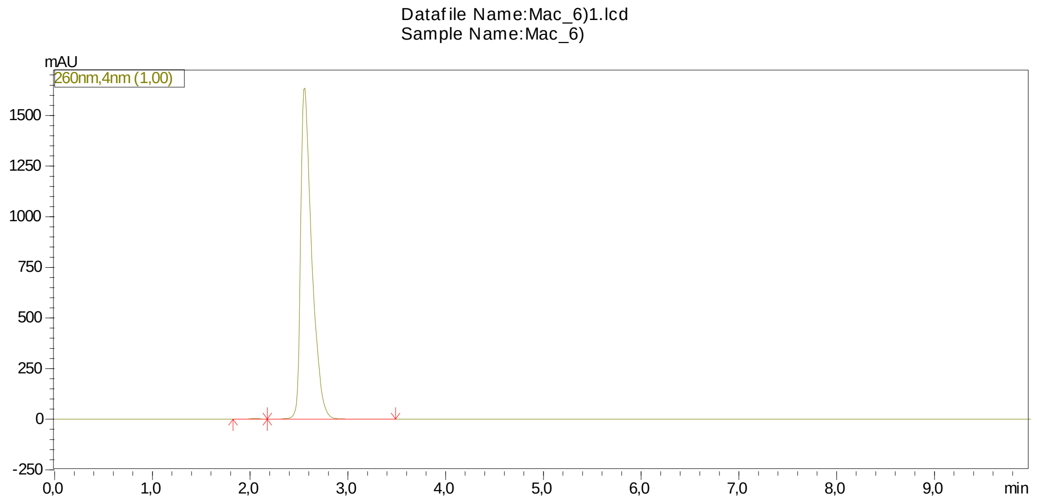

Peak#	Ret. Time	Area	Height	Conc.	Name	Area%
1	2,057	23363	3576	0,000		0,180
2	2,556	12953317	1635482	0,000		99,820
Total		12976681	1639059	0,000		100,000

Figure **S60**. HPLC chromatogram of compound **5a**

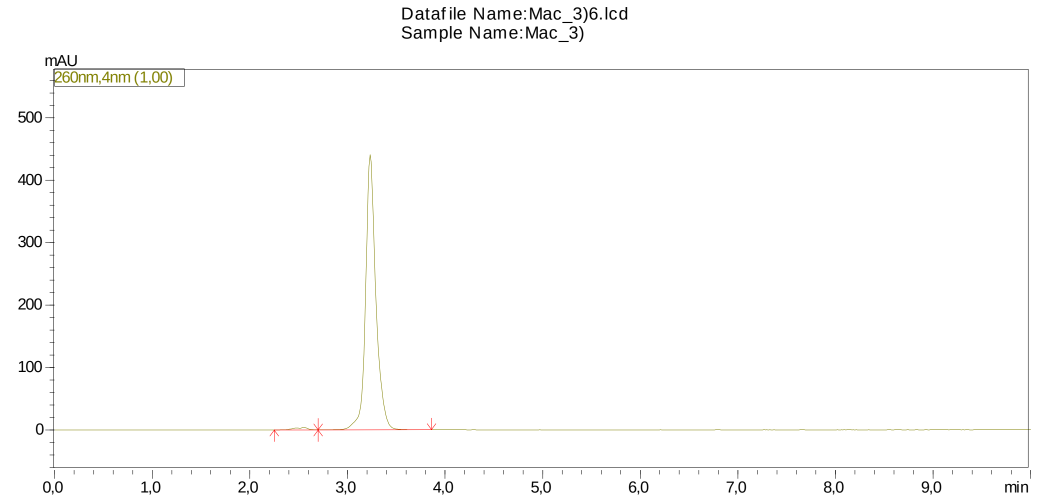

Peak#	Ret. Time	Area	Height	Conc.	Name	Area%
1	2,554	36978	3900	0,000		1,123
2	3,233	3256785	440860	0,000		98,877
Total		3293763	444760	0,000		100,000

Figure **S61**. HPLC chromatogram of compound **5b**

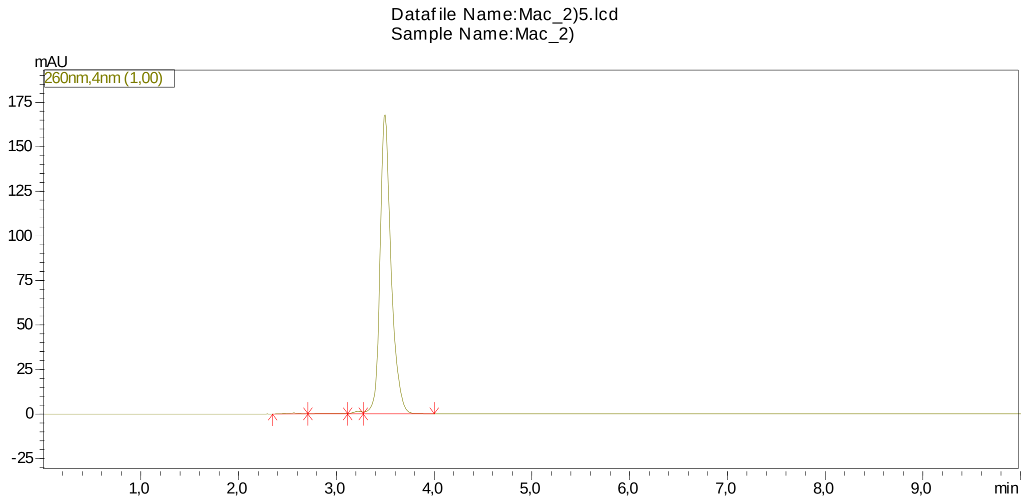

Peak#	Ret. Time	Area	Height	Conc.	Name	Area%
1	2,563	4407	563	0,000		0,329
2	2,987	5434	314	0,000		0,406
3	3,222	9115	1394	0,000		0,681
4	3,495	1320214	167829	0,000		98,584
Total		1339170	170100	0,000		100,000

Figure **S62**. HPLC chromatogram of compound **5c**

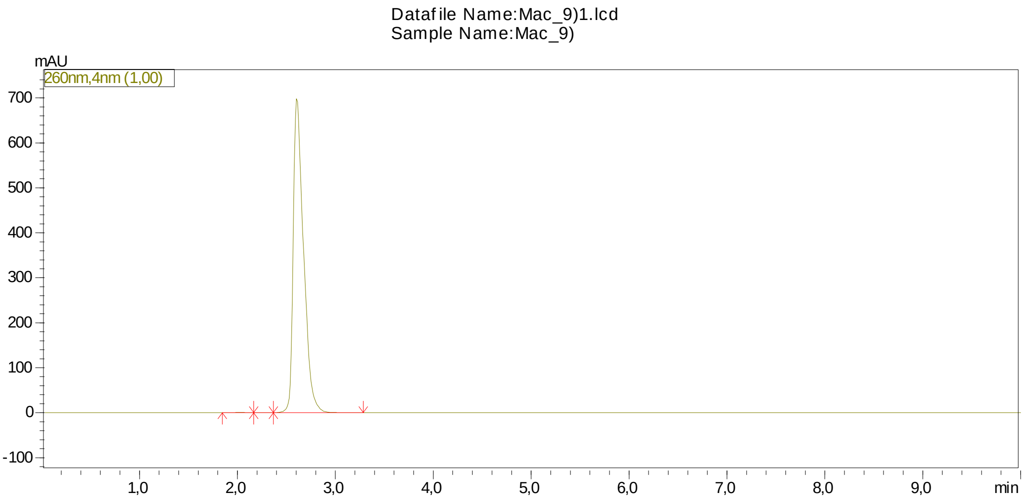

Peak#	Ret. Time	Area	Height	Conc.	Name	Area%
1	2,007	3772	422	0,000		0,074
2	2,293	1240	181	0,000		0,024
3	2,607	5099798	697670	0,000		99,902
Total		5104811	698273	0,000		100,000

Figure **S63**. HPLC chromatogram of compound **5f**

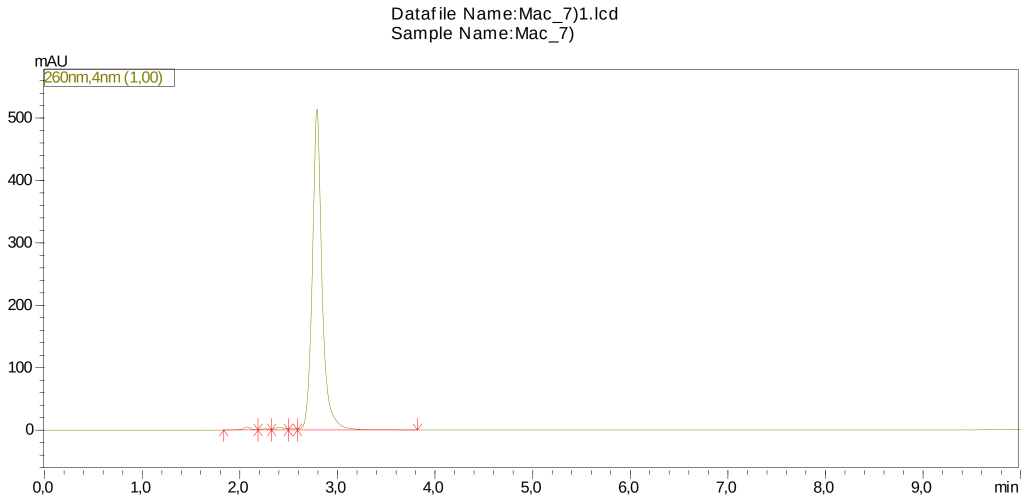

Peak#	Ret. Time	Area	Height	Conc.	Name	Area%
1	2,074	28923	4957	0,000		0,791
2	2,255	12384	1963	0,000		0,339
3	2,406	28758	4155	0,000		0,786
4	2,548	11170	2111	0,000		0,305
5	2,790	3575977	513864	0,000		97,779
Total		3657212	527051	0,000		100,000

Figure **S64**. HPLC chromatogram of compound **5g**

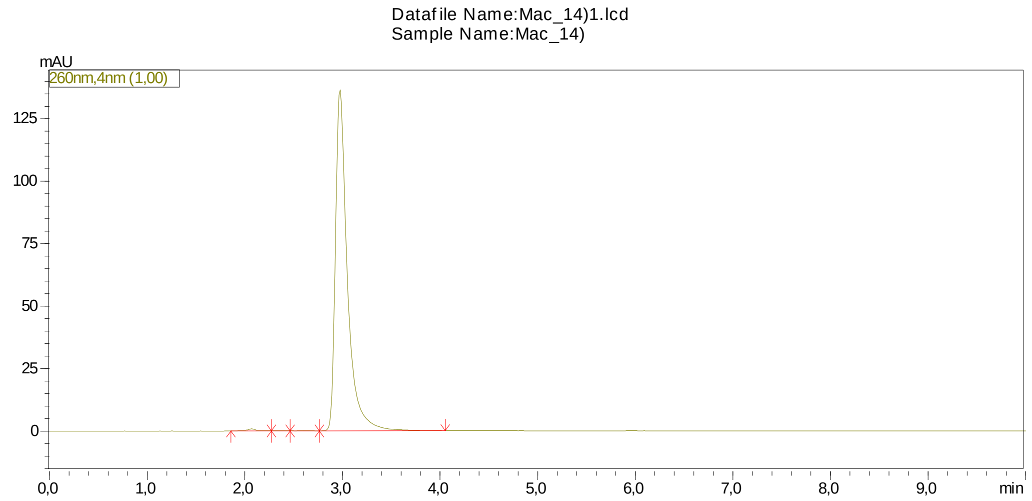

Peak#	Ret. Time	Area	Height	Conc.	Name	Area%
1	2,070	6499	862	0,000		0,557
2	2,383	1006	121	0,000		0,086
3	2,626	1413	180	0,000		0,121
4	2,974	1158389	136490	0,000		99,236
Total		1167307	137654	0,000		100,000

Figure **S65**. HPLC chromatogram of compound **8c**

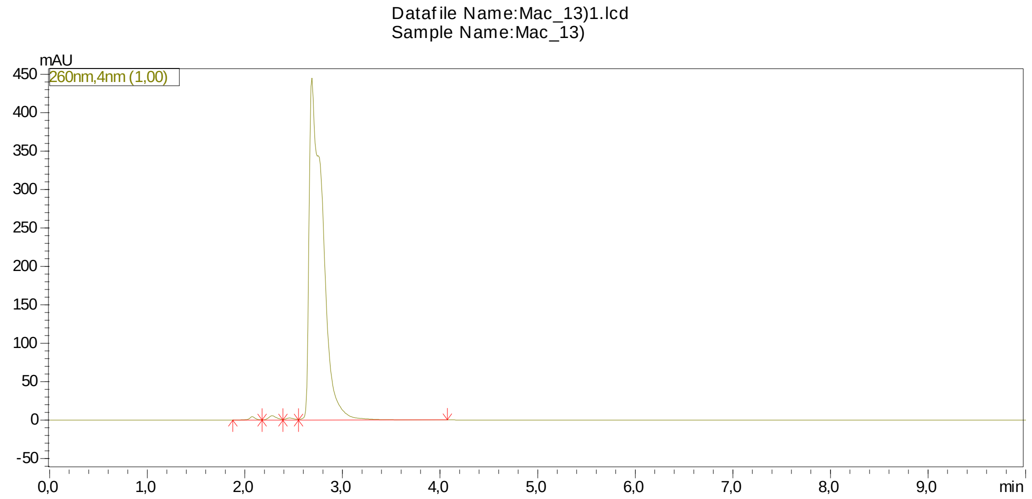

Peak#	Ret. Time	Area	Height	Conc.	Name	Area%
1	2,076	20533	4300	0,000		0,470
2	2,279	33450	5581	0,000		0,765
3	2,460	16928	2771	0,000		0,387
4	2,686	4299256	444788	0,000		98,377
Total		4370167	457440	0,000		100,000

Figure **S66**. HPLC chromatogram of compound **9a**

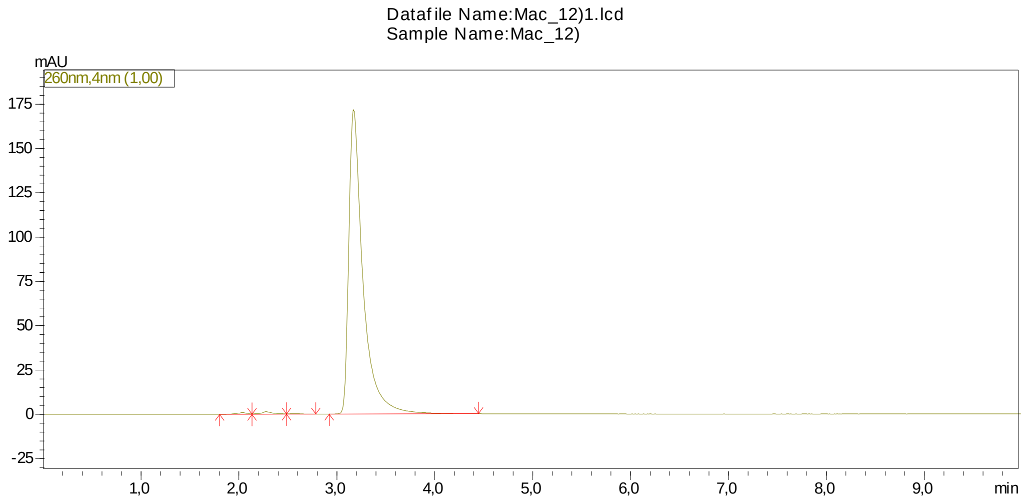

Peak#	Ret. Time	Area	Height	Conc.	Name	Area%
1	2,039	6774	899	0,000		0,386
2	2,277	11983	1407	0,000		0,682
3	2,566	2858	335	0,000		0,163
4	3,172	1734641	171773	0,000		98,769
Total		1756257	174413	0,000		100,000

Figure **S67**. HPLC chromatogram of compound **9b**

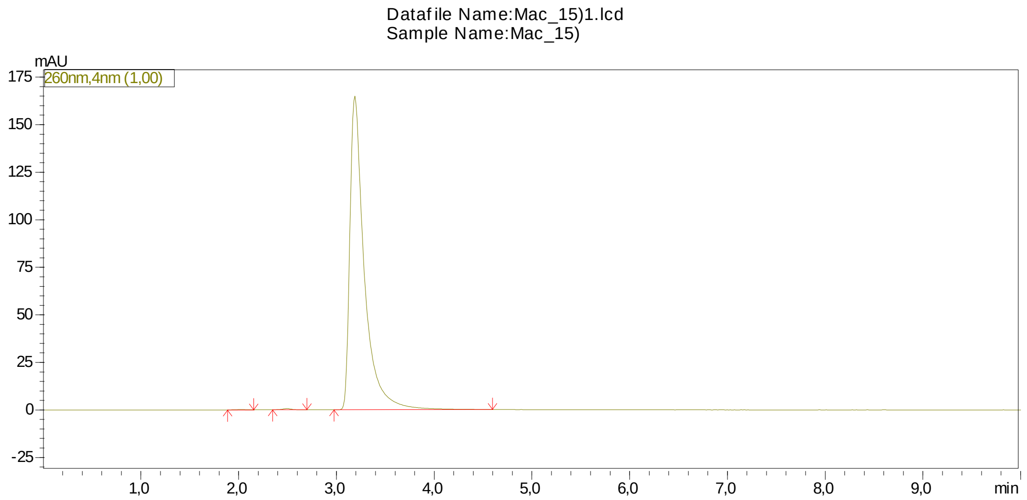

Peak#	Ret. Time	Area	Height	Conc.	Name	Area%
1	2,023	1654	230	0,000		0,097
2	2,493	4887	669	0,000		0,287
3	3,188	1698306	164995	0,000		99,616
Total		1704848	165894	0,000		100,000

Figure **S68** HPLC chromatogram of compound **11**

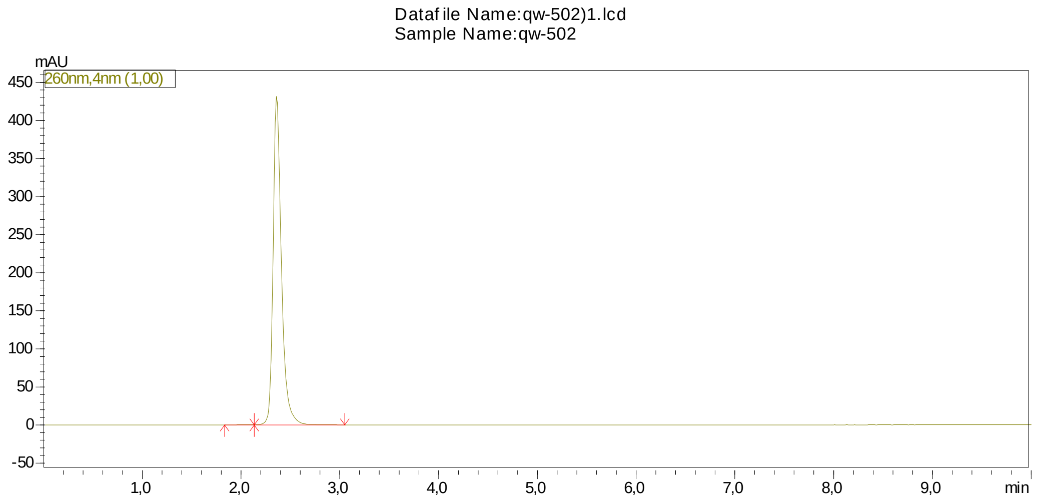

Peak#	Ret. Time	Area	Height	Conc.	Name	Area%
1	2,027	3339	358	0,000		0,131
2	2,361	2541580	431340	0,000		99,869
Total		2544919	431698	0,000		100,000

Figure **S69**. HPLC chromatogram of compound **13a**

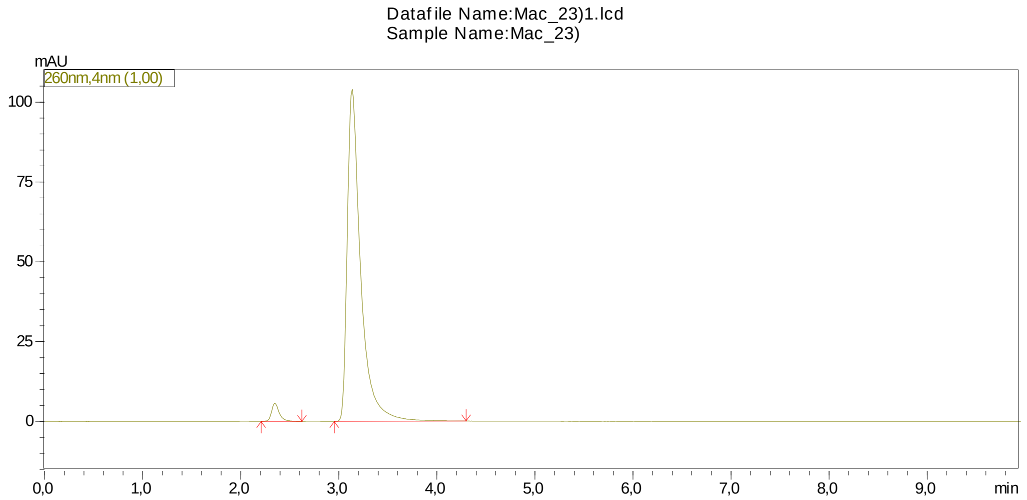

Peak#	Ret. Time	Area	Height	Conc.	Name	Area%
1	2,347	31104	5687	0,000		3,046
2	3,135	990044	103946	0,000		96,954
Total		1021148	109633	0,000		100,000

Figure **S70**. HPLC chromatogram of compound **13b**

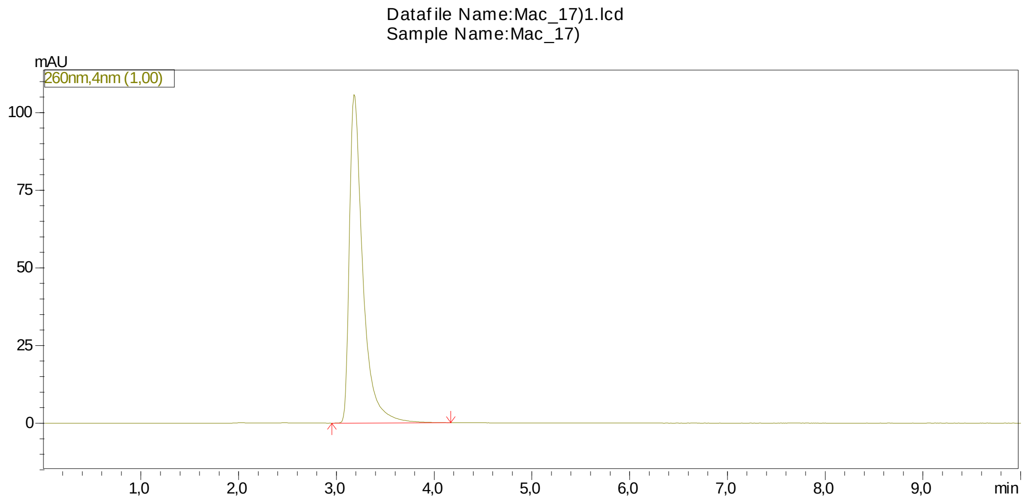

Peak#	Ret. Time	Area	Height	Conc.	Name	Area%
1	3,183	1012015	105755	0,000		100,000
Total		1012015	105755	0,000		100,000

Figure **S71**. HPLC chromatogram of compound **13c**

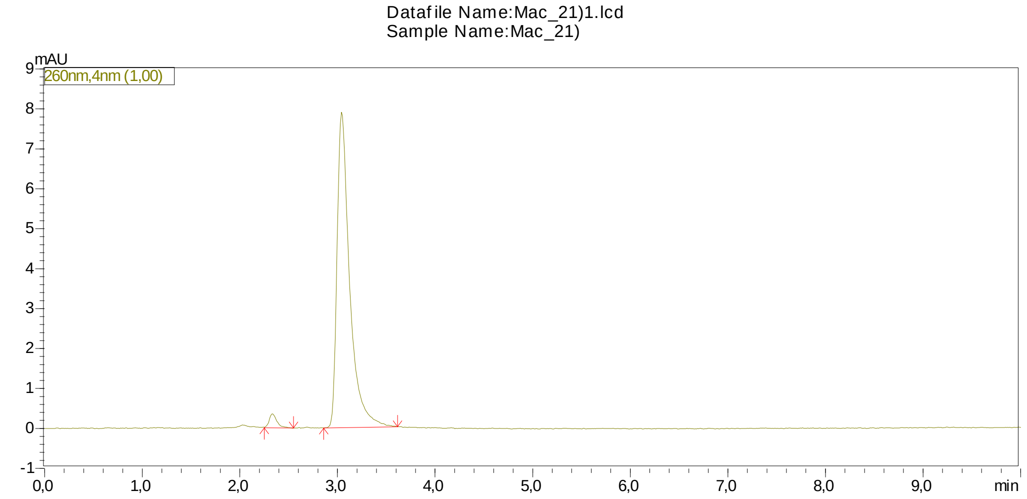

Peak#	Ret. Time	Area	Height	Conc.	Name	Area%
1	2,332	1950	349	0,000		2,667
2	3,043	71159	7896	0,000		97,333
Total		73108	8245	0,000		100,000

Figure **S72**. HPLC chromatogram of compound **13d**

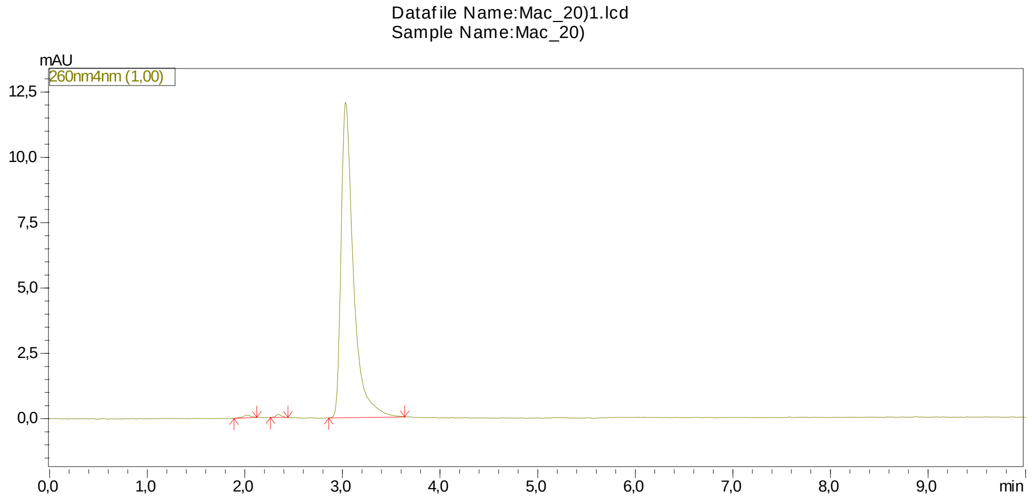

Peak#	Ret. Time	Area	Height	Conc.	Name	Area%
1	2,026	609	102	0,000		0,565
2	2,342	482	111	0,000		0,447
3	3,033	106726	12072	0,000		98,988
Total		107817	12285	0,000		100,000

Figure **S73**. HPLC chromatogram of compound **13e**

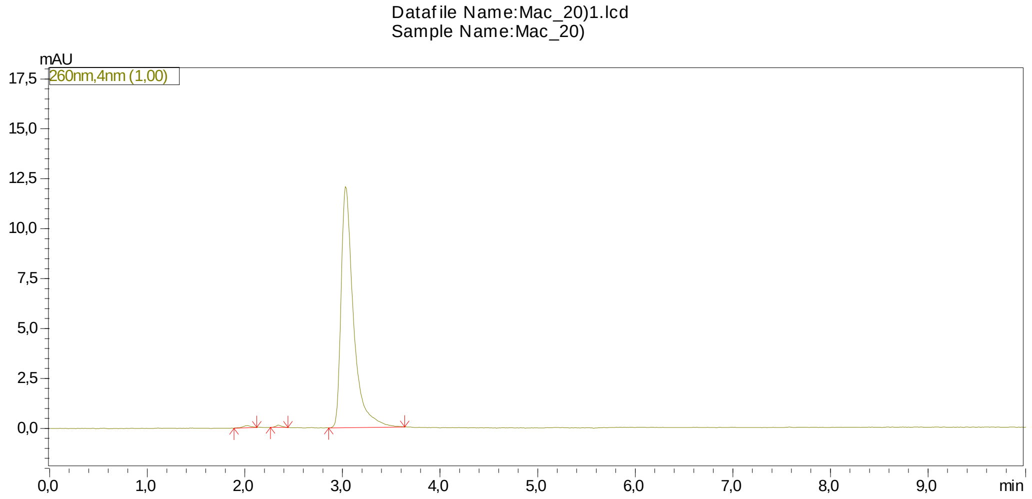

Peak#	Ret. Time	Area	Height	Conc.	Name	Area%
1	2,026	609	102	0,000		0,565
2	2,342	482	111	0,000		0,447
3	3,033	106726	12072	0,000		98,988
Total		107817	12285	0,000		100,000

Figure **S74**. HPLC chromatogram of compound **13f**

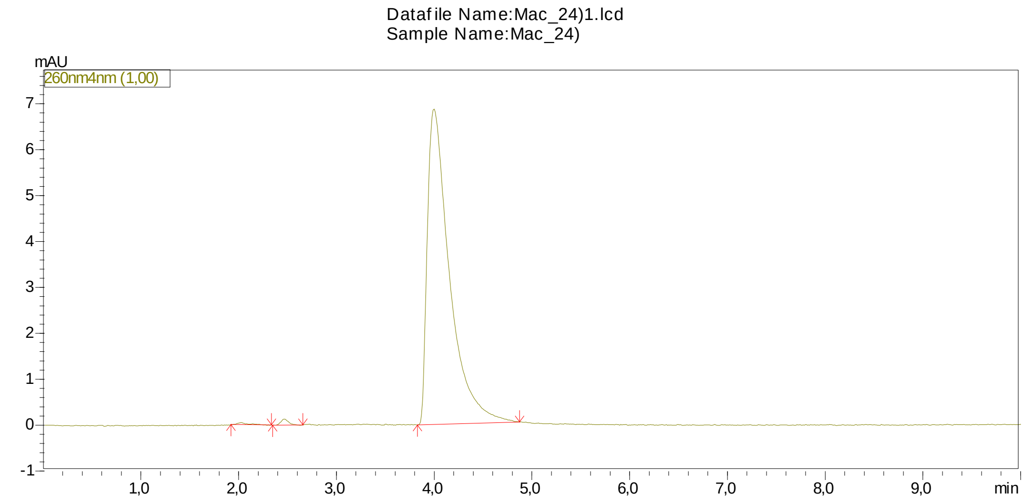

Peak#	Ret. Time	Area	Height	Conc.	Name	Area%
1	2,027	313	46	0,000		0,285
2	2,467	675	129	0,000		0,616
3	3,998	108563	6874	0,000		99,099
Total		109551	7050	0,000		100,000

Figure **S75**. HPLC chromatogram of compound **13g**

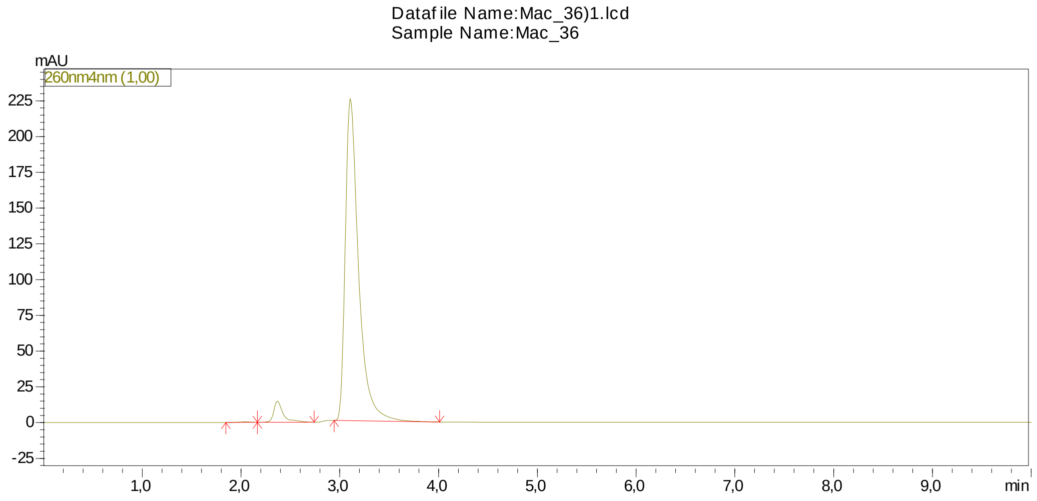

Peak#	Ret. Time	Area	Height	Conc.	Name	Area%
1	2,060	4171	453	0,000		0,189
2	2,368	100343	14886	0,000		4,545
3	3,107	2103193	225070	0,000		95,266
Total		2207708	240410	0,000		100,000

Figure **S76**. HPLC chromatogram of compound **13h**

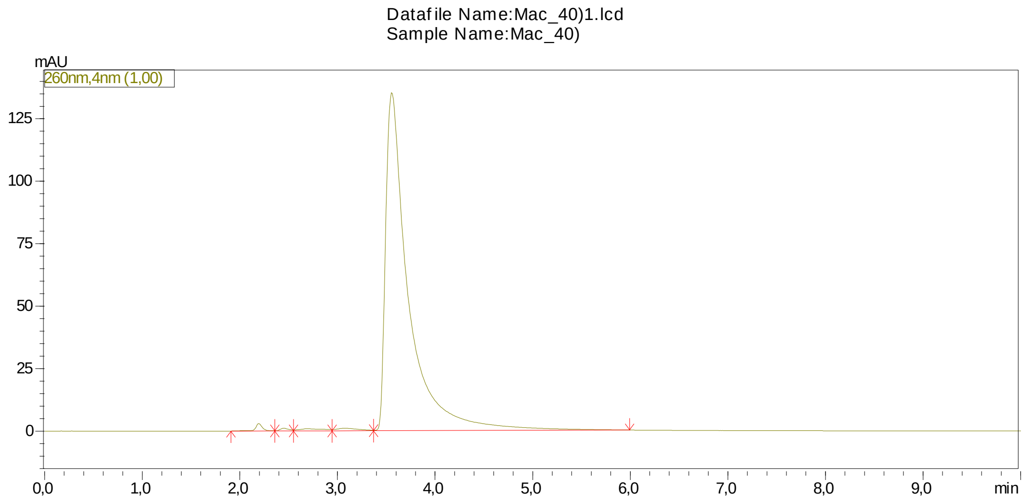

Peak#	Ret. Time	Area	Height	Conc.	Name	Area%
1	2,193	14981	2954	0,000		0,663
2	2,451	7019	1074	0,000		0,311
3	2,688	15100	888	0,000		0,669
4	3,073	15995	1013	0,000		0,708
5	3,556	2204769	135226	0,000		97,648
Total		2257864	141155	0,000		100,000

Figure **S77**. HPLC chromatogram of compound **13i**

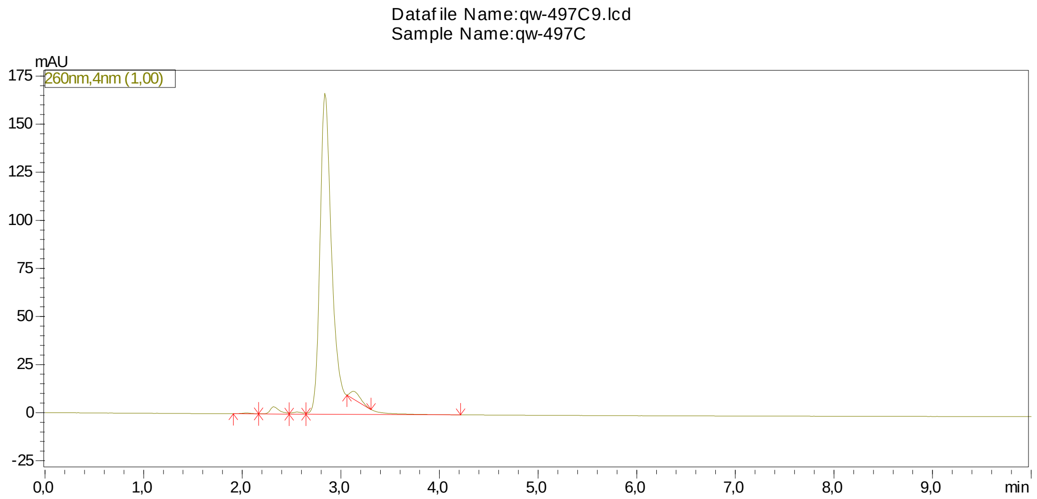

Peak#	Ret. Time	Area	Height	Conc.	Name	Area%
1	2,042	3340	455	0,000		0,214
2	2,319	25602	3756	0,000		1,639
3	2,555	7868	1045	0,000		0,504
4	2,839	1498621	166865	0,000		95,961
5	3,126	26262	3953	0,000		1,682
Total		1561693	176074	0,000		100,000

Figure **S78** HPLC chromatogram of compound **13j**

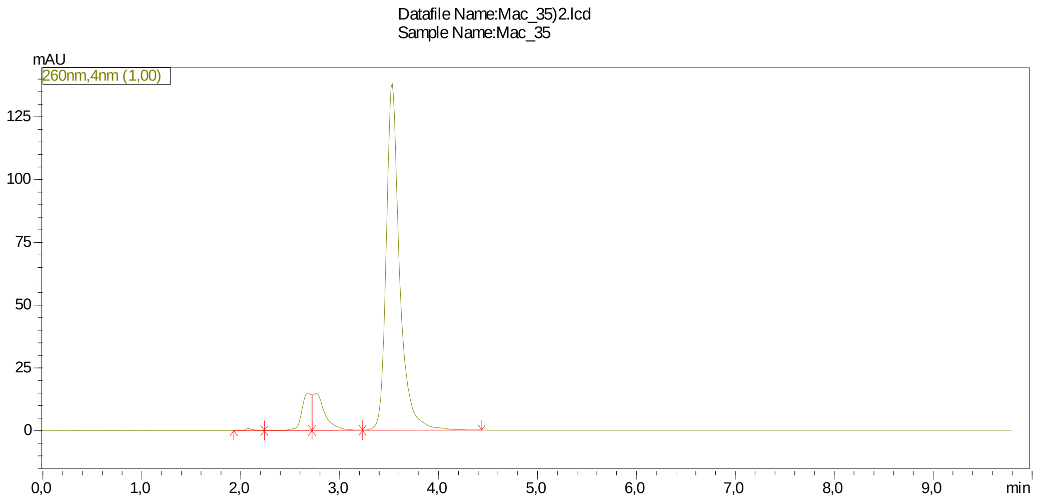

Peak#	Ret. Time	Area	Height	Conc.	Name	Area%
1	2,075	4007	742	0,000		0,262
2	2,678	102580	14879	0,000		6,704
3	2,764	123978	14676	0,000		8,102
4	3,529	1299644	138164	0,000		84,932
Total		1530208	168461	0,000		100,000

Figure **S79**. HPLC chromatogram of compound **13k**

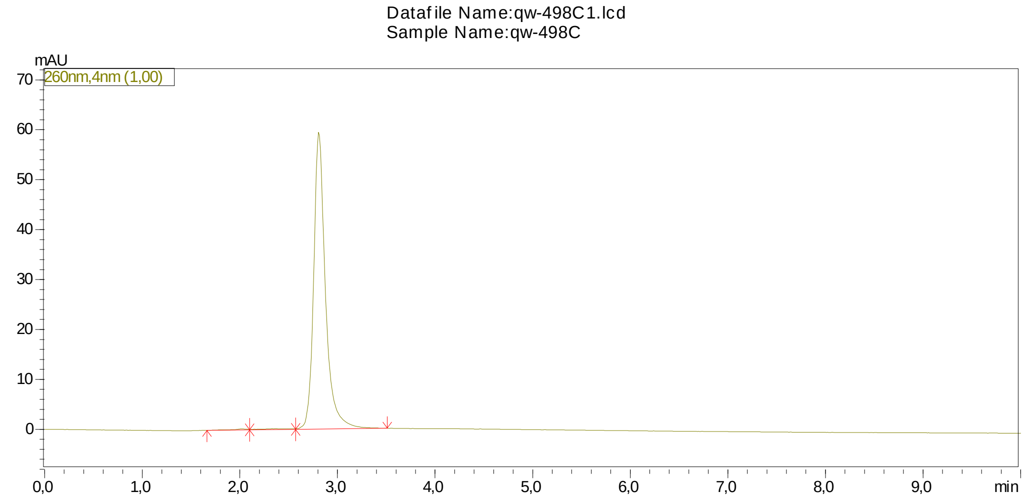

Peak#	Ret. Time	Area	Height	Conc.	Name	Area%
1	2,015	2128	194	0,000		0,421
2	2,325	3431	169	0,000		0,679
3	2,809	499824	59452	0,000		98,900
Total		505382	59815	0,000		100,000

Figure **S80**. HPLC chromatogram of compound **13l**

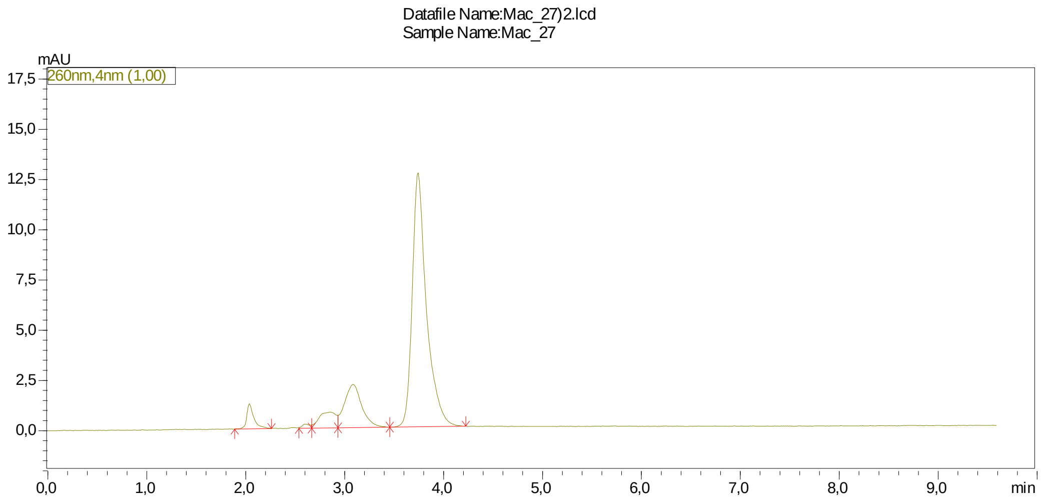

Peak#	Ret. Time	Area	Height	Conc.	Name	Area%
1	2,037	6272	1251	0,000		3,717
2	2,603	1150	201	0,000		0,681
3	2,856	9563	779	0,000		5,667
4	3,087	25795	2144	0,000		15,286
5	3,741	125965	12643	0,000		74,648
Total		168745	17018	0,000		100,000

Figure **S81**. HPLC chromatogram of compound **13m**

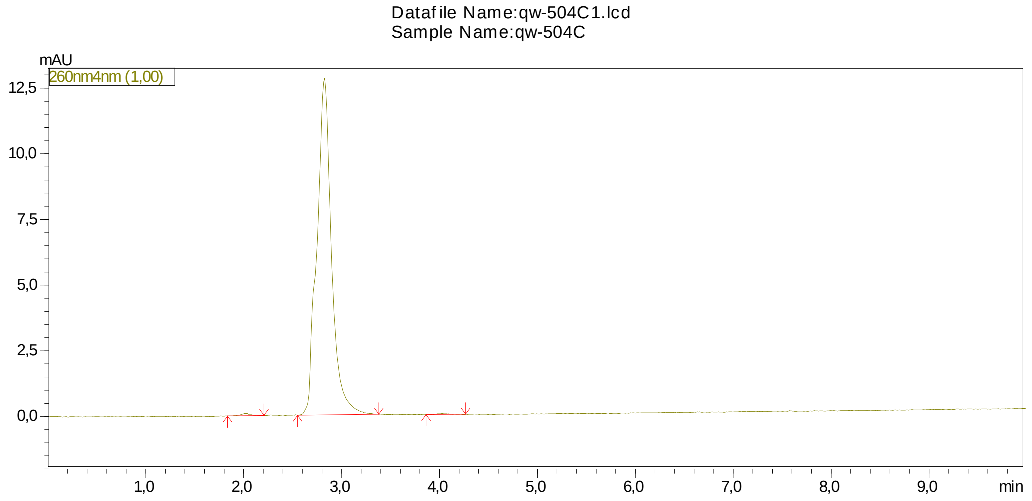

Peak#	Ret. Time	Area	Height	Conc.	Name	Area%
1	2,008	582	87	0,000		0,434
2	2,824	133246	12809	0,000		99,396
3	4,030	229	34	0,000		0,170
Total		134057	12930	0,000		100,000

Figure **S82**. HPLC chromatogram of compound **16**

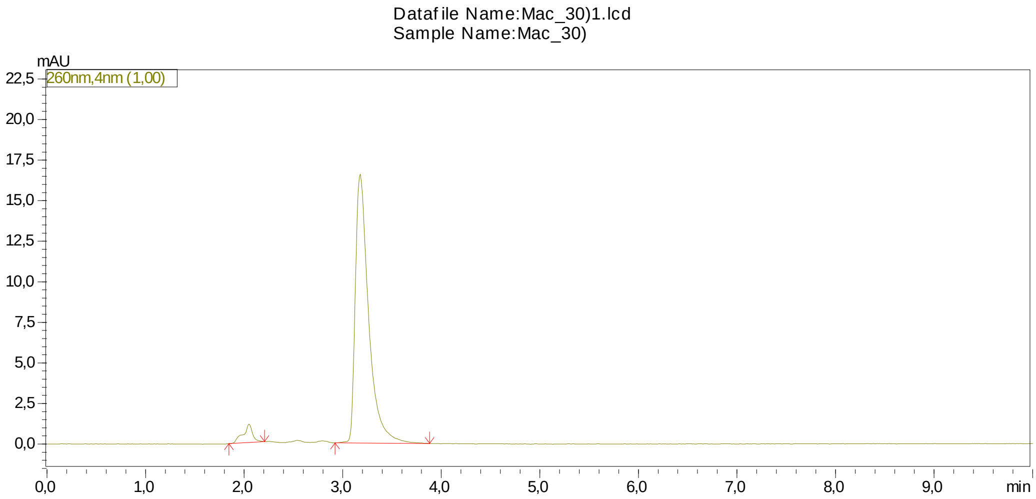

Peak#	Ret. Time	Area	Height	Conc.	Name	Area%
1	2,050	7708	1145	0,000		4,699
2	3,176	156311	16580	0,000		95,301
Total		164019	17725	0,000		100,000
